# Inhibin beta A drives colorectal cancer progression through macrophage M2 polarization and mitochondria-dependent ferroptosis suppression

**DOI:** 10.1038/s41392-025-02518-y

**Published:** 2025-12-26

**Authors:** Wentao Li, Lin Liang, Siyi Liu, Jingqiong Tang, Shuangyan Ou, Zhijun Yuan, Yanhong Zhou, Xia Yuan

**Affiliations:** 1 Gastroenterology and Urology Department Ⅱ, The Affiliated Cancer Hospital of Xiangya School of Medicine, Central South University/Hunan Cancer Hospital, Changsha, Hunan China; 2 NHC Key Laboratory of Carcinogenesis, Cancer Research Institute, Basic School of Medicine, Central South University, Changsha, Hunan China; 3 Department of Geriatrics, The Second Xiangya Hospital of Central South University, Changsha, Hunan China; 4 Key Laboratory of Carcinogenesis and Cancer Invasion of the Chinese Ministry of Education, Cancer Research Institute, Basic School of Medicine, Central South University, Changsha, Hunan China

**Keywords:** Gastrointestinal cancer, Tumour immunology

## Abstract

Colorectal cancer (CRC) is a prevalent malignant tumor, and its pathogenesis has not yet been fully elucidated. The tumor microenvironment (TME) and ferroptosis in cancer cells are key drivers of tumor progression and metastasis. This research revealed that elevated INHBA expression in CRC tissues correlates with unfavorable clinical outcomes. In vitro and in vivo studies demonstrated that elevated INHBA enhances CRC cellular growth, migration, and invasion, whereas INHBA knockdown inhibits these malignant biological behaviors. Further investigation revealed that INHBA drives malignancy by reprogramming tumor-associated macrophages (TAMs) toward the M2 phenotype in the TME and by inhibiting mitochondrial-dependent ferroptosis in CRC cells. Mechanistically, INHBA upregulates SLC25A10 to activate the succinate/SUCNR1 axis, thus facilitating M2-like TAM polarization. It also activates the mitochondrial glutathione (mtGSH)/glutathione peroxidase 4 (GPX4) pathway to suppress mitochondria-dependent ferroptosis in CRC cells. Additionally, INHBA acts as a scaffold protein to inhibit TRIM21-mediated ubiquitination and degradation of SLC25A10, thereby stabilizing the SLC25A10 protein. In summary, INHBA drives tumor progression by remodeling the immune microenvironment and antagonizing ferroptosis in CRC cells, providing a theoretical basis for developing INHBA-targeted inhibitors or combined immunoferroptosis therapeutic strategies.

**Figure:**
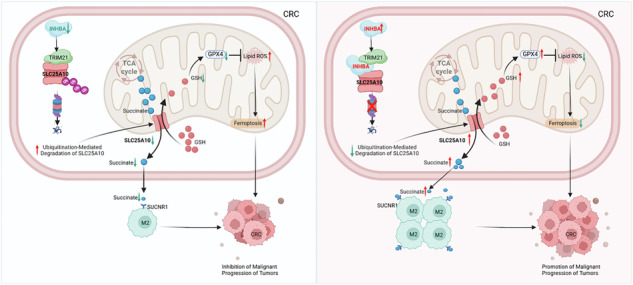
**Mechanisms of INHBA in colorectal cancer** In colorectal cancer, INHBA is upregulated. Acting as a scaffold protein, INHBA inhibits the K48-linked ubiquitination and degradation of the mitochondrial protein SLC25A10, mediated by the E3 ubiquitin ligase TRIM21. This inhibition leads to the upregulation of SLC25A10 expression. The upregulated SLC25A10 facilitates the transport of succinate from the mitochondrial matrix to the cytoplasm and further secretes it outside the tumor cells. The secreted succinate binds to SUCNR1 on macrophages, activating the succinate/SUCNR1 axis, which in turn promotes the M2 polarization of tumor-associated macrophages (TAMs). Meanwhile, SLC25A10, as one of the key mitochondrial glutathione (mtGSH) transporters embedded in the mitochondrial inner membrane, promotes the transport of glutathione (GSH) synthesized in the cytoplasm into the mitochondria. This process activates the mitochondrial GSH-GPX4 axis, thereby inhibiting mitochondrial ferroptosis. Through these two mechanisms, INHBA ultimately promotes the malignant progression of colorectal cancer

**Mechanisms of INHBA in colorectal cancer** In colorectal cancer, INHBA is upregulated. Acting as a scaffold protein, INHBA inhibits the K48-linked ubiquitination and degradation of the mitochondrial protein SLC25A10, mediated by the E3 ubiquitin ligase TRIM21. This inhibition leads to the upregulation of SLC25A10 expression. The upregulated SLC25A10 facilitates the transport of succinate from the mitochondrial matrix to the cytoplasm and further secretes it outside the tumor cells. The secreted succinate binds to SUCNR1 on macrophages, activating the succinate/SUCNR1 axis, which in turn promotes the M2 polarization of tumor-associated macrophages (TAMs). Meanwhile, SLC25A10, as one of the key mitochondrial glutathione (mtGSH) transporters embedded in the mitochondrial inner membrane, promotes the transport of glutathione (GSH) synthesized in the cytoplasm into the mitochondria. This process activates the mitochondrial GSH-GPX4 axis, thereby inhibiting mitochondrial ferroptosis. Through these two mechanisms, INHBA ultimately promotes the malignant progression of colorectal cancer

## Introduction

CRC ranks third among cancer deaths worldwide, with persistently high incidence and mortality rate.^1^ Therefore, CRC currently ranks among the most significant health system challenges in a multitude of nations.^2^ Aging populations and Western-style diets in affluent nations, coupled with established risk factors such as obesity and insufficient exercise, drive up the incidence of CRC.^3^ Despite significant advances in detection methods and treatment modalities including endoscopic and surgical local resection, local ablation therapy, targeted treatments, and immune interventions in recent years, the long-term prognosis for CRC patients remains low, especially in cases of advanced disease or metastasis.^3,4^ Therefore, in-depth investigations into the molecular processes of CRC, and the discovery of novel treatment targets and prognostic biomarkers, are highly important for improving the clinical outcomes of patients.

Inhibin beta A (INHBA) belongs to the transforming growth factor-β (TGF-β) superfamily and was initially discovered for its role in the reproductive system.^5,6^ For instance, INHBA is instrumental in testicular development and function,^7^ and modulates ovarian tissue and oocyte growth.^8^ Recently, INHBA has emerged as a focal target because of its upregulation in various tumors and its significant role in tumor progression.^9–16^ As a key ligand for TGF-β signaling, INHBA can activate the TGF-β pathway, engage the phosphatidylinositol-3-kinase/protein kinase B (PI3K/Akt) pathway, increase cyclin D1 expression, and accelerate the cell-cycle progression, thereby driving CRC cell proliferation.^17^ Additionally, silencing the INHBA gene can inhibit the proliferation, migration, and invasion of gastric cancer cells and osteosarcoma cells by suppressing the TGF-β signaling pathway.^18,19^ Nevertheless, the precise mechanisms underlying INHBA in CRC, particularly in terms of tumor immunity and modes of cell death, remain unclear.

The TME encompasses the non-malignant cells and structural elements found within a tumor, along with the bioactive factors they generate and secrete.^20^ The ongoing interplay between cancer cells and their surrounding milieu is instrumental in driving tumorigenesis, progression, and metastatic dissemination.^21,22^ TAMs serve as fundamental elements within the TME, emerging as a pivotal regulator in cancer progression and an attractive therapeutic target. These immune cells can be broadly classified into two subsets: M1-type and M2-type TAMs.^23^ The M2-type TAMs are closely related to tumor immune suppression, angiogenesis, and invasive growth.^24–27^ Succinate, a circulating metabolite, serves as the sole direct bridge connecting the Krebs cycle and the mitochondrial respiratory chain. And it contributes significantly to inflammation, hypoxia, and metabolic processes.^28–30^ Succinate is generated in the mitochondrial matrix, and its efflux into the cytoplasm serves as a signal of mitochondrial status.^29^ Succinate receptor (SUCNR1), otherwise known as GPR91, belongs to the G protein-coupled receptor family. This receptor is a key node in cellular metabolic pathways, critically modulates immune homeostasis, and orchestrates inflammatory responses.^31^ Studies have shown that tumor-derived succinate can activate the SUCNR1 on macrophages, polarizing them into M2-type TAMs and thereby promoting oncogenic signaling.^32^ Solute carrier family 25 member 10 (SLC25A10), alternatively termed the mitochondrial dicarboxylate ion carrier (DIC), is a mitochondrial transporter which primarily facilitates mitochondrial metabolite exchange. SLC25A10 critically maintains TCA cycle intermediate homeostasis and modulates redox equilibrium and ferroptosis susceptibility.^33,34^ Studies have shown that SLC25A10 can transport succinate from the mitochondrial matrix to the cytoplasm.^35,36^ On the other hand, mitochondria serve as the primary hubs for oxygen utilization and the generation of reactive oxygen species (ROS) in cells, with the majority of ROS originating from the mitochondrial respiratory chain. mtGSH acts as a critical line of defense in preserving redox balance within mitochondria, helping to stave off or fix mitochondrial impairments triggered by oxidative stress and thus preventing cellular demise.^37^ Since mitochondria do not produce GSH, SLC25A10 can mediate the import of GSH into the mitochondria, thereby participating in the regulation of redox homeostasis and ferroptosis.^38^ Tripartite motif-containing protein 21 (TRIM21), commonly referred to as Ro52, functions as an E3 ubiquitin ligase, utilizing its RING domain to catalyze ubiquitin transfer. It plays a pivotal role in numerous cellular processes, including immune regulation, inflammation control, cell death, and tumor progression.^39–42^

This research sought to delineate the mechanisms by which INHBA operates in CRC, particularly its roles in regulating the TME and cellular metabolism, as well as the mode of cell death. We found that INHBA, SLC25A10, and TRIM21 are interacting proteins. INHBA acts as a scaffold protein to prevent TRIM21 from degrading SLC25A10 through K48-linked ubiquitination, thereby boosting its stability. Moreover, INHBA boosts the succinate/SUCNR1 pathway to induce M2 TAM polarization by enhancing the SLC25A10 protein levels. It also activates the mtGSH/GPX4 axis, which in turn suppresses mitochondria-dependent ferroptosis. Collectively, these alterations ultimately fuel the aggressive growth of CRC. Our findings not only comprehensively reveal multiple mechanisms of INHBA in CRC progression but also identify candidate targets to advance the development of new therapeutic strategies.

## Results

### INHBA is elevated in colorectal cancer and positively tracks with clinical progression and poor prognosis

**Fig. 1:**
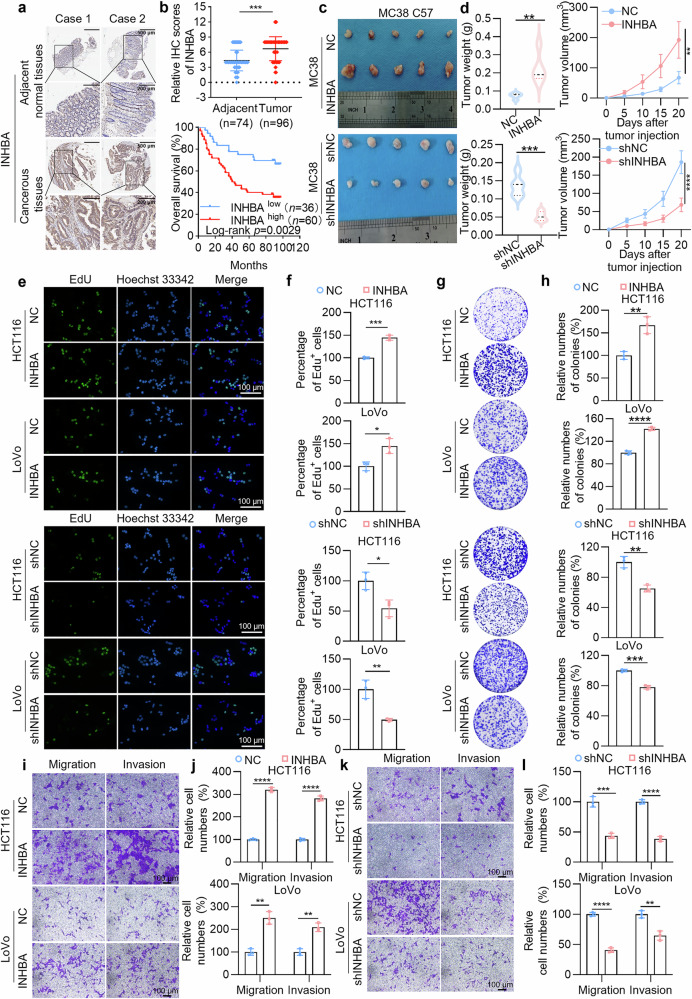
INHBA upregulation in CRC correlates with advanced disease and adverse prognosis. **a** Representative IHC images of INHBA in 96 CRC tissues and 74 matched normal adjacent tissues. Scale bar: 500 μm (top), 200 μm (bottom). **b** INHBA expression in CRC vs adjacent normal tissue (top) and Kaplan–Meier OS curves (bottom; log-rank test). **c** In vivo tumorigenesis experiments: Subcutaneous tumor implantation experiments in C57BL/6 mice using MC38 cells stably overexpressing or knocking down INHBA. Photographs of tumors removed from euthanized mice. **d** Tumor growth curve (measured every 5 days) and final weight (day 20); *n* = 5. **e** Representative EdU proliferation images. Scale bar: 100 μm. **f** Quantification of EdU-positive cells (*n* = 3). **g** Representative colony images. **h** Colony number quantification (*n* = 3). **i** Representative images of migration and invasion assays with INHBA overexpression. Scale bar: 100 μm. **j** Quantification of migrated/invaded cells (*n* = 3). **k** Representative migration and invasion images after INHBA knockdown. Scale bar: 100 μm. **l** Quantification of migrated/invaded cells (*n* = 3). For all the statistical plots, values are expressed as the means ± SD. *P < 0.05, **P < 0.01, ***P < 0.001, ****P < 0.0001

First, we identified differentially expressed genes that are upregulated in CRC via the TCGA and GEO databases. We subsequently used the VennDiagram package in R to create Venn diagrams of the intersections and unions of the four datasets. The results revealed that 16 genes were consistently upregulated across these datasets (Supplementary Fig. 1a). Next, we analyzed the overall survival (OS) and disease-free survival (DFS) rates associated with these 16 genes via the GEPIA web server (http://gepia.cancer-pku.cn/index.html). The analysis revealed that only high INHBA expression robustly predicted shortened OS and DFS in patients with colon adenocarcinoma (COAD) (Supplementary Fig. 1b, c). Furthermore, we used a human colon tissue microarray to probe INHBA expression levels in malignant colon tissue versus healthy surrounding tissue. The findings revealed a marked upregulation of INHBA expression within the malignant samples. K‒M survival analysis revealed a correlation between elevated INHBA levels and unfavorable outcomes in CRC patients (Fig. 1a, b, and Supplementary Table 1). To further evaluate the prognostic value of INHBA expression in CRC patients, we constructed receiver operating characteristic (ROC) curves for OS at 1, 3, and 5 years on the basis of tissue microarray data. The area under the curve (AUC) values were 0.798, 0.717, and 0.742, respectively, indicating that INHBA expression has favorable prognostic predictive potential (Supplementary Fig. 2). Additionally, correlation analysis of INHBA expression with clinicopathological features in CRC patients showed a significant link to T stage (Supplementary Table 2). When evaluating OS among CRC patients, the findings of Cox proportional-hazards regression, both univariate and multivariate, revealed that INHBA expression levels significantly predicted OS. According to the univariate analysis, the high-expression group had an increased risk relative to the low-expressors. In the multivariate analysis, INHBA expression levels remained significantly associated with OS even after adjusting for other variables (Supplementary Table 3). In summary, these results demonstrate that INHBA is overexpressed in CRC and is tightly linked to adverse patient outcomes.

To assess INHBA’s oncogenic function in CRC in vivo, we first infected MC38 cells with lentivirus and confirmed the successful generation of INHBA-overexpressing and INHBA-knockdown models via qPCR and Western blot experiments (Supplementary Fig. 3a, b). We subsequently performed subcutaneous syngeneic tumor transplantation experiments in C57BL/6 mice via these methods. The results revealed that tumors in the INHBA-overexpressing cohort exhibited significantly greater size, volume, and weight relative to the control cohort. In contrast, the INHBA-knockdown cohort presented the opposite results (Fig. 1c, d). Additionally, we assessed the cell proliferation index in these solid tumors. Relative to the control, Ki-67 signal was significantly increased in the INHBA-overexpressing cohort, whereas it was substantially reduced in the INHBA-knockdown cohort (Supplementary Fig. 4a, b). These results reinforce INHBA’s oncogenic function in CRC.

Furthermore, we uncovered how INHBA shapes the phenotypic identity of CRC cells. We selected two normal colon mucosal epithelial cell lines and five colon adenocarcinoma cell lines for Western blot analysis. The findings showed markedly elevated INHBA levels in CRC cells versus normal colon mucosal epithelial cells (Supplementary Fig. 5). We subsequently selected two INHBA-high cell lines (HCT116 and LoVo) for further experiments and constructed CRC cell lines in which INHBA was overexpressed or knocked down (Supplementary Fig. 6a, b). EdU cell proliferation assay indicated that INHBA overexpression markedly increased the proliferative capacity of CRC cells, whereas INHBA knockdown inhibited cell proliferation (Fig. 1e, f). Colony-formation assays yielded consistent outcomes (Fig. 1g, h). INHBA overexpression notably augmented the migration and invasion of HCT116 and LoVo cells, as evidenced by these assays, while its depletion attenuated both processes (Fig. 1i–l). Western blot analysis revealed that INHBA overexpression substantially decreased the epithelial markers E-cadherin and ZO-1 but markedly increased the mesenchymal markers vimentin and N-cadherin in CRC cells; conversely, INHBA knockdown produced the opposite effects (Supplementary Fig. 7a, b).

In summary, these findings establish INHBA as a pivotal driver of CRC cell proliferation, migration, and invasion.

### INHBA promotes the M2 polarization of TAMs in the tumor microenvironment

To elucidate how INHBA promotes the malignant progression of CRC, we used 24 immune cell signature genes defined by Bindea et al. (Immunity, 2013).^43^ By applying ssGSEA to the TCGA-COAD dataset, we quantified immune-subpopulation infiltration levels and performed unsupervised hierarchical clustering to classify samples into high- and low-immune infiltration cohorts. The resulting heatmap revealed that the low-infiltration subset was enriched for late N stage, M stage and overall stage, implying that insufficient immune infiltration is associated with accelerated tumor progression. Moreover, the Mann‒Whitney U test revealed that INHBA expression rose as immune infiltration declined, suggesting that elevated INHBA may reflect an immune-escape phenotype and is closely linked to an immunosuppressive microenvironment (Supplementary Fig. 8a, b). We subsequently utilized CIBERSORTx (https://cibersortx.stanford.edu/) to evaluate tumor-infiltrating immune cells abundance. Analyses unveiled that INHBA expression levels showed strong concordance with several immune cell types, including macrophages (Supplementary Fig. 9a). On the basis of the median INHBA expression, samples were stratified into INHBA-high and INHBA-low cohorts. Differential analysis of macrophage infiltration abundance between these two groups revealed substantially greater macrophage infiltration in INHBA-high cohorts than in INHBA-low cohorts (Supplementary Fig. 9b). Macrophages are important immune cells. In the TME, macrophages tend to shift to TAMs with special functions, most of which exhibit features similar to those of M2-type macrophages, thereby promoting tumor development. Analysis using TIMER (http://cistrome.shinyapps.io/timer/) revealed that INHBA levels showed a positive association with M2-type TAM marker expression, such as CD163 (*P* < 2.2e−16, *R* = 0.57) and MRC1 (CD206) (*P* < 2.2e−16, *R* = 0.55) (Supplementary Fig. 9c). We next performed multiplex immunofluorescence staining on tissue sections from 93 patients with colon cancer to examine the association of INHBA tumor expression with macrophage infiltration. INHBA⁺ cell counts correlated positively with CD68⁺ macrophage density (*R* = 0.5304, *P* < 0.0001) (Supplementary Fig. 10a, b). Using the median fluorescence intensity of INHBA within tumor nests as the cutoff, we classified samples into INHBA_high (*n* = 47) and INHBA_low (*n* = 46) groups and quantified the fraction of CD68⁺ macrophages in three concentric peritumoral zones (0–20 μm, 20–30 μm, and 30–50 μm) surrounding individual tumor cells. Compared with INHBA_low tumors, INHBA_high tumors presented a significantly greater proportion of CD68⁺ macrophages in each zone (Supplementary Fig. 10c, d), suggesting that high INHBA expression recruits CD68⁺ macrophages into the TME and promotes their peritumoral accumulation, thereby fostering an immunosuppressive, tumor-promoting niche.

**Fig. 2:**
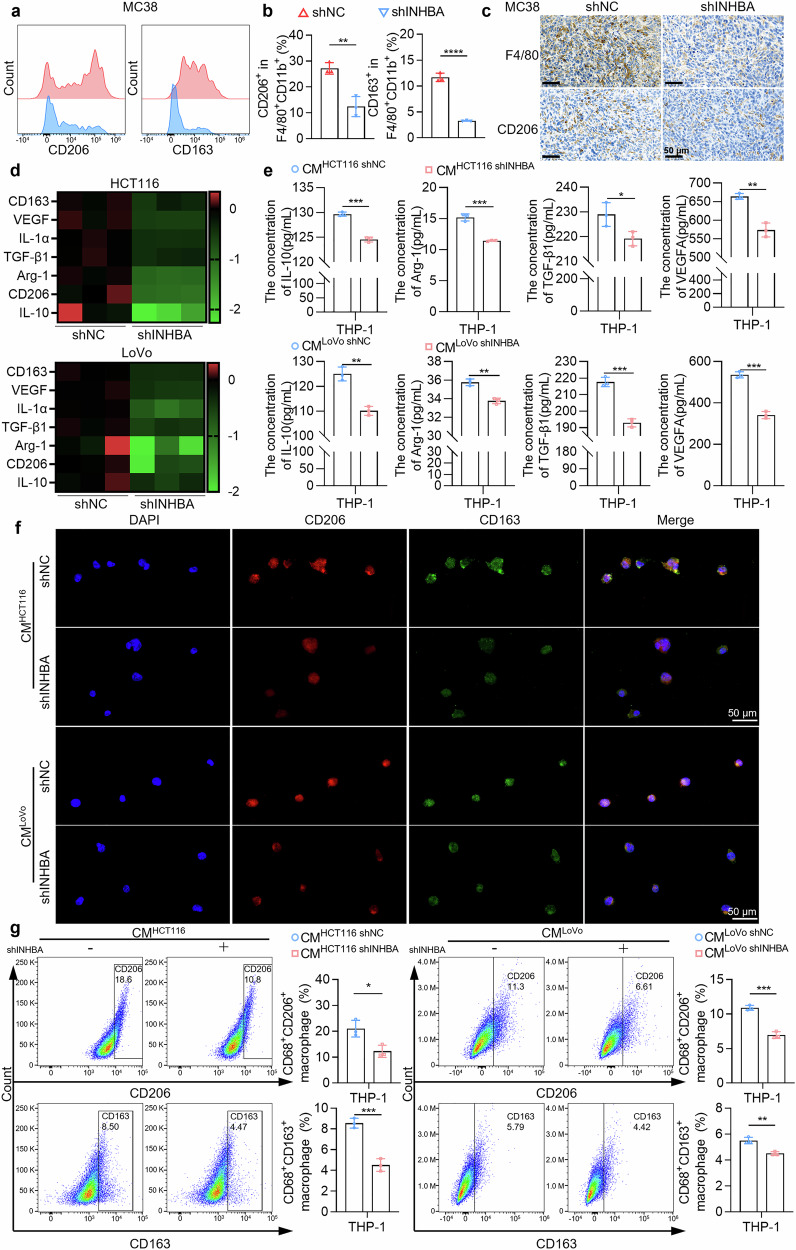
INHBA silencing inhibits TAM M2 polarization within the TME. **a** Representative flow-cytometry plots showing M2-like (F4/80⁺CD11b⁺CD206⁺ and F4/80⁺CD11b⁺CD163⁺) TAMs after INHBA knockdown in MC38 syngeneic tumors. **b** Quantification of M2-like TAM proportions after INHBA knockdown. **c** IHC revealing INHBA expression association with F4/80 and CD206 in murine tissue. Scale bar: 50 µm. **d** qPCR analysis of M2-polarization gene expression in TAMs after treatment with CM from human CRC cells with INHBA knockdown. Data are log2-transformed. **e** ELISA quantification of IL-10, Arg-1, TGF-β1, and VEGFA levels in human macrophages treated with different CMs. **f** Immunofluorescence showing CD206 (red) and CD163 (green) in human macrophages treated with different CMs. Scale bar: 50 µm. **g** Flow-cytometric quantification of CD68⁺CD206⁺ and CD68⁺CD163⁺ macrophages subsets after different CM treatments. For all the statistical plots, values are expressed as the means ± SD; *n* = 3 independent experiments. *P < 0.05, **P < 0.01, ***P < 0.001, ****P < 0.0001

Furthermore, we performed flow cytometry analysis on CRC tumor grafts from INHBA-overexpressing and INHBA-knockdown mice. Results showed that INHBA knockdown significantly decreased total macrophage infiltration, whereas INHBA overexpression increased infiltration (Supplementary Fig. 11a, b). Furthermore, knockdown of INHBA significantly reduced the F4/80⁺CD11b⁺CD206⁺ and F4/80⁺CD11b⁺CD163⁺ macrophages percentage (Fig. 2a, b), whereas elevated INHBA levels raised these cells’ percentage (Supplementary Fig. 12a, b). Similarly, immunohistochemistry (IHC) analysis revealed decreased expression of F4/80 and CD206 upon INHBA knockdown (Fig. 2c), whereas overexpression of INHBA led to increased expression of these markers (Supplementary Fig. 12c). These in vivo experimental results indicate that INHBA promotes TAMs’ M2 polarization within the TME. Moreover, immunohistochemical staining revealed elevated expression of CD8, GZMB, and CD11c in tumor tissues upon INHBA knockdown (Supplementary Fig. 13), indicating that silencing INHBA may enhance CD8⁺ T cells and dendritic cells (DCs) infiltration within the TME. These data establish INHBA as a candidate target for immune-based therapy.

We prepared conditioned medium (CM) from CRC cell lines as described in the Materials and methods section and used it to stimulate THP-1 cells differentiated with phorbol 12-myristate 13-acetate (PMA; 100 ng/mL) for 24 hours. We subsequently detected M2 polarization-related genes in TAMs via qPCR. In macrophages exposed to CM from INHBA-knockdown cells (HCT116 and LoVo), the mRNA levels of genes such as CD163, VEGF, IL-1α, TGF-β1, Arg-1, CD206, and IL-10 were significantly reduced (Fig. 2d). In contrast, CM from INHBA-overexpressing cells led to increased expression of these genes (Supplementary Fig. 12d). Additionally, we measured IL-10, Arg-1, TGF-β1, and VEGFA levels in macrophages treated with different CMs via enzyme-linked immunosorbent assay (ELISA). Macrophages exposed to CM from the INHBA-knockdown group had significantly reduced secretion levels of the above factors (Fig. 2e), whereas those in the INHBA-overexpressing group presented the opposite effect (Supplementary Fig. 12e).

We subsequently monitored the polarization phenotype of the THP-1 cells via immunofluorescence. CM from the INHBA-knockdown group markedly inhibited the M2 polarization of THP-1 cells relative to the control, as evidenced by the reduced fluorescence intensity of CD206 (red fluorescence) and CD163 (green fluorescence) (Fig. 2f). In contrast, INHBA overexpression had the opposite effect (Supplementary Fig. 12f). Flow cytometry experiments also yielded similar results, revealing a decreased proportion of CD68⁺CD206⁺ macrophages and CD68⁺CD163⁺ macrophages in the INHBA-knockdown group (Fig. 2g), whereas CM from the INHBA-overexpressing group significantly promoted the M2 polarization of THP-1 cells (Supplementary Fig. 12g). Furthermore, we repeated the above experiments via a transwell coculture system and found that the trends were consistent with those observed upon direct CM stimulation (Supplementary Figs. 14, 15). In summary, these experimental results indicate that INHBA can foster M2-like differentiation of TAMs in the TME.

Next, CM from CRC cell lines engineered to overexpress or knock down INHBA was used to treat THP-1 monocytes that had differentiated into macrophages after 24 h of PMA exposure. The resulting macrophages were then labeled with PKH26 and cocultured with CFDA SE-labeled HCT116 or LoVo cells, and tumor cell phagocytosis was quantified via flow cytometry. The phagocytic activity of macrophages cultured with INHBA-knockdown CM was significantly increased, whereas that of those cultured with INHBA-overexpressing CM was markedly reduced (Supplementary Fig. 16a, b). These results demonstrate that INHBA-driven M2 polarization of TAMs attenuates their capacity to phagocytose tumor cells.

### INHBA promotes M2 polarization of TAMs by activating the succinate/SUCNR1 axis

**Fig. 3:**
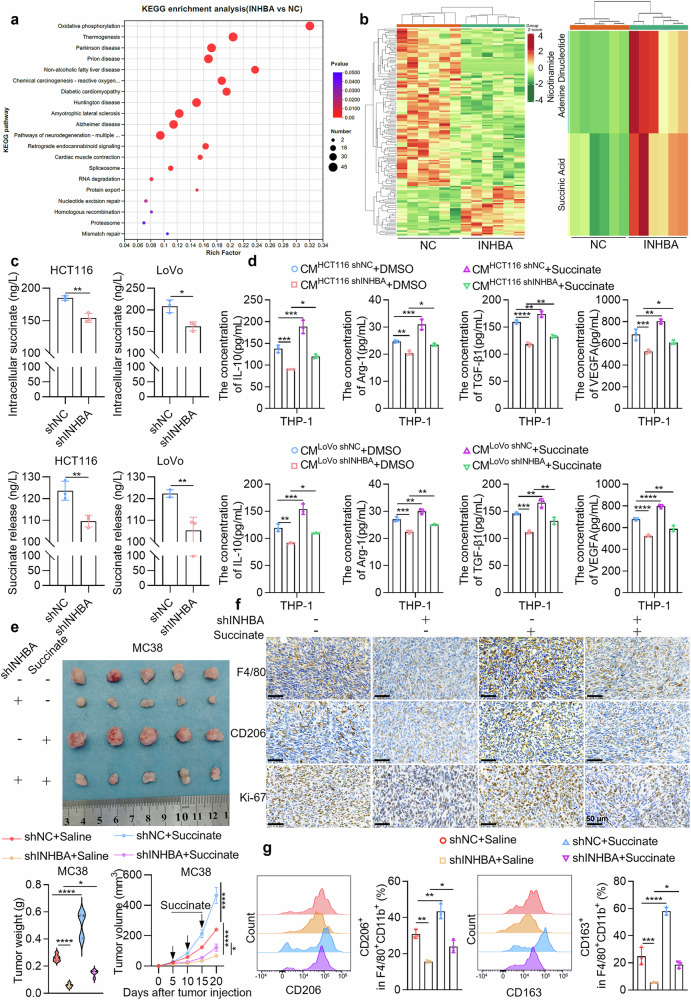
INHBA drives TAMs toward M2 phenotype via the succinate/SUCNR1 pathway activation. **a** KEGG enrichment of RNA-seq data. **b** Results of widely targeted metabolomics sequencing: Cluster heatmaps displaying differentially abundant metabolites and enrichment analysis heatmap focusing on the oxidative phosphorylation pathway, revealing the impact of INHBA on metabolites. **c** Intracellular and extracellular succinate levels in CRC cells upon INHBA knockdown (*n* = 3). **d** ELISA detection of exogenous succinate supplementation: Using exogenous succinate supplementation, ELISA was used to assess IL-10, Arg-1, TGF-β1, and VEGFA levels in macrophages (*n* = 3). **e** In vivo experiments and exogenous succinate treatment: Subcutaneous tumor implantation experiments in C57BL/6 mice using mouse cells with INHBA knockdown. Starting from day 5, exogenous intraperitoneal injection of succinate was performed every 5 days. On day 20, the mice were euthanized, the tumors were removed and photographed (top), and the tumor weights were statistically analyzed. Tumor growth curves were generated by measuring the tumor volume every 5 days during the growth of the transplanted tumors (bottom) (*n* = 5). **f** Immunohistochemical results: Immunohistochemical results from transplanted tumor samples were used to assess the impact of INHBA on the TME. Scale bar: 50 µm. **g** Flow cytometry results: flow cytometry results from transplanted tumor samples were analyzed for changes in macrophage subpopulations (*n* = 3). For all the statistical plots, the data are depicted as the means ± SD. *P < 0.05, **P < 0.01, ***P < 0.001, ****P < 0.0001

To elucidate how INHBA promotes the M2 polarization of TAMs, we performed RNA sequencing on HCT116 cells overexpressing INHBA and control cells. Prior to analysis, all sequencing data was subjected to rigorous quality assessment and normalization. Principal component analysis (PCA) revealed that samples within every group were tightly clustered and were readily distinguishable, indicating good reproducibility of the samples (Supplementary Fig. 17a). The heatmap of sample correlation also demonstrated good biological reproducibility among the samples (Supplementary Fig. 17b). The differential expression volcano plot depicted 73 up-regulated and 420 down-regulated genes in the INHBA-high samples relative to controls (Supplementary Fig. 17c). Kyoto Encyclopedia of Genes and Genomes (KEGG) enrichment analysis revealed that the “oxidative phosphorylation” pathway was significantly enriched in INHBA-overexpressing samples relative to controls, with a high enrichment factor, a small P value, and numerous DEGs (Fig. 3a).

Additionally, we conducted wide-targeted metabolomics sequencing on HCT116 cells overexpressing INHBA and the control cells. Orthogonal partial least squares discriminant analysis (OPLS-DA) revealed that samples from the two groups formed distinct, well-separated clusters, indicating that the model could effectively distinguish the two groups (Supplementary Fig. 18a). The validation results of the OPLS-DA model revealed that Q² = 0.716 (>0.5), indicating good discriminative ability of the model (Supplementary Fig. 18b). The differentially abundant metabolite volcano plot revealed that, versus controls, INHBA-overexpressing samples yielded 38 up- and 121 down-regulated metabolites (Supplementary Fig. 18c). Differentially abundant metabolite clustering heatmap analysis revealed distinct metabolic expression patterns between the two groups, with similar metabolic expression profiles within each group (Fig. 3b).

On the basis of the transcriptome sequencing results, we conducted enrichment analysis on the differentially expressed metabolites in the oxidative phosphorylation pathway and found that this pathway was enriched with 2 metabolites, namely, nicotinamide adenine dinucleotide (NAD) and succinate (Fig. 3b). The bar chart of the differentially expressed metabolites revealed that NAD and succinate were among the top 20 metabolites with the greatest fold changes (Supplementary Fig. 18d). Furthermore, we measured the levels of NAD⁺ and succinate after INHBA knockdown via ELISA. Upon INHBA knockdown, the intracellular NAD⁺ abundance and the NAD⁺/NADH ratio in CRC cells were significantly decreased (Supplementary Fig. 19a). Additionally, the intracellular accumulation and extracellular release of succinate were also significantly reduced (Fig. 3c). In contrast, the INHBA-overexpressing group presented significantly elevated levels of NAD⁺ and succinate (Supplementary Fig. 19a–c).

Additionally, using the HCT116 cell line, we identified INHBA-interacting proteins through coimmunoprecipitation (co-IP) coupled with liquid chromatography‒mass spectrometry (LC‒MS). We observed that SLC25A10 is a potential interacting protein of INHBA (Supplementary Table 4). It has been reported that succinate generation occurs in the mitochondrial matrix and that SLC25A10 on the mitochondrial membrane can transport succinate, facilitating its rapid equilibrium between the mitochondria and the cytoplasm.^29,44^ Furthermore, using our INHBA-overexpressing cell model for immunoprecipitation (IP) experiments, we detected the expression of SLC25A10 in the immunoprecipitate of INHBA (Supplementary Fig. 20). Conversely, we constructed an HA-SLC25A10-overexpressing CRC cell model and performed IP experiments to detect the expression of INHBA in the immunoprecipitate of SLC25A10 (Supplementary Figs. 21, 22). These results demonstrate the interaction between INHBA and SLC25A10. To determine the subcellular site of the INHBA-SLC25A10 interaction, LoVo cells were transfected with pDsRed2-Mito to label mitochondria and then subjected to immunofluorescence staining and confocal microscopy. The merged images confirmed that INHBA and SLC25A10 colocalized within the mitochondria (Supplementary Fig. 23). Additionally, the literature indicates that tumor-derived succinate functions as a signaling ligand to activate the macrophage surface SUCNR1, thereby promoting their polarization.^32^ Therefore, we speculate that INHBA may skew TAMs toward the M2 phenotype by activating the succinate/SUCNR1 axis.

To verify the above hypothesis, we stimulated THP-1 cells differentiated by PMA with CM from INHBA-knockdown CRC cell lines while simultaneously supplementing them with 0.5 mM exogenous succinate. We then identified IL-10, Arg-1, TGF-β1, and VEGFA expression in macrophages via ELISA. Exogenous succinate partially reversed the decrease in the secretion of IL-10, Arg-1, TGF-β1, and VEGFA by macrophages induced by CM from INHBA-knockdown cells (Fig. 3d). Furthermore, increasing the supplemented succinate concentration to 1 mM or 2 mM markedly intensified this restorative effect (Supplementary Fig. 24a, b). Additionally, the addition of 1 μM NF-56-EJ40 (a human SUCNR1 antagonist) partially reversed the increase in the secretion of IL-10, Arg-1, TGF-β1, and VEGFA by macrophages induced by CM from INHBA-overexpressing cells (Supplementary Fig. 25). We subsequently monitored the polarization phenotype of the THP-1 cells via flow cytometry. Exogenous succinate (0.5 mM) partially reversed the reduction in CD68⁺CD206⁺ and CD68⁺CD163⁺ macrophages proportion induced by CM from INHBA-knockdown cells (Supplementary Fig. 26). The restorative effect further intensified when the succinate concentration was increased to 1 mM or 2 mM (Supplementary Fig. 27). Conversely, the addition of 1 μM NF-56-EJ40 partially reversed the rise in these cells’ percentage induced by CM from INHBA-overexpressing cells (Supplementary Fig. 28). In summary, INHBA facilitates the M2 polarization of TAMs through the succinate/SUCNR1 axis activation in vitro.

Furthermore, we conducted subcutaneous syngeneic tumor transplantation experiments in C57BL/6 mice via mouse cell models with INHBA knockdown. Exogenous supplementation with succinate partially reversed the reduction in tumor volume and weight caused by INHBA knockdown (Fig. 3e). We subsequently detected M2 polarization-related markers of TAMs in these solid tumors and assessed the cell proliferation index via immunohistochemical analysis. Exogenous succinate partially reversed the downregulation of F4/80 and CD206 expression and the decrease in Ki-67 signaling caused by INHBA knockdown (Fig. 3f). Additionally, flow cytometry analysis of syngeneic tumor samples revealed that exogenous succinate partially reversed the decrease in F4/80⁺CD11b⁺CD206⁺ and F4/80⁺CD11b⁺CD163⁺ macrophages proportion caused by INHBA knockdown (Fig. 3g). In summary, these in vivo experimental results further demonstrate that INHBA drives TAMs to polarize toward the M2 phenotype by activating the succinate/SUCNR1 axis, thereby driving the malignant progression of CRC.

### INHBA promotes M2 polarization of TAMs by upregulating SLC25A10 to activate the succinate/SUCNR1 axis

We previously confirmed that INHBA and SLC25A10 interact with each other. To dissect their mutual regulatory mechanism, we conducted the following experiments. First, we constructed cell models with INHBA knockdown and overexpression. Analyses unveiled that INHBA knockdown substantially reduced SLC25A10 protein level but did not significantly alter its mRNA level. Conversely, INHBA overexpression upregulated the protein level of SLC25A10 without affecting its mRNA level (Supplementary Fig. 29a). These findings indicate that INHBA mainly regulates the protein expression of SLC25A10 through posttranscriptional mechanisms. On the basis of the successful construction of the HA-SLC25A10-overexpressing cell model, we subsequently constructed a cell model with SLC25A10 knockdown and confirmed efficient silencing by qPCR and Western blot experiments (Supplementary Fig. 29b). Using this model, we found that SLC25A10 did not significantly alter INHBA mRNA or protein levels (Supplementary Fig. 29c). This result indicates that the regulation of SLC25A10 by INHBA is unidirectional; that is, INHBA positively regulates the protein level of SLC25A10, whereas SLC25A10 does not affect the expression of INHBA. Given that SLC25A10 is a mitochondrial carrier protein, we further used a mitochondrial isolation kit to separate mitochondria and detect the effect of INHBA on the SLC25A10 protein in mitochondria. The findings revealed that INHBA knockdown substantially reduced mitochondrial SLC25A10 protein levels, whereas INHBA overexpression increased its level (Supplementary Fig. 29d). These findings indicate that INHBA not only increases the total protein level of SLC25A10 but also specifically increases its protein level in mitochondria.

**Fig. 4:**
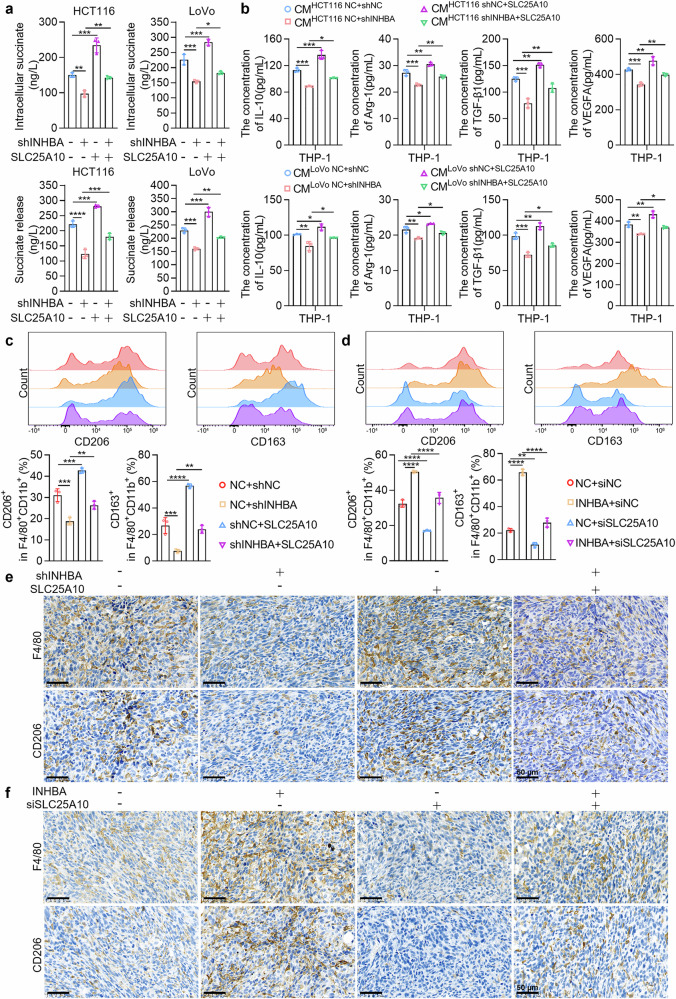
INHBA promotes M2 polarization of TAMs by upregulating SLC25A10 to activate the succinate/SUCNR1 axis. **a** ELISA quantification of intracellular and released succinate in CRC cells upon INHBA silencing and SLC25A10 reconstitution. **b** ELISA quantification of IL-10, Arg-1, TGF-β1, and VEGFA levels in macrophages cultured with CM from human CRC cells with INHBA knockdown and restored SLC25A10 expression. **c** Flow cytometry quantification of F4/80⁺CD11b⁺CD206⁺ and F4/80⁺CD11b⁺CD163⁺ macrophages in mouse syngeneic tumor samples with INHBA knockdown and restored SLC25A10 expression. **d** Flow cytometry quantification of F4/80⁺CD11b⁺CD206⁺ and F4/80⁺CD11b⁺CD163⁺ macrophages in mouse syngeneic tumor samples with INHBA overexpression and SLC25A10 knockdown. **e** IHC for F4/80 and CD206 in mouse syngeneic tumor samples with INHBA knockdown and restored SLC25A10 expression. Scale bar: 50 µm. **f** IHC for F4/80 and CD206 in mouse syngeneic tumor samples with INHBA overexpression and SLC25A10 knockdown. Scale bar: 50 µm. For all the statistical plots, data are means ± SD; *n* = 3 independent experiments. *P < 0.05, **P < 0.01, ***P < 0.001, ****P < 0.0001

To verify whether INHBA drives succinate accumulation and release in CRC cells through SLC25A10, we conducted the following experiments. Restoring SLC25A10 expression could partially reverse the decrease in succinate accumulation and release in CRC cells caused by INHBA knockdown (Fig. 4a). Conversely, knocking down SLC25A10 expression partially reversed the increase in succinate accumulation and release caused by INHBA overexpression (Supplementary Fig. 30). This supports the notion that INHBA regulates succinate metabolism in CRC cells through increased SLC25A10 expression. Furthermore, we investigated whether INHBA drives TAMs toward the M2 phenotype via SLC25A10. ELISA revealed that restoring SLC25A10 partially reversed the reduction in macrophage-derived IL-10, Arg-1, TGF-β1, and VEGFA secretion evoked by CM from INHBA-knockdown cells (Fig. 4b). Conversely, knocking down SLC25A10 expression partially reversed the increase in the secretion of these factors induced by CM from INHBA-overexpressing cells (Supplementary Fig. 31). Additionally, through flow cytometry analysis of macrophage polarization, we observed that restoring SLC25A10 expression could partially reverse the reduced CD68⁺CD206⁺ and CD68⁺CD163⁺ macrophages populations triggered by CM from INHBA-knockdown cells (Supplementary Fig. 32). Conversely, knocking down SLC25A10 expression partially reversed the increase in the proportion of these macrophages induced by CM from INHBA-overexpressing cells (Supplementary Fig. 33). Collectively, these in vitro findings indicate that INHBA drives TAMs toward the M2 phenotype via SLC25A10-mediated succinate/SUCNR1 signaling.

To confirm SLC25A10’s function in CRC, we generated SLC25A10 overexpression and knockdown constructs for use in mouse models and validated their effectiveness by qPCR and Western blot (Supplementary Fig. 34). We subsequently performed subcutaneous syngeneic tumor transplantation experiments in C57BL/6 mice via mouse cell models with INHBA knockdown and restored SLC25A10 expression, as well as models with INHBA overexpression and SLC25A10 knockdown. The dissected tumors were subjected to flow cytometry; restoring SLC25A10 levels partially reversed the decrease in F4/80⁺CD11b⁺CD206⁺ and F4/80⁺CD11b⁺CD163⁺ macrophage populations caused by INHBA knockdown (Fig. 4c). Conversely, knocking down SLC25A10 partially reversed the increase in the proportion of these macrophages caused by INHBA overexpression (Fig. 4d). Additionally, immunohistochemical analysis revealed that restoring SLC25A10 expression could partially reverse the downregulation of F4/80 and CD206 expression caused by INHBA knockdown (Fig. 4e). Conversely, knocking down SLC25A10 partially reversed the upregulation of F4/80 and CD206 expression caused by INHBA overexpression (Fig. 4f). In summary, INHBA promotes the M2 polarization of TAMs through SLC25A10 upregulation to activate the succinate/SUCNR1 axis.

### INHBA inhibits mitochondrial ferroptosis by upregulating SLC25A10 to activate the mtGSH/GPX4 axis

**Fig. 5:**
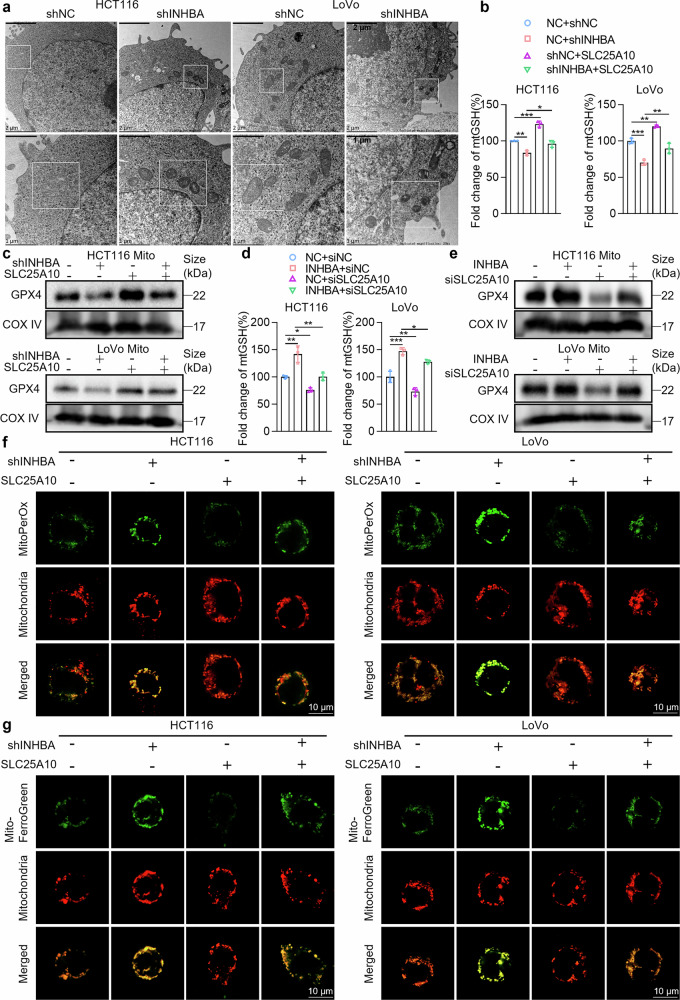
INHBA inhibits mitochondrial ferroptosis by upregulating SLC25A10 to activate the mtGSH/GPX4 axis. **a** TEM of mitochondrial morphology in CRC cells after INHBA silencing. Scale bar: 2 μm (top), 1 μm (bottom). **b** Detection of mtGSH levels (knockdown of INHBA + restoration of SLC25A10): In the cell model with INHBA knockdown and restored SLC25A10 expression, mitochondria were isolated via a mitochondrial separation kit, and mtGSH levels were detected via ELISA. **c** Detection of mitochondrial GPX4 protein (knockdown of INHBA + restoration of SLC25A10): In the cell model with INHBA knockdown and restored SLC25A10 expression, mitochondria were isolated via a mitochondrial separation kit, and mitochondrial GPX4 protein was measured by Western blot. **d** Detection of mtGSH levels (overexpression of INHBA + knockdown of SLC25A10): In the cell model with INHBA overexpression and SLC25A10 knockdown, mitochondria were isolated via a mitochondrial separation kit, and mtGSH levels were detected via ELISA. **e** Detection of mitochondrial GPX4 protein (overexpression of INHBA + knockdown of SLC25A10): In the cell model with INHBA overexpression and SLC25A10 knockdown, mitochondria were isolated via a mitochondrial separation kit, and mitochondrial GPX4 protein was measured by Western blot. **f** Detection of lipid peroxidation levels: In human CRC cells with INHBA knockdown and restored SLC25A10 expression, MitoPerOx was used to measure lipid peroxidation within the mitochondrial inner membrane. **g** Detection of ferrous ion changes: In human CRC cells with INHBA knockdown and restored SLC25A10 expression, changes in ferrous ions in the mitochondria were detected via Mito-FerroGreen. For all the statistical plots, data are means ± SD; *n* = 3 independent experiments. *P < 0.05, **P < 0.01, ***P < 0.001

mtGSH is an important antioxidant that maintains redox homeostasis within mitochondria. The mtGSH/GPX4 axis is pivotal for ferroptosis. However, mitochondria are incapable of synthesizing GSH themselves and instead rely on specific transporters to import GSH from the cytoplasm into the mitochondria. SLC25A10 functions as an essential mtGSH transporter located within the mitochondrial inner membrane.^45^ Given our previous finding that INHBA positively regulates SLC25A10, we hypothesized that INHBA might inhibit mitochondrial ferroptosis in CRC cells by upregulating SLC25A10 to activate the mtGSH/GPX4 axis. First, we isolated mitochondria and examined the regulatory effect of INHBA on mtGSH. INHBA knockdown notably decreased mtGSH levels in tumor cells, whereas its overexpression elevated mtGSH, as shown by ELISA (Supplementary Fig. 35a). Concurrently, we evaluated GPX4 protein expression within mitochondria. CRC cells showed a marked reduction in mitochondrial GPX4 following INHBA knockdown, whereas INHBA overexpression significantly increased mitochondrial GPX4 levels (Supplementary Fig. 35b). We then assessed INHBA’s impact on GPX4 enzymatic activity in CRC cells via a GPX4 activity assay kit. The results revealed that GPX4 activity was markedly decreased upon INHBA knockdown and significantly increased upon INHBA overexpression (Supplementary Fig. 35c). High-performance liquid chromatography (HPLC) quantification of malondialdehyde (MDA), the end product of lipid peroxidation, showed that MDA levels were notably higher in the INHBA-silenced cohort and substantially lower in the INHBA-overexpressing cohort (Supplementary Fig. 35d). Furthermore, we used the fluorescent probes MitoPerOx and Mito-FerroGreen to detect two important indicators of ferroptosis in mitochondria: lipid peroxidation and the accumulation of ferrous ions. The results revealed that the green fluorescence signals reflecting lipid peroxidation and ferrous ion levels rose substantially in the INHBA-knockdown cohort but declined markedly in the INHBA-overexpressing cohort (Supplementary Fig. 35e, f). Furthermore, the increase in green fluorescence caused by INHBA knockdown was partially reversed by the ferroptosis-specific inhibitors liproxstatin-1 or ferrostatin-1, confirming that the fluorescent signals are indeed mediated by ferroptosis-dependent lipid peroxidation and Fe²⁺ accumulation (Supplementary Fig. 36). Additionally, we observed mitochondrial morphology via transmission electron microscopy (TEM) and revealed that the mitochondria in the INHBA-knockdown group presented a reduced volume, increased membrane density, and decreased number of cristae, all of which are typical morphological features of mitochondria during ferroptosis (Fig. 5a). In summary, these findings establish INHBA as a key modulator of mitochondrial ferroptosis. Because oxidative stress is closely linked to DNA damage,^46^ we examined core DNA damage response (DDR) markers γ-H2AX and 53BP1 by immunofluorescence. INHBA silencing markedly increased γ-H2AX/53BP1-colocalized foci, indicating that the loss of INHBA activates the DDR pathway in response to ferroptosis-driven oxidative stress (Supplementary Fig. 37a, b).

To further verify whether INHBA exerts the aforementioned effects through SLC25A10, we constructed cell models with INHBA knockdown and restored SLC25A10 expression, as well as models with INHBA overexpression and SLC25A10 knockdown. ELISA and Western blot revealed that restoring SLC25A10 could partially reverse the decrease in mitochondrial GSH levels and GPX4 expression in CRC cells caused by INHBA knockdown (Fig. 5b, c). Conversely, knocking down SLC25A10 partially reversed the INHBA-overexpression-induced increase in mitochondrial GSH levels and GPX4 expression (Fig. 5d, e). MitoPerOx and MitoFerroGreen fluorescence probe experiments revealed that restoring SLC25A10 expression could partially reverse the increase in lipid peroxidation and ferrous ion levels in CRC cells caused by INHBA knockdown (Fig. 5f, g). Conversely, knocking down SLC25A10 expression partially reversed the reduction in lipid peroxidation and ferrous ion levels caused by INHBA overexpression (Supplementary Fig. 38a, b).

In summary, INHBA promotes the expression of SLC25A10, thereby activating the mtGSH/GPX4 axis and inhibiting mitochondrial ferroptosis in CRC cells. These findings uncover a pivotal pathway through which INHBA controls cell survival and ferroptosis in CRC, providing a novel theoretical basis for subsequent targeted therapies.

### INHBA acts as a scaffold protein to inhibit the TRIM21-mediated ubiquitination and degradation of SLC25A10

**Fig. 6:**
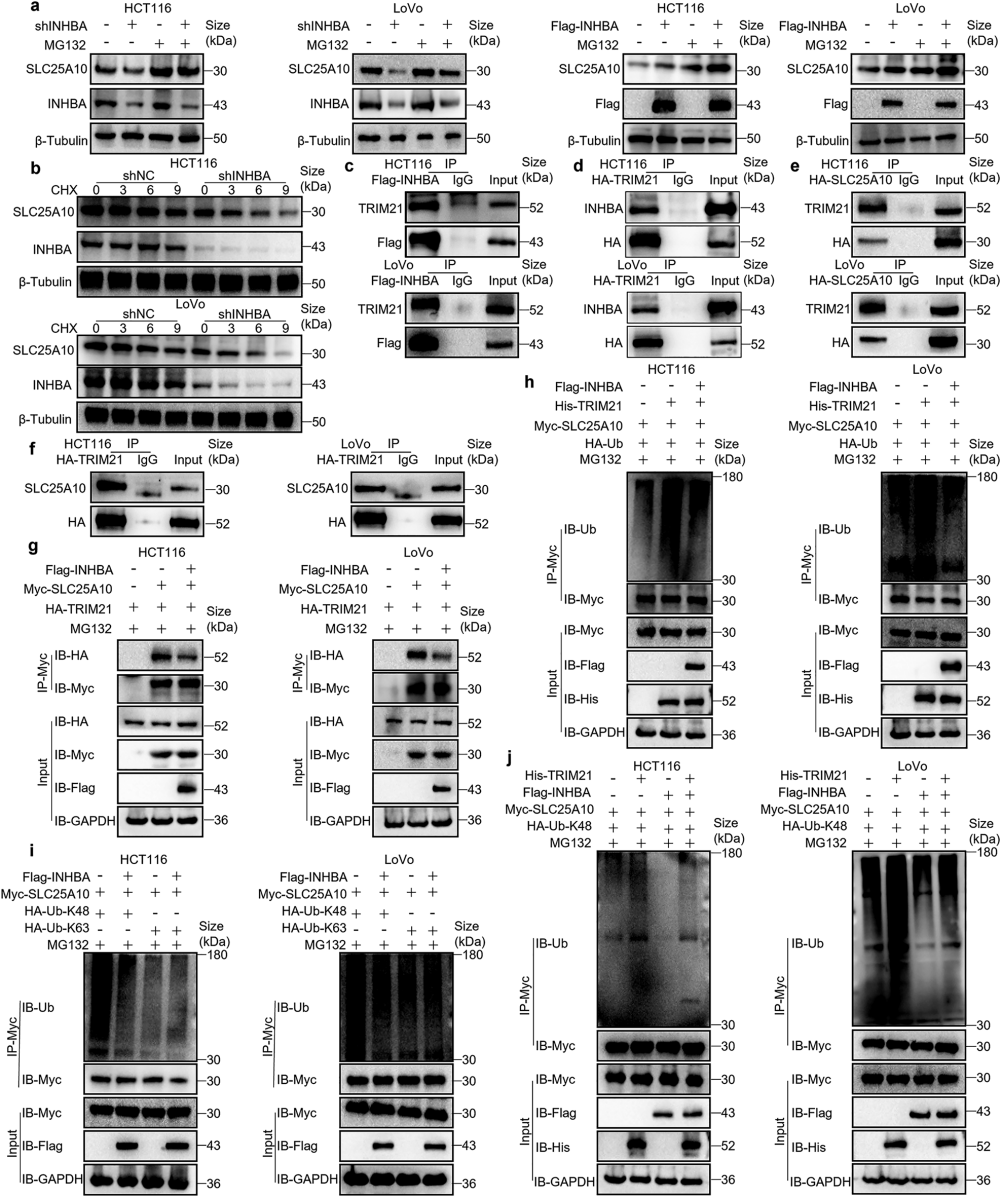
INHBA acts as a scaffold protein to prevent the TRIM21-driven ubiquitination and degradation of SLC25A10. **a** Western blot of SLC25A10 in CRC cells with INHBA silenced or overexpressed after MG132 treatment (20 μM, 4 h). **b** CHX chase (50 μg/ml, TargetMol, USA) of INHBA-knockdown CRC cells; SLC25A10 abundance determined by Western blot at 0, 3, 6, 9 h. **c** IP was performed using anti-Flag to detect INHBA-interacting proteins. **d** IP was performed using anti-HA to identify TRIM21-interacting proteins. **e** IP was were performed using anti-HA to detect SLC25A10-interacting proteins. **f** IP was performed using anti-HA to identify TRIM21-interacting proteins. **g** Enrichment and detection of SLC25A10-interacting proteins: CRC cells were transfected with HA-TRIM21 together with Myc-SLC25A10, Flag-INHBA, or an empty vector. 48 h post-transfection, cells were exposed to MG132 (20 μM, 4 h). Myc-SLC25A10 was immunoprecipitated with anti-Myc beads, and immunoprecipitates were detected via Western blot with anti-HA. **h** Detection of SLC25A10 ubiquitination levels: CRC cells were transfected with Myc-SLC25A10 and HA-Ub together with Flag-INHBA, His-TRIM21, or an empty vector. 48 h post-transfection, cells were exposed to MG132 (20 μM, 4 h). Myc-SLC25A10 was immunoprecipitated with anti-Myc beads, and ubiquitination was detected by anti-Ub Western blot. **i** Detection of SLC25A10 ubiquitination levels (K48 and K63): CRC cells were transfected with Myc-SLC25A10 together with HA-Ub-K63, HA-Ub-K48, Flag-INHBA, or empty vector. 48 hours post-transfection, cells were exposed to MG132 (20 μM, 4 h). Myc-SLC25A10 was immunoprecipitated with anti-Myc beads, and ubiquitination was detected by anti-Ub Western blot. **j** Detection of SLC25A10 ubiquitination levels (K48 linkage): HA-Ub-K48 and Myc-SLC25A10 were co-transfected into CRC cells together with Flag-INHBA, His-TRIM21, or empty vector. After 48 h, cells were exposed to MG132 (20 μM, 4 h). Myc-SLC25A10 was immunoprecipitated with anti-Myc beads, and ubiquitination was detected by anti-Ub Western blot

Previous results confirmed that INHBA increases SLC25A10 protein levels. SLC25A10 is an unstable protein that undergoes ubiquitination and degradation after treatment with ammonium iron (III) citrate (FAC).^45^ Additionally, our mass spectrometry results indicated that TRIM21 is the strongest E3 ubiquitin ligase that interacts with INHBA (Supplementary Table 4). Moreover, a study leveraging the Clinical Proteomic Tumor Analysis Consortium (CPTAC) database and a tissue microarray containing 427 CRC tissues together with their matched adjacent normal mucosa samples demonstrated that TRIM21 is significantly downregulated in tumors; low TRIM21 abundance predicted shorter OS and worse disease-specific survival (DSS).^47^ On the basis of these findings, we postulated that INHBA protects SLC25A10 from ubiquitin–proteasome–mediated degradation. To test this hypothesis, we used INHBA-overexpressing and INHBA-knockdown cell models, exposed them to MG132 or cycloheximide (CHX), and monitored SLC25A10 stability by Western blot. Upon proteasome inhibition with MG132, SLC25A10 abundance was restored in INHBA-knockdown cells and further elevated in INHBA-overexpressing cells (Fig. 6a). After CHX treatment, the Western blot revealed accelerated SLC25A10 degradation and a significantly shorter half-life in INHBA-knockdown cells relative to controls (Fig. 6b). In summary, INHBA protect the SLC25A10 protein from ubiquitin–proteasome–mediated degradation.

To verify whether INHBA stabilizes SLC25A10 via TRIM21, we first overexpressed Flag-INHBA in CRC cells and conducted IP experiments. TRIM21 was present in INHBA immunoprecipitates (Fig. 6c), indicating an interaction between INHBA and TRIM21. Furthermore, HA-TRIM21-overexpressing CRC cells were generated (Supplementary Fig. 39) and INHBA in TRIM21 immunoprecipitates was detected by IP (Fig. 6d). These results demonstrated that INHBA binds TRIM21. Given our previous demonstration of an INHBA–SLC25A10 interaction, we hypothesized that TRIM21 might also interact with SLC25A10. To test this, we separately overexpressed HA-SLC25A10 and HA-TRIM21 in CRC cells and confirmed their interaction by IP (Fig. 6e, f). To further clarify the regulatory relationships among these three proteins, Western blot analysis showed that INHBA and TRIM21 do not reciprocally regulate each other’s protein levels (Supplementary Fig. 40a, b). However, TRIM21 overexpression markedly attenuated total and mitochondrial SLC25A10, whereas its knockdown upregulated them (Supplementary Fig. 40c–g). To explore how TRIM21 regulates SLC25A10, we constructed a CRC cell model overexpressing His-TRIM21 (Supplementary Fig. 40h). We subsequently cotransfected CRC cells with His-NC or His-TRIM21 and an HA-Ub (ubiquitin) expression vector. After 48 h, cells underwent MG132 treatment for 4 hours, and were subjected to anti-SLC25A10 IP. TRIM21 overexpression markedly boosted ubiquitinated SLC25A10 (Supplementary Fig. 41a). In contrast, CRC cells underwent cotransfection with shNC or shTRIM21 together with an HA-Ub expression vector. Following a 48-h period, cells received 4-h MG132 exposure, and subjected to anti-SLC25A10 IP. TRIM21 knockdown markedly decreased ubiquitinated SLC25A10 (Supplementary Fig. 41b). These findings indicate that TRIM21 ubiquitinates and directs SLC25A10 to proteasomal degradation.

To further clarify whether INHBA acts as a scaffold protein to prevent TRIM21-induced ubiquitination of SLC25A10, we generated Myc–SLC25A10-overexpressing human CRC cells (Supplementary Fig. 42). IP experiments demonstrated that INHBA overexpression markedly weakens TRIM21–SLC25A10 interaction (Fig. 6g) and inhibited TRIM21-driven SLC25A10 ubiquitination (Fig. 6h), indicating that INHBA disrupts TRIM21–SLC25A10 association to block SLC25A10 ubiquitination and degradation. K48 and K63 ubiquitin chains constitute the primary linkage forms.^48^ To further determine the ubiquitin linkage that INHBA blocks to stabilize SLC25A10, we performed IP experiments. Upon equal capture of Myc–SLC25A10, INHBA inhibited mainly the K48-linked polyubiquitination rather than the K63-linked form (Fig. 6i). Further IP experiments confirmed that INHBA, acting as a scaffold protein, inhibits TRIM21-mediated K48-linked ubiquitination of the SLC25A10 protein (Fig. 6j). Additionally, we overexpressed TRIM21 and INHBA simultaneously in CRC cells and extracted mitochondria via a mitochondrial isolation kit. Western blot analysis showed that INHBA substantially suppressed TRIM21-mediated ubiquitination and degradation of the mitochondrial SLC25A10 protein (Supplementary Fig. 43). Together, our data show that INHBA acts as a scaffold protein to stabilize the SLC25A10 by inhibiting TRIM21-directed K48-linked ubiquitylation.

### INHBA promotes the malignant progression of colorectal cancer by regulating the mitochondrial protein SLC25A10

**Fig. 7:**
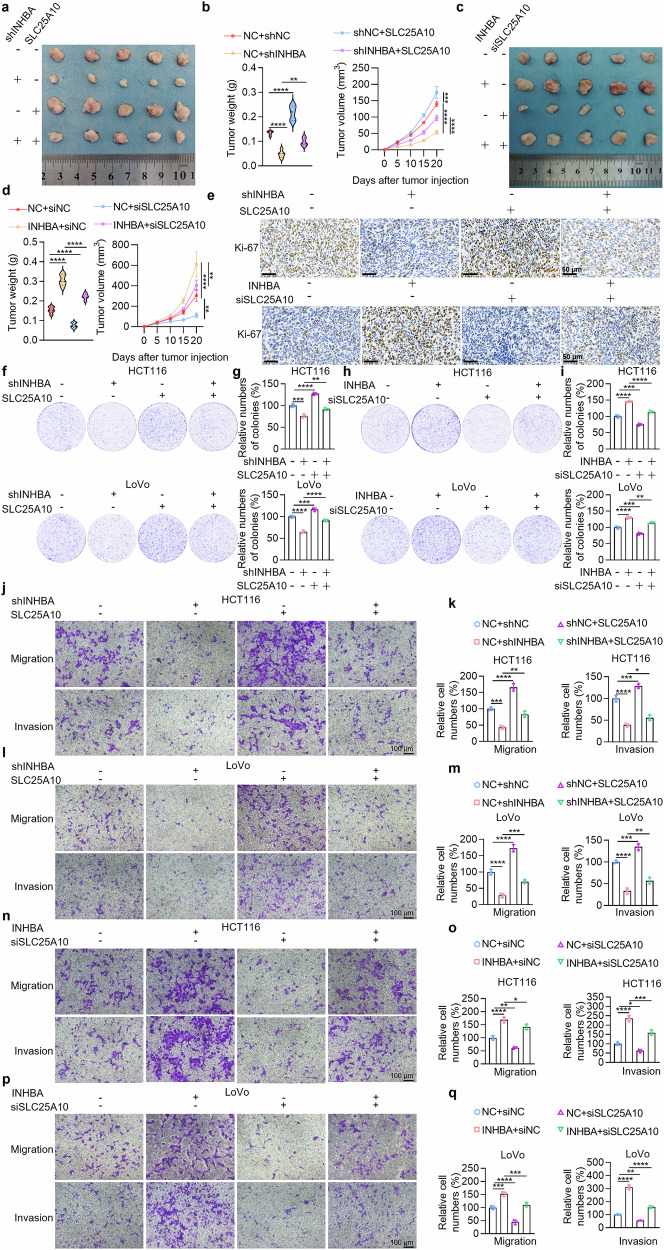
INHBA drives CRC aggressiveness via activation of the mitochondrial SLC25A10. **a** In vivo experiments (knockdown of INHBA + restoration of SLC25A10): Subcutaneous tumor implantation experiments in C57BL/6 mice using mouse cell models with INHBA knockdown and restored SLC25A10 expression. Photographs of tumors removed from euthanized mice are shown. **b** Tumor weight and growth curve (knockdown of INHBA + restoration of SLC25A10): Statistical chart of tumor weight and tumor growth curves obtained by measuring tumor volume every 5 days during the growth of transplanted tumors (*n* = 5). **c** In vivo experiments (overexpression of INHBA + knockdown of SLC25A10): Subcutaneous tumor implantation experiments in C57BL/6 mice using mouse cell models with INHBA overexpression and SLC25A10 knockdown. Photographs of tumors removed from euthanized mice are shown. **d** Tumor weight and growth curve (overexpression of INHBA + knockdown of SLC25A10): Statistical chart of tumor weight and tumor growth curves obtained by measuring tumor volume every 5 days during the growth of transplanted tumors (*n* = 5). **e** Immunohistochemical experiments: Detection of the effects of INHBA knockdown or overexpression with restored SLC25A10 expression on Ki-67 in mouse syngeneic tumors. Scale bar: 50 µm. **f** Colony formation assay (knockdown of INHBA + restoration of SLC25A10): Detection of the effects of INHBA knockdown with restored SLC25A10 expression on cell proliferation. **g** Colony number quantification (knockdown of INHBA + restoration of SLC25A10): *n* = 3. **h** Colony formation assay (overexpression of INHBA + knockdown of SLC25A10): Detection of the effects of INHBA overexpression with SLC25A10 knockdown on cell proliferation. **i** Colony number quantification (overexpression of INHBA + knockdown of SLC25A10): *n* = 3. **j** Cell migration and invasion assays (HCT116, knockdown of INHBA + restoration of SLC25A10): Detection of the effects of INHBA knockdown with restored SLC25A10 expression on the migration and invasion of HCT116 cells. Scale bar: 100 µm. **k** Quantification of migrated/invaded cells (HCT116, knockdown of INHBA + restoration of SLC25A10): *n* = 3. **l** Cell migration and invasion assays (LoVo, knockdown of INHBA + restoration of SLC25A10): Detection of the effects of INHBA knockdown with restored SLC25A10 expression on cell migration and invasion in LoVo cells. Scale bar: 100 µm. **m** Quantification of migrated/invaded cells (LoVo, knockdown of INHBA + restoration of SLC25A10): *n* = 3. **n** Cell migration and invasion assays (HCT116, overexpression of INHBA + knockdown of SLC25A10): Detection of the effects of INHBA overexpression with SLC25A10 knockdown on the migration and invasion of HCT116 cells. Scale bar: 100 µm. **o** Quantification of migrated/invaded cells (HCT116, overexpression of INHBA + knockdown of SLC25A10): *n* = 3. **p** Cell migration and invasion assays (LoVo, overexpression of INHBA + knockdown of SLC25A10): Detection of the effects of INHBA overexpression with SLC25A10 knockdown on cell migration and invasion in LoVo cells. Scale bar: 100 µm. **q** Quantification of migrated/invaded cells (LoVo, overexpression of INHBA + knockdown of SLC25A10): *n* = 3. For all the statistical plots, data are means ± SD. *P < 0.05, **P < 0.01, ***P < 0.001, ****P < 0.0001

To further verify the in vivo role of SLC25A10, we subcutaneously transplanted syngeneic tumor into C57BL/6 mice. Restoring SLC25A10 expression partly reversed the reduced tumor volume and weight caused by INHBA knockdown (Fig. 7a, b), whereas silencing SLC25A10 partly attenuated the tumor volume and weight enhancements due to INHBA upregulation (Fig. 7c, d). Thus, SLC25A10 is required for INHBA-driven CRC progression in vivo. Furthermore, IHC of dissected tumors demonstrated that SLC25A10 expression restoration rescued the diminished Ki-67 signal caused by INHBA knockdown (Fig. 7e). Conversely, knocking down SLC25A10 partially reversed the increase in the Ki-67 signal caused by INHBA overexpression (Fig. 7e). Next, IHC of 30 CRC tissue samples and matched adjacent normal mucosa revealed high SLC25A10 expression in cancerous tissues. Consistently, TCGA analysis indicated high SLC25A10 expression strongly correlated with shorter OS in COAD patients (Supplementary Fig. 44).

Moreover, colony-forming data revealed that restoring SLC25A10 could partially reverse the reduced CRC cell proliferation capacity caused by INHBA knockdown (Fig. 7f, g). Conversely, knocking down SLC25A10 partially reversed the increased CRC cell proliferation capacity caused by INHBA overexpression (Fig. 7h, i). Thus, SLC25A10 mediates the pro-proliferative effect of INHBA in CRC. Furthermore, cell migration and invasion assays demonstrated that restoring SLC25A10 could partially reverse the reduced CRC cell migration and invasion resulting from INHBA knockdown (Fig. 7j–m). Conversely, knocking down SLC25A10 partially reversed the increased CRC cell migration and invasion caused by INHBA overexpression (Fig. 7n–q). In summary, INHBA promotes CRC malignancy by stabilizing the mitochondrial protein SLC25A10.

## Discussion

In this work, we integrated multi-omics with in vitro and in vivo models to delineate INHBA function in CRC. INHBA is markedly up-regulated, predicts adverse prognosis, and fosters malignancy by skewing TAMs toward M2 and inhibiting mitochondrial ferroptosis. Mechanistically, INHBA stabilizes the mitochondrial protein SLC25A10, activating the succinate/SUCNR1 and mtGSH/GPX4 axes, thus defining a therapeutically targetable driver of CRC progression.

Previous studies have shown INHBA to be markedly up-regulated in colorectal cancer, cervical cancer, breast cancer, gastric cancer, lung adenocarcinoma and other cancers, correlating strongly with aggressive disease and adverse survival.^49–53^ Our data corroborate these findings, and extend them by dissecting how INHBA remodels the TME and restrains ferroptosis in CRC, mechanisms that had remained undefined. Importantly, we show that INHBA maintains mitochondrial SLC25A10 stability, thereby activating the succinate/SUCNR1 axis and the mtGSH/GPX4 axis to promote tumor malignancy. These findings provide a more comprehensive perspective on INHBA’s function in CRC and offer a novel theoretical basis for future targeted therapies.

This study is novel in revealing the molecular mechanisms by which INHBA stabilizes SLC25A10 and activates the succinate/SUCNR1 axis and the mtGSH/GPX4 axis, thereby regulating TAM M2 polarization and inhibiting mitochondrial ferroptosis, to advance CRC malignancy. Additionally, this study revealed a new mechanism by which INHBA acts as a scaffold protein to inhibit TRIM21-mediated ubiquitination and degradation of SLC25A10. These findings establish a mechanistic framework for INHBA-driven colorectal tumorigenesis and simultaneously offer new directions for devising focused therapeutic methods grounded in INHBA and its regulatory pathways.

Despite the pivotal function of INHBA in CRC and the mechanistic insights uncovered herein, several limitations remain. First, this research relies chiefly on cellular assays in vitro and animal models. Although these experiments have provided important clues for understanding the mechanisms of INHBA in CRC, they require validation in large-scale clinical samples. The human TME arises through intricate crosstalk among malignant cells, immune cells, endothelial cells, fibroblasts and additional stromal elements.^54^ The mouse models used here, however, harbor a simpler immune system that lacks human-specific leukocyte subsets; consequently, they can only partially reconstitute this complex ecosystem. Moreover, human CRC is genetically and molecularly highly heterogeneous,^55^ whereas conventional mouse strains carry a uniform genetic background, limiting their ability to mirror such diversity. Additionally, human colitis-associated colorectal cancer (CAC) develops against a background of inflammatory bowel disease (IBD),^56^ where chronic inflammation reshapes the immune milieu and directly drives carcinogenesis. In contrast, subcutaneous transplant tumors in mice are devoid of the gut-specific inflammatory context; their immune landscape also differs markedly from that of primary human lesions, further weakening the models’ fidelity to the authentic TME. Therefore, INHBA’s precise function and pathways in CRC patients still need to be confirmed through further clinical studies. Future research should include the analysis of an extensive clinical cohort to validate the potential of INHBA as a biomarker and to elucidate its expression differences across different clinical stages. Second, this study focused on the impact of INHBA on the polarization of TAMs and revealed its mechanisms by skewing TAMs toward the M2 phenotype to fuel tumor progression. However, the complexity of the immune microenvironment suggests that INHBA could also exert essential roles in other immune cells, including B cells, T cells, and dendritic cells, which critically shape the anti-tumor immune response. Therefore, future research should further explore the regulatory effects of INHBA on these immune cells to comprehensively understand its functions in the TME. Finally, this research mainly employed overexpression and knockdown approaches; CRISPR knock-in or inducible systems to evaluate the enduring physiological impacts of the INHBA/SLC25A10 axis have not yet been implemented and will be addressed in future work.

Future research will focus on the following aspects. First, a substantial body of literature has demonstrated that INHBA exerts tumor-promoting effects on multiple cancers by triggering TGF-β pathway activation.^17–19^ Several TGF-β receptor kinase inhibitors, such as galunisertib, have already entered clinical trials.^57^ However, TGF-β typically exerts tumor-suppressive activity during early oncogenesis but converts to a progression-driving cue once the lesion advances.^58^ This dual role makes global inhibition of TGF-β signaling potentially hazardous. Consequently, developing specific INHBA inhibitors could overcome the challenges posed by the pleiotropic effects of TGF-β, offering a more precise therapeutic strategy. Moreover, studies have shown that tumor-derived INHBA can dampen the response to immune checkpoint blockade by suppressing interferon-γ (IFN-γ) pathway, reducing programmed death-ligand 1 (PD-L1), and impairing T-cell chemokines secretion like C-X-C motif chemokine ligand 9/10 (CXCL9/10). Combining garetosmab, an activin-A (INHBA homodimer)-neutralizing antibody, with atezolizumab, an anti-PD-L1 antibody, yielded enhanced antitumor efficacy compared with either agent alone.^11^ Thus, small-molecule inhibitors specifically targeting INHBA hold promise to transform immunologically “cold” tumors into “hot” ones and for enhancing immune checkpoint inhibitors’ efficacy, offering a broad and attractive therapeutic perspective. Through these studies, we seek novel therapeutic approaches for CRC to advance targeted therapy progress.

## Materials and methods

### Ethics declarations

The animal protocols received authorization from Hunan Cancer Hospital’s Institutional Animal Care and Use Committees (Permit number: KNZY-202411). Tissue microarray acquisition received Shanghai Outdo Biotech Company Ethics Committee approval (Permit numbers: SHYJS-CP-101005 and YB M-05-02). The study protocol for validating SLC25A10 levels in human colon cancer specimens passed review and received approval from Hunan Cancer Hospital’s Ethics Committee (Permit number: 143).

### Cell strains and maintenance

HCT116, LoVo and MC38 cell lines were provided by the Cell Center, Central South University. HCT116 and LoVo were maintained in DMEM containing 10% fetal bovine serum (FBS) from Procell (Wuhan, China) and 1% penicillin/streptomycin (NCM Biotech, Suzhou, China). MC38 was grown in RPMI-1640 medium (Procell) containing identical supplements. All cells were grown at 37 °C in a 5% CO₂ humidified atmosphere.

### Lentiviral vector transfection

The lentiviral vectors used for the overexpression and knockdown of human and mouse INHBA were sourced from Genechem (Shanghai, China); empty vector served as control. Human and mouse CRC cells were transfected with these lentiviral vectors. Post-transfection, cells received 2 μg/ml puromycin (BasalMedia, Shanghai, China) in the culture medium to select and enrich the transfected cells with antibiotic resistance. INHBA overexpression and silencing efficiency were subsequently assessed via qPCR and Western blot.

### Plasmid constructs, siRNA sequences, and cellular transfection

The plasmids used in this study included human INHBA pcDNA3.1-Flag-C, human INHBA sh1 + 2 pLKO.1, human SLC25A10 pcDNA3.1-HA-C, human SLC25A10 pcDNA3.1-MYC-C, human TRIM21 pcDNA3.1-HA-C, human TRIM21 pcDNA3.1-His-C, human shTRIM21 pLKO.1, and mouse SLC25A10 pcDNA3.1-HA-C, all of which are ampicillin resistant. Additionally, the plasmid pDsRed2-Mito is kanamycin resistant. UNIBIO (Changsha, China) supplied these plasmids. Professor Ming Zhou of Central South University kindly supplied the plasmids pCMV-HA-Ub and its K48 and K63 variants.

Morzan Biotech (Shanghai, China) synthesized the siRNAs for this investigation with these sequences: human si-SLC25A10, sense 5’-GGAUGCAGAACGACGUGAA-3’, and antisense 5’-UUCACGUCGUUCUGCAUCC-3’. Mouse si-SLC25A10, sense 5’-GUACCUGAGUGACAACAUATT-3’, antisense 5’-UAUGUUGUCACUCAGGUACTT-3’.

Cell transfections utilizing the jetPRIME® transfection reagent (Polyplus®, France) were executed adhering to the supplier’s guidelines.

### RNA isolation, reverse transcription, and RT–qPCR

Using the Total RNA Rapid Extraction Kit for Cells/Tissues (NCM Biotech), we isolated total cellular RNA following the manufacturer’s protocol. Total RNA was subsequently converted to cDNA via All-in-One Script RT premix (with dsDNase) (KERMEY, Zhengzhou, China). RT–qPCR was then performed with 2×SYBR Green qPCR Premix (KERMEY). The sequences of primers employed are listed in Supplementary Table 5.

### Western blot

Whole-cell lysates were prepared with RIPA buffer containing protease-inhibitor cocktail (NCM Biotech) following the supplier’s protocol. Mitochondria were isolated and lysed with the Cell Mitochondria Isolation Kit (Beyotime Biotechnology, Shanghai, China). BCA kit (Abbkine Scientific, Wuhan, China) was used to measure protein content, using bovine serum albumin (BSA) for the standard curve. Using 10% SDS‒PAGE gels (NCM Biotech), the proteins underwent electrophoretic separation before being transferred onto PVDF membranes supplied by Millipore (USA). After blocking with 5% skim milk (Yili, China), membranes were probed overnight at 4 °C with primary antibodies in blocking buffer. The primary antibodies used are detailed in Supplementary Table 6. After being washed three times with PBST for 15 min each, the membranes were subjected to 1-h incubation at 37 °C with HRP-linked secondary antibodies, and then were washed again three times with PBST for 15 min each. Proteins were detected via an enhanced chemiluminescence (ECL) solution (NCM Biotech) according to the manufacturer’s protocol.

### Coimmunoprecipitation

For co-immunoprecipitation, 30 μL protein A/G magnetic beads (Biolinkedin®, Shanghai, China) were pre-conjugated with antibodies by incubation at room temperature for 2 h. Alternatively, specific antigen-coated magnetic beads, including anti-DYKDDDDK (Flag), anti-Myc, and anti-HA magnetic beads (Biolinkedin®, Shanghai, China), were directly used for coimmunoprecipitation without prior antibody conjugation. These beads were selected on the basis of the epitope tags present in the target proteins. Cells were lysed in Western and IP lysates (NCM Biotech) plus protease inhibitors. Upon centrifugation (12,000 × *g*, 4 °C, 15 min), the supernatant was rocked with either antibody-bound or the specific antigen-coated magnetic beads overnight at 4 °C. The immune-complex-laden beads were then meticulously rinsed six times with pre-cooled lysis buffer, eluted by boiling, and analyzed by Western blot. See Supplementary Table 6 for antibody details.

### Ubiquitination assay

Cells seeded in 10-cm dishes were subjected to 48 h transfection, treated with 20 μM MG132 (MCE, USA) for 4 h, and lysed. Anti-Myc magnetic beads were used for IP, and ubiquitination was evaluated via Western blot with an anti-ubiquitin antibody.

### Immunofluorescence assays

For cellular immunofluorescence staining, we employed a multiplex fluorescence staining kit from AiFang Biological (Hunan, China). The samples underwent initial fixation using a 4% paraformaldehyde solution (Biosharp®, Beijing, China) for 15 minutes, followed by permeabilization with 0.1% Triton X-100 (Beyotime Biotechnology) for 20 min to enhance antibody penetration. Following 30 min of blocking with 5% BSA (Bovogen, Australia) to minimize nonspecific binding, cells underwent primary antibody incubation overnight at 4 °C.

The next day, the secondary antibody was applied under ambient conditions for 30 min, followed by a 10-min incubation with the fluorescent dye. After two 10-min washes at 37 °C with elution buffer, samples were subjected to a second round of blocking, primary and secondary antibody incubation, and fluorescent dye staining. To visualize the cell nuclei, we treated the cells with DAPI (Beyotime Biotechnology) for 10 min. Finally, photographic documentation was acquired from no fewer than three randomly selected regions utilizing either an Olympus fluorescence microscope (Japan) or a Leica laser confocal microscope (Germany).

For tissue-section immunofluorescence staining, the sections underwent xylene dewaxing, followed by gradual rehydration using a descending ethanol gradient, and underwent antigen retrieval with AR6 buffer (Akoya Biosciences, USA) in a microwave. Following 10-min incubation with 3% H₂O₂ to inhibit endogenous peroxidase, multiplex immunofluorescence was carried out in sequential cycles: 1% BSA blocking, primary antibody, HRP-conjugated secondary antibody (Akoya Biosciences), and Opal fluorophore (1:100) with tyramide signal amplification. Antibody complexes were stripped by heat-mediated retrieval between rounds. Following the final cycle, the slides underwent DAPI spectral counterstaining (Akoya Biosciences) and mounting in Abcam antifade medium (UK). Images were acquired at ×200 magnification via a PANNORAMIC SCAN II slide scanner (3Dhistech, Hungary). Supplementary Table 7 presents the specifics of the utilized antibodies.

### Colony formation assay

Post-transfection, the cell suspension was diluted to 1000 cells/mL within the culture medium before being transferred to 12-well plates. Cells were then cultured continuously for 10–14 days or until well-defined colonies emerged, then stained with crystal violet (Solarbio®, Beijing, China) for visualization.

### EdU cell proliferation assay

CRC cells with stable INHBA overexpression or INHBA knockdown were cultivated in 12-well dishes prelined with coverslips. After culturing for 12 hours, a 2×EdU working solution (final concentration of 20 μM) was prepared and prewarmed at 37 °C. This prewarmed solution was added at equal volume to yield 1× EdU (10 μM) for 2 h. Medium was then aspirated, cells were fixed with 4% paraformaldehyde for 15 min, washed three times (5 min each) with wash buffer, and permeabilized for 10–15 min at ambient temperature. Once permeabilized, they were subjected to two additional washes (5 min each), incubated with Click reaction mix for a half-hour in the dark, and washed three times (3–5 min each) with wash buffer. Finally, the cell nuclei were dyed with Hoechst 33342 and imaged on an Olympus fluorescence microscope (Japan).

### Cell migration and invasion assays

To evaluate migration and invasion, Transwell chambers (Millipore) were employed. The lower compartment contained DMEM with 20% FBS. 2% FBS cell suspensions were plated in the upper chamber (with or without Matrigel, Corning, USA). Following a 48-h incubation period at 37 °C, non-migrated/non-invaded cells on the upper surface were removed with a cotton swab. Chambers were fixed with 4% paraformaldehyde (30 min) and crystal-violet-stained (1%, 10 min, Solarbio®). Finally, cells on the underside were imaged and quantified under an inverted phase-contrast microscope in three randomly picked fields.

### Preparation of conditioned medium and coculture of CRC cells with THP-1 cells

To obtain CM from CRC cells, supernatants were collected 48 h post-transfection, centrifuged (3000 rpm, 10 min), and filtered (0.22 μm, Millipore). CM was either used immediately to coculture with PMA-differentiated THP-1 cells (100 ng/ml, 24 h, MedChemExpress, USA) or stored at −80 °C for later use.

In CRC cells–THP-1 transwell cocultures, CRC cells were transfected for 48 h and seeded into 24 mm, 0.4 μm pore inserts (37006, SPL Life Sciences, South Korea). Inserts were placed in 6-well plates containing PMA-induced THP-1 cells (100 ng/ml, 24 h) and cocultured for a further 24 h.

### Enzyme-linked immunosorbent assay

To assess IL-10, Arg-1, TGF-β1, and VEGFA expression in macrophages, CM was cocultured with PMA-induced (100 ng/ml, 24 h) THP-1 cells for an additional 24 h. Alternatively, we adopted CRC cell–THP-1 transwell coculture experiments and maintained the coculture for an additional 24 h. We subsequently measured the levels of these cytokines via ELISA kits (ELK Biotechnology, Wuhan, China).

To measure the intracellular and extracellular succinate levels, as well as the intracellular NAD⁺ and NAD⁺/NADH ratios, in CRC cells, we used succinate ELISA kits (MEI MIAN, Jiangsu, China) and NAD⁺/NADH detection kits (Beyotime Biotechnology) 48 h after cell transfection.

To determine intracellular GPX4 activity in CRC cells, samples were collected 48 h post-transfection and analyzed via the GPX4 Activity Assay Kit (Elabscience, Wuhan, China) adhering to the kit’s protocol.

To determine the GSH content in the mitochondria of CRC cells, we first isolated the mitochondria via a mitochondrial isolation kit (Beyotime Biotechnology). We then measured the GSH content via a reduced GSH colorimetric assay kit (Elabscience).

The levels of IL-10, Arg-1, TGF-β1, VEGFA, succinate, NAD⁺, and GSH, as well as GPX4 enzyme activity, were measured via a Merck microplate reader (Germany).

### Wide-targeted metabolomic sequencing

HCT116 cells in 150 mm dishes were transfected for 48 h with either negative-control or INHBA-overexpression vectors, snap-frozen on dry ice, and sent to MetWare (Wuhan, China) for further examination.

### Liquid chromatography with tandem mass spectrometry

Gel bands were destained, reduced/alkylated, and digested with trypsin (37 °C, overnight). Peptides were dried, re-suspended in LC buffer, and analyzed on an LTQ-Orbitrap Velos (Thermo Fisher, USA) coupled to a Dionex RSLC nano-LC. Proteins were identified with Proteome Discoverer 1.4 (Thermo Fisher) against UniProtKB/Swiss-Prot (10 ppm precursor, 0.8 Da fragment tolerance); peptides with false discovery rate (FDR) < 1% were retained.

### Measurements of mitochondrial iron using Mito-FerroGreen

Mito-FerroGreen stands out as a specialized probe designed to detect ferrous ions within mitochondria. CRC cells transfected for 48 h were placed in confocal dishes and allowed to incubate for another 12 h. Where indicated, ferroptosis inhibitors were added at seeding: liproxstatin-1 (MCE) at 1 μM (HCT116) or 10 μM (LoVo); ferrostatin-1 (MCE) at 0.1 μM (HCT116) or 1 μM (LoVo). Cells were then cultured for 12 h. Following incubation in a Mito-FerroGreen solution (5 μM, MCE) at 37 °C for 30 min, the cells underwent a PBS wash and imaged on a Leica laser confocal microscope.

### Measurements of mitochondrial lipid peroxidation via MitoPerOx

MitoPerOx is a mitochondrion-targeted fluorescent probe for detecting lipid peroxidation. CRC cells transfected for 48 h were placed in confocal dishes and allowed to incubate for another 12 h. Where indicated, ferroptosis inhibitors were added at seeding: liproxstatin-1 at 1 μM (HCT116) or 10 μM (LoVo); ferrostatin-1 at 0.1 μM (HCT116) or 1 μM (LoVo). Cells were then cultured for 12 h. Following incubation in a MitoPerOx solution (100 nM, MCE) at 37 °C for 30 min, the cells underwent a PBS wash and imaged on a Leica laser confocal microscope.

### Immunohistochemistry

For IHC, 4 μm paraffin sections were deparaffinized, rehydrated and subjected to heat-induced antigen retrieval. The peroxidase antiperoxidase (PAP) method was employed for the immunohistochemical staining process. Sections were blocked, then incubated overnight at 4 °C with primary antibodies (Supplementary Table 8). HRP-linked secondary antibodies were applied at ambient temperature for 30 min. Immunoreactivity was visualized with 3,3’-diaminobenzidine (DAB), counterstained with hematoxylin (3 min).

### Flow cytometry

To assess INHBA-driven M2 macrophage polarization, we cocultured CM with PMA-differentiated (100 ng/ml, 24 h) THP-1 cells for an additional 24 h. Alternatively, we adopted CRC cell–THP-1 transwell coculture experiments and maintained the coculture for another 24 h. Following digestion with 0.25% trypsin solution without EDTA (BasalMedia), cells were prepared as single-cell suspensions. Live cells were first labeled via the Zombie Aqua™ Fixable Viability Kit (423102, BioLegend), followed by stained with flow cytometry antibodies, which included antibodies against CD68, CD163, and CD206. Supplementary Table 9 lists the antibodies utilized in flow cytometry. The specific procedure was as follows: After surface staining with CD206 and CD163, cellular samples underwent fixation and permeabilization using the Cytofix/Cytoperm™ Fixation/Permeabilization Kit (554714, BD Biosciences) adhering to the manufacturer’s protocol. Following intracellular CD68 staining, M2-type macrophages were gated as CD68⁺CD206⁺ or CD68⁺CD163⁺ populations.

To quantify phagocytosis after INHBA-driven M2 polarization, THP-1 monocytes were differentiated with PMA (100 ng/ml, 24 h). Macrophages were incubated for 24 h with CM from CRC cells engineered to overexpress or knock down INHBA. After 2 h of serum starvation, the macrophages were cocultured with untransfected CRC cells at a 5:1 effector-target ratio for 4 h. Prior to coculture, the tumor cells were labeled with CFDA-SE (Beyotime Biotechnology), and the macrophages were labeled with PKH26 (Solarbio®). Finally, the percentage of PKH26⁺CFDA-SE⁺ macrophages was determined via flow cytometry to assess phagocytic activity.

For flow cytometry of tissue samples, we minced and homogenized the tumor tissues and then filtered them via a 70 μm strainer for single-cell isolation. First, we blocked the samples with purified rat anti-mouse CD16/CD32 (553141, BD Biosciences). Next, Zombie Aqua™ Fixable Viability Kit (423102, BioLegend) incubated with cells for 15 min at ambient conditions to label live cells. Following PBS washing, the cells underwent centrifugation at 400 × *g* for 5 min to eliminate free dye. The samples were subsequently stained with flow cytometry antibodies, which included antibodies against CD45, F4/80, CD11b, CD163, and CD206 (Supplementary Table 9). Following a 30-minute antibody incubation at ambient temperature, the cells underwent washing in PBS and were subjected to centrifugation at 400 × *g* for 5 min to eliminate free antibodies. Erythrocytes were depleted by treating the single-cell suspension with red blood cell lysis buffer. After washing and centrifugation, samples were acquired on a BD FACSAria III flow cytometer with data analysis performed through FlowJo software (Tree Star, USA).

### High-performance liquid chromatography

To assess MDA levels, CRC cells underwent transfection with INHBA overexpression or knockdown vectors. Post-48-h transfection, the samples were harvested, transported on dry ice to Shanghai Best Choice Biotechnology Co., Ltd., and analyzed on an Agilent 1200 HPLC system equipped with a VWD detector set at 310 nm.

### Clinical specimens

Following ethics committee approval from Shanghai Outdo Biotech Co., Ltd, a tissue microarray containing 96 colon cancer specimens (diagnosed 2004–2009) was constructed. Clinical and pathological data were retrieved from surgical pathology and medical records. Histological typing adhered to the WHO’s classification standards, and staging was referenced from the latest edition of the American Joint Committee on Cancer (AJCC) guidelines, specifically the 8th version.

Two independent pathologists, blinded to clinical results, evaluated INHBA expression by semi-quantitative immunohistochemistry. Staining intensity was scored as 0 (negative), 1 (weak), 2 (moderate), or 3 (strong); the positive tumor cell proportion was scored as 0 ( ≤ 5%), 1 (6–25%), 2 (26–50%), 3 (51–75%), or 4 ( > 75%). The final H-score (intensity × proportion) ranged from 0 to 12. ROC curve analysis identified an H-score ≥8 as the optimal cutoff for high INHBA expression. Kaplan–Meier analysis and log-rank test were used to assess the association between INHBA expression and OS.

Additionally, to determine SLC25A10 expression in colon cancer tissues, ethics approval was obtained from the Hunan Cancer Hospital Ethics Committee. Thirty patients pathologically diagnosed with colon cancer between 2023 and 2025 were enrolled.

### Animal experiments

Six-week-old female C57BL/6 mice obtained from the Experimental Animal Center at the Xiangya School of Medicine Affiliated Tumor Hospital, Central South University (Changsha, China) were maintained under a specific pathogen-free (SPF) conditions. Following 1-week acclimatization, exponentially growing transfected MC38 cells were resuspended at 1 × 10⁷ cells/mL in PBS, and mice received a subcutaneous injection of 1 × 10⁶ cells into the left forelimb’s upper area. From day 5 post-inoculation, tumor size was measured every 5 days and volume calculated as (length × width²)/2. On day 20, the mice were euthanized and tumors were excised, photographed, and weighed.

For the animal experiments involving INHBA knockdown with succinate supplementation, we initiated intraperitoneal injections of succinate (20 mg/kg; Sigma, Germany) every 5 days, starting from day 5 after the subcutaneous injection of MC38 cells. On day 20, mice were euthanized and tumors excised.

### Statistical analysis

Statistical analysis and graphs were generated with GraphPad Prism. Two-group comparisons were performed using Student’s *t* test; multiple groups were analyzed by one-way ANOVA. Data are presented as mean ± standard deviations.

## Supplementary information

Sigtrans_Supplementary_Materials

the full uncropped Gels and Blots images

## Data Availability

The bulk RNA sequencing dataset is accessible in the GSA-Human database with accession code HRA013752.
