# Supplementary material for: Inhibin beta A drives colorectal cancer progression through macrophage M2 polarization and mitochondria-dependent ferroptosis suppression
Source: Signal Transduct Target Ther. 2025 Dec 26;10:420. doi: 10.1038/s41392-025-02518-y (PMC12741050; doi:10.1038/s41392-025-02518-y)
Supplement: Supplementary file 1 — Sigtrans_Supplementary_Materials [file 41392_2025_2518_MOESM1_ESM.docx]

Supplementary Materials for

Inhibin beta A drives colorectal cancer progression through macrophage M2 polarization and mitochondria-dependent ferroptosis suppression

Wentao Li, Lin Liang, Siyi Liu, Jingqiong Tang, Shuangyan Ou, Zhijun Yuan, Yanhong Zhou, Xia Yuan

Correspondence to: zhouyanhong@csu.edu.cn; yuanxia@hnca.org.cn

**This PDF file includes:**

Figures. S1 to S44

Tables S1 to S9


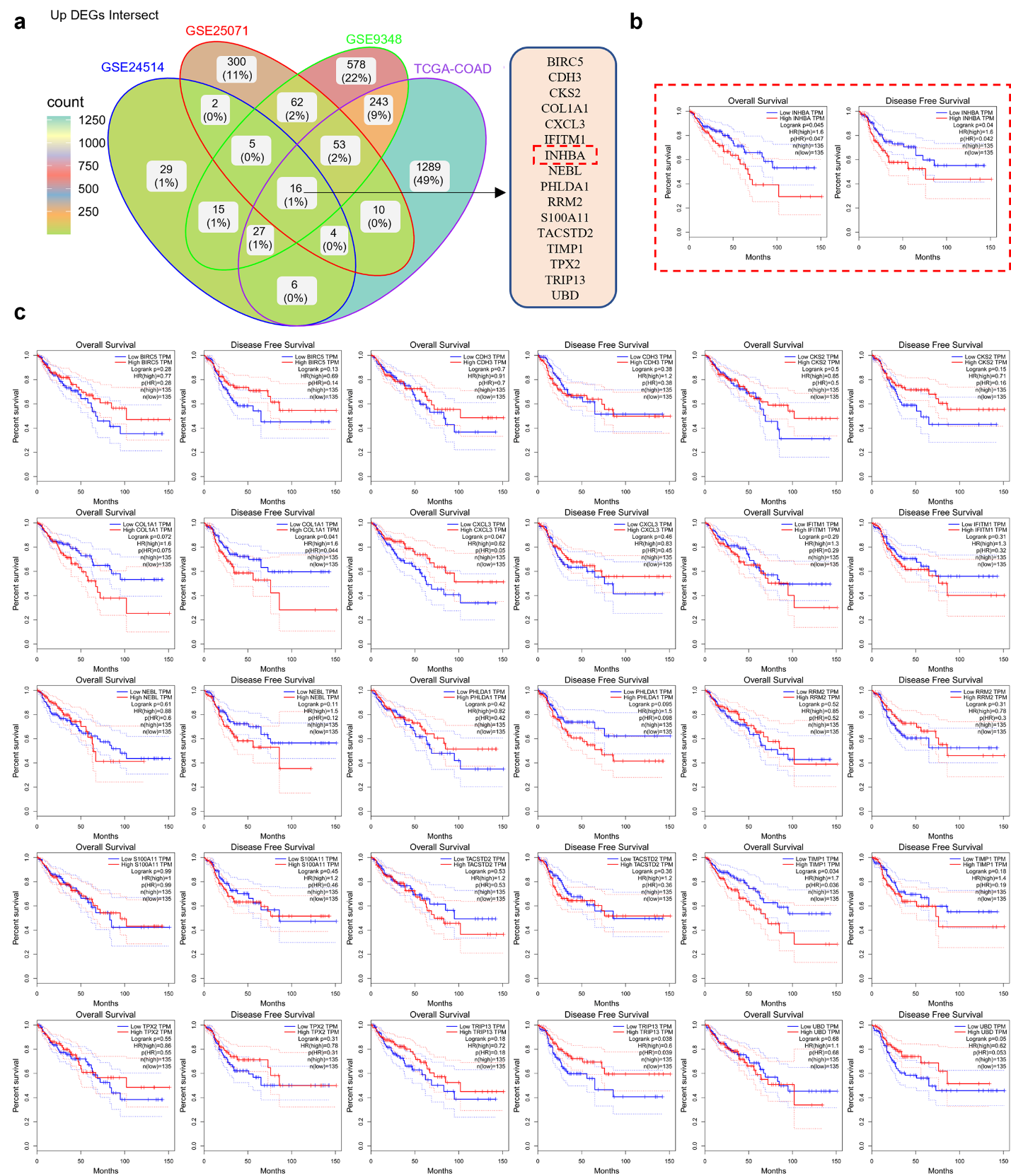


Supplementary Fig. 1. Bioinformatics analysis shows that INHBA is upregulated in CRC and is positively correlated with poor prognosis.

(a) Differential gene mining and Venn diagram construction: Using the TCGA and GEO databases, we identified 16 upregulated differentially expressed genes in CRC and constructed a Venn diagram to display the intersection and union of these genes across different datasets. (b) Survival analysis of the INHBA gene: Using the public online platform GEPIA, we performed OS and DFS analyses for the INHBA gene to assess its prognostic value in CRC. (c) Survival analysis of the other 15 genes: Using the public online platform GEPIA, we performed OS and DFS analyses for the remaining 15 differentially expressed genes to assess their prognostic value in CRC.


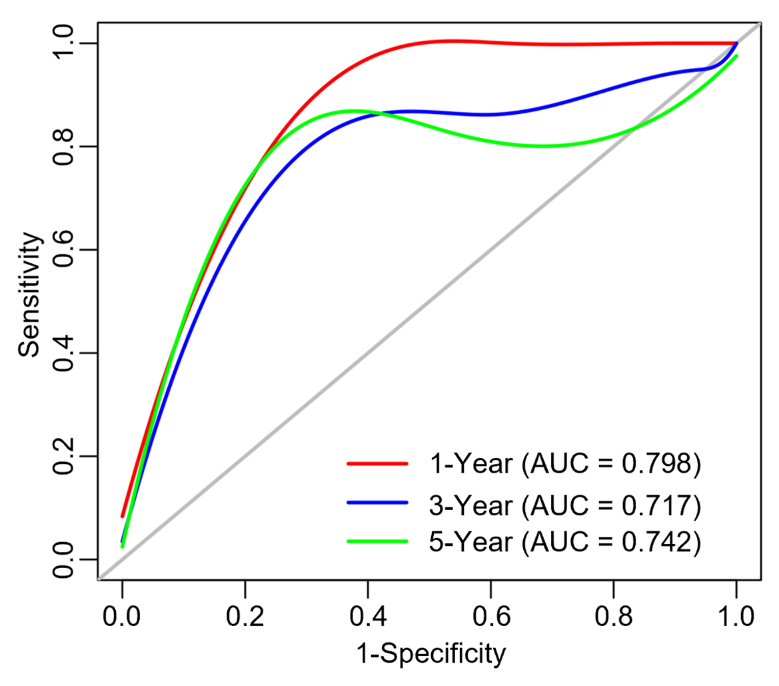


Supplementary Fig. 2. ROC curve analysis of INHBA expression for predicting long-term survival in CRC patients.

ROC curves for 1-, 3-, and 5-year OS in CRC patients were generated based on INHBA IHC scores from tissue microarrays. The AUCs were 0.798, 0.717, and 0.742, respectively, indicating that INHBA expression is a favorable predictor of prognosis.


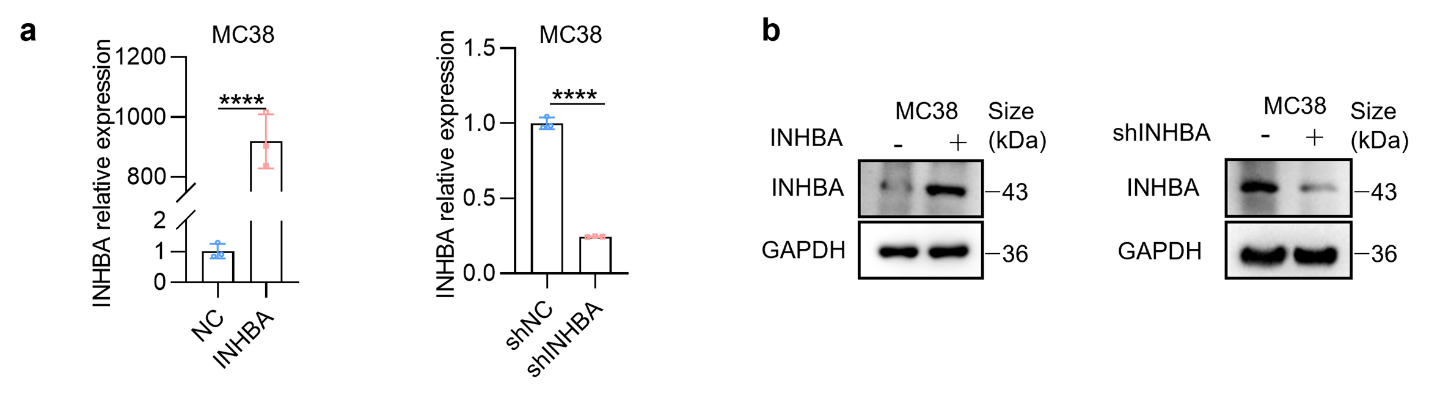


Supplementary Fig. 3. Construction of mouse CRC cell models with INHBA overexpression and knockdown.

(a) qPCR experiments demonstrate the successful construction of INHBA overexpression and knockdown models. (b) Western Blot experiments demonstrate the successful construction of INHBA overexpression and knockdown models. For all the statistical plots, the data are presented as the means ± SD; n = 3 independent experiments. ****P < 0.0001.


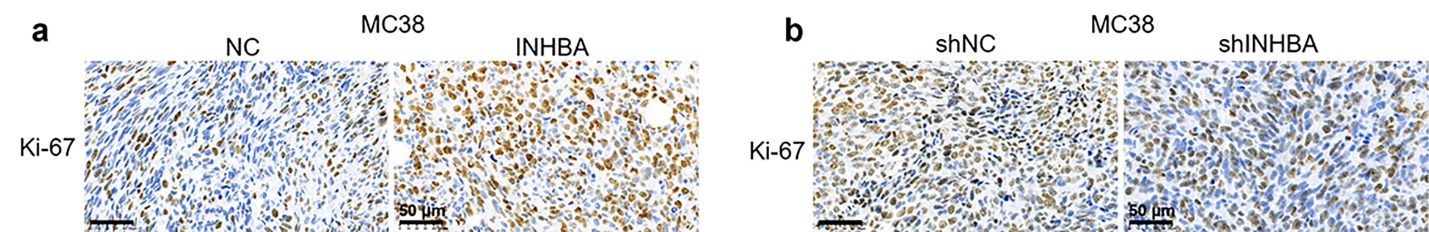


Supplementary Fig. 4. Immunohistochemical detection of the effects of INHBA on Ki-67 in mouse syngeneic tumors.

(a) Effects of INHBA overexpression on Ki-67 in mouse syngeneic tumors. (b) Effects of INHBA knockdown on Ki-67 in mouse syngeneic tumors, with a scale bar of 50 µm.


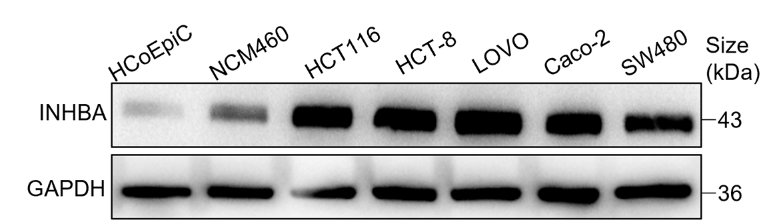


Supplementary Fig. 5. Western Blot detection of INHBA protein expression in two normal colorectal mucosal epithelial cell lines and five colorectal adenocarcinoma cell lines.


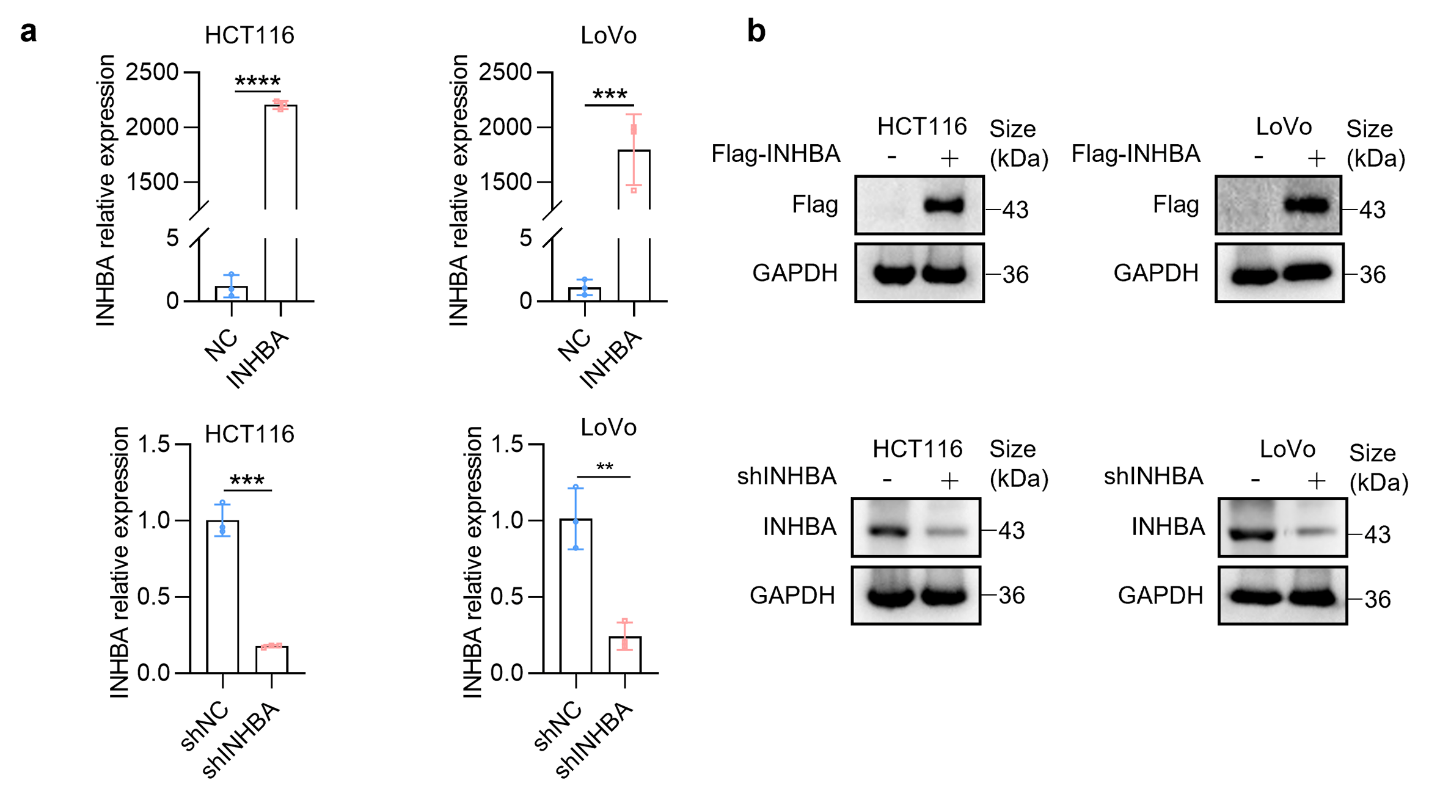


Supplementary Fig. 6. Construction of human CRC cell models with INHBA overexpression and knockdown.

(a) qPCR experiments demonstrate the successful construction of human CRC cell models with INHBA overexpression and knockdown. (b) Western Blot experiments demonstrate the successful construction of human CRC cell models with INHBA overexpression and knockdown. For all the statistical plots, the data are presented as the means ± SD; n = 3 independent experiments. **P < 0.01, ***P < 0.001, ****P < 0.0001.


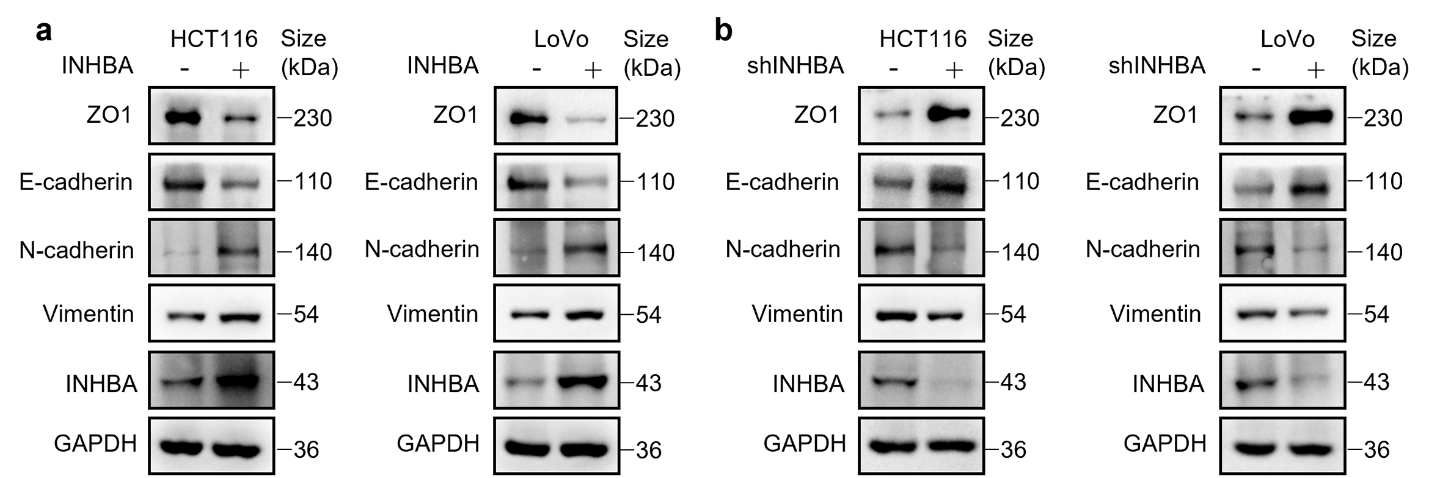


Supplementary Fig. 7. Western Blot analysis of the effects of INHBA on epithelial–mesenchymal transition (EMT) marker protein levels.

(a) Western Blot evaluation of the impact of INHBA overexpression on EMT marker protein levels in HCT116 and LoVo cells. (b) Western Blot evaluation of the impact of INHBA knockdown on EMT marker protein levels in HCT116 and LoVo cells.


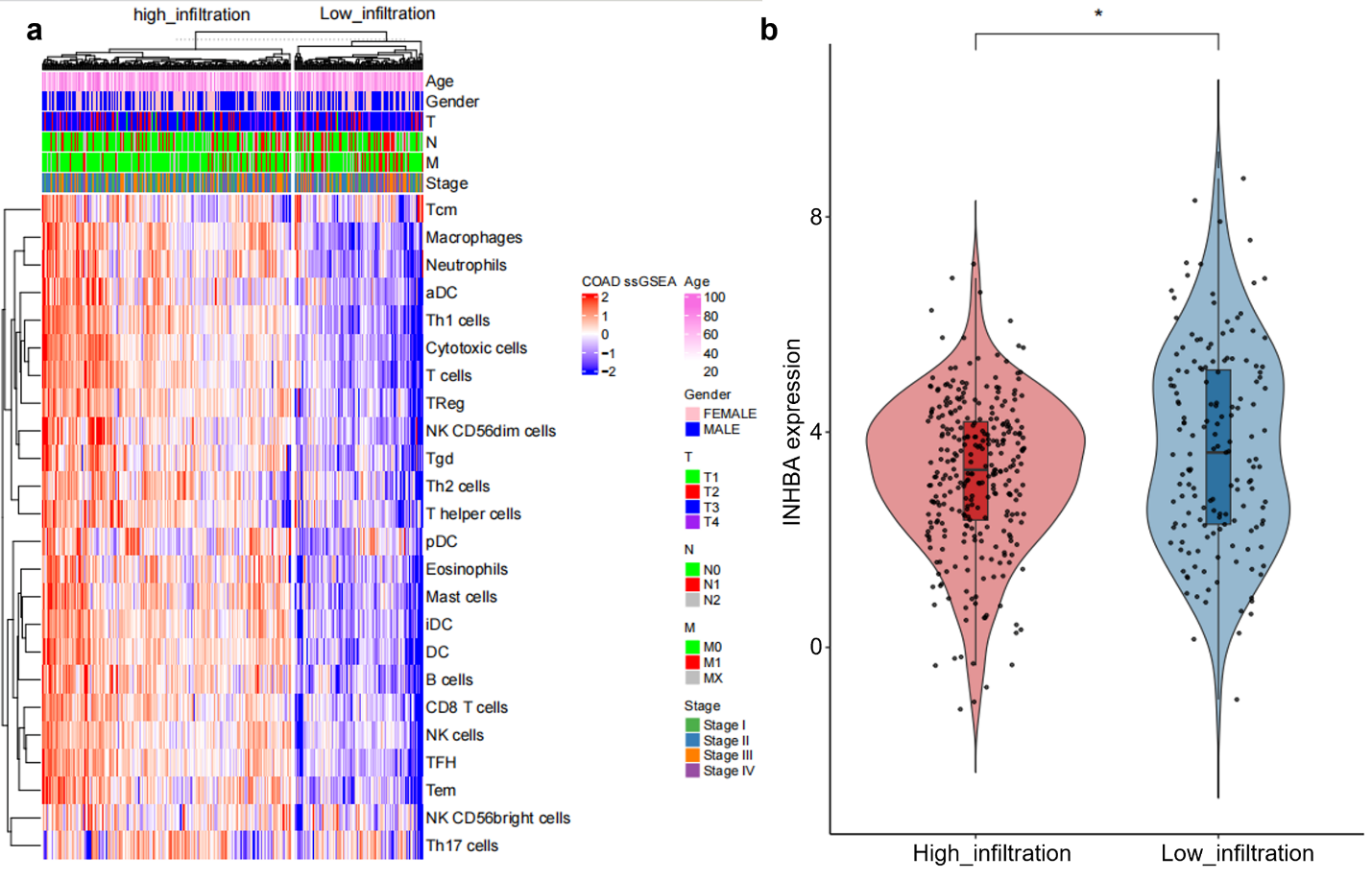


Supplementary Fig. 8. Low immune infiltration correlates with later N, M and overall Stage and elevated INHBA in CRC.

(a) Unsupervised hierarchical clustering of TCGA-COAD samples based on ssGSEA scores of 24 immune-cell signature genes separated tumors into high- and low-immune-infiltration groups; heatmap shows later N stage, M stage and overall Stage in the low-infiltration subset. (b) Mann–Whitney U test revealed significantly higher INHBA expression in the low-infiltration group, suggesting a potential link between high INHBA levels, immune escape and an immunosuppressive microenvironment.


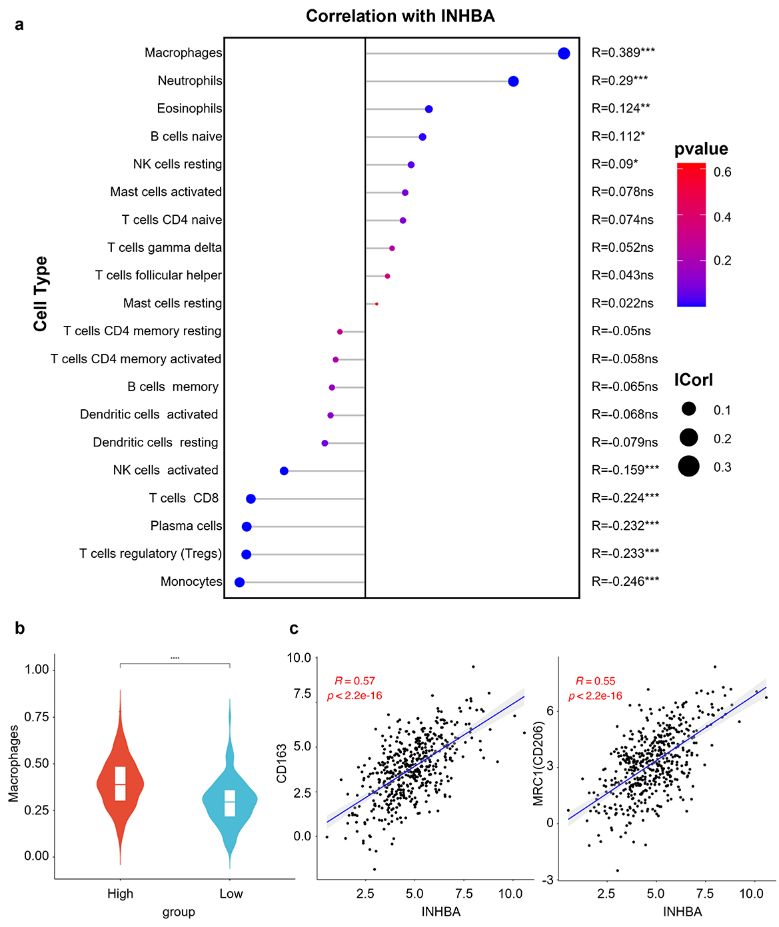


Supplementary Fig. 9. Bioinformatics analysis of the relationship between INHBA and tumor-infiltrating immune cells.

(a) CIBERSORTx analysis of immune cell infiltration abundance: Using the online tool CIBERSORTx to evaluate the abundance of tumor-infiltrating immune cells and analyzing the correlation between INHBA expression and the infiltration levels of various immune cells. (b) Differential analysis of macrophage abundance: Samples were divided into high-expression and low-expression groups based on the median value of INHBA expression. Differential analysis of macrophage abundance was performed between these two groups to reveal the impact of INHBA expression levels on macrophage infiltration. (c) TIMER analysis of the correlation between INHBA and M2 TAM markers: Using the online platform TIMER to analyze the correlation between INHBA and markers of M2-type TAMs, assessing the potential role of INHBA in regulating the tumor immune microenvironment. *P < 0.05, **P < 0.01, ***P < 0.001, ****P < 0.0001, ns = no significance.


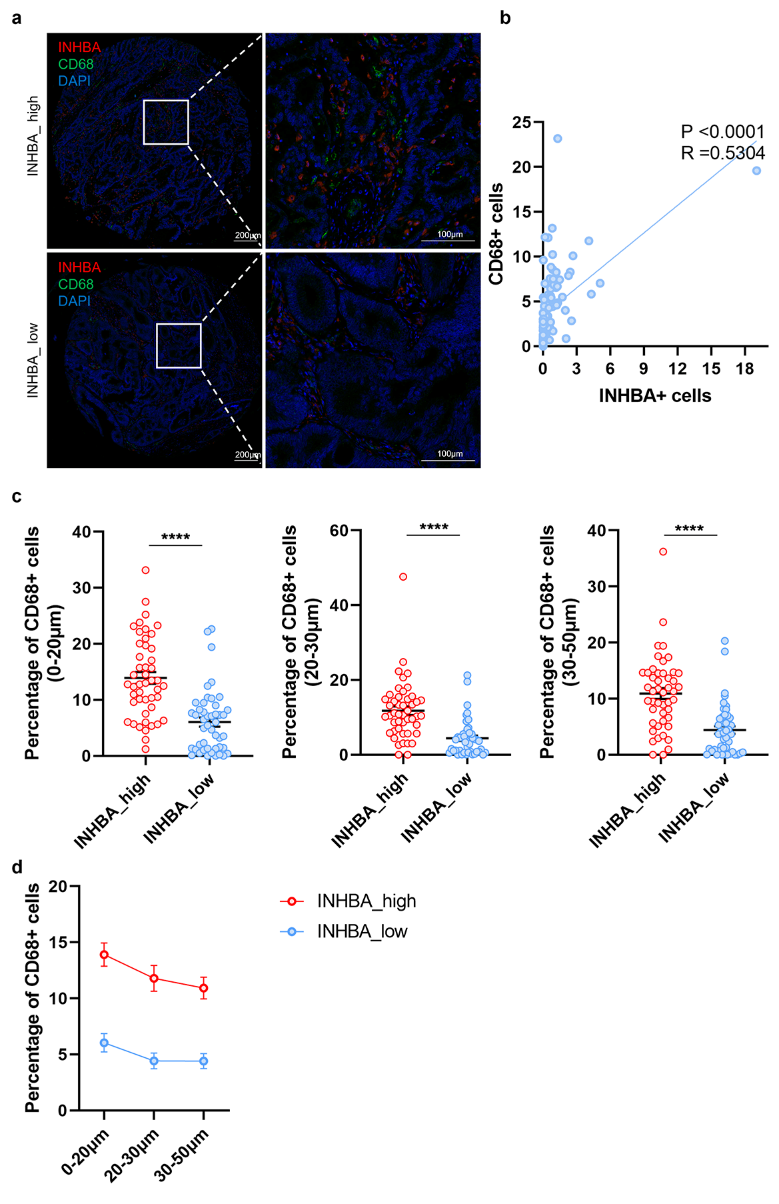


Supplementary Fig. 10. INHBA-high expression positively correlates with CD68⁺ macrophage infiltration and peritumoral distribution.

(a) Representative multiplex-immunofluorescence images showing the spatial distribution of INHBA (red) and CD68⁺ macrophages (green) in human CRC tissue; nuclei counterstained with DAPI (blue). (b) Correlation between INHBA⁺ tumor-cell abundance and CD68⁺ macrophage density across 93 colon-cancer specimens (Pearson R = 0.5304, P < 0.0001). (c) Samples were stratified into INHBA-high (n = 47) and INHBA-low (n = 46) based on median INHBA fluorescence intensity; CD68⁺ macrophage fractions were quantified in three concentric peritumoral zones (0–20 μm, 20–30 μm, 30–50 μm). (d) CD68⁺ macrophage proportions in each zone were significantly higher in the INHBA-high group. For all the statistical plots, the data are presented as the means ± SEM.


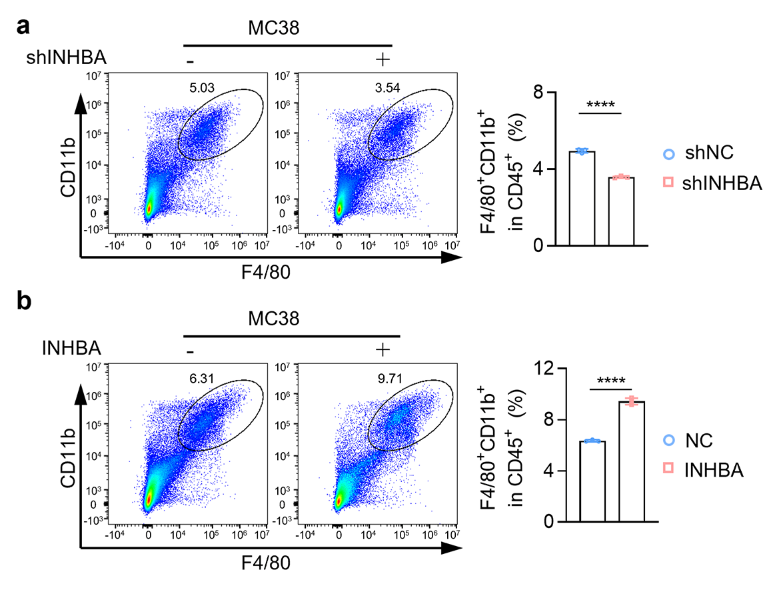


Supplementary Fig. 11. Flow cytometry analysis of the impact of INHBA on macrophage infiltration in mouse CRC tumor grafts.

(a) Flow cytometry analysis of the impact of INHBA knockdown on macrophage infiltration in mouse CRC tumor grafts. (b) Flow cytometry analysis of the impact of INHBA overexpression on macrophage infiltration in mouse CRC tumor grafts. For all the statistical plots, the data are presented as the means ± SD; n = 3 independent experiments. ****P < 0.0001.


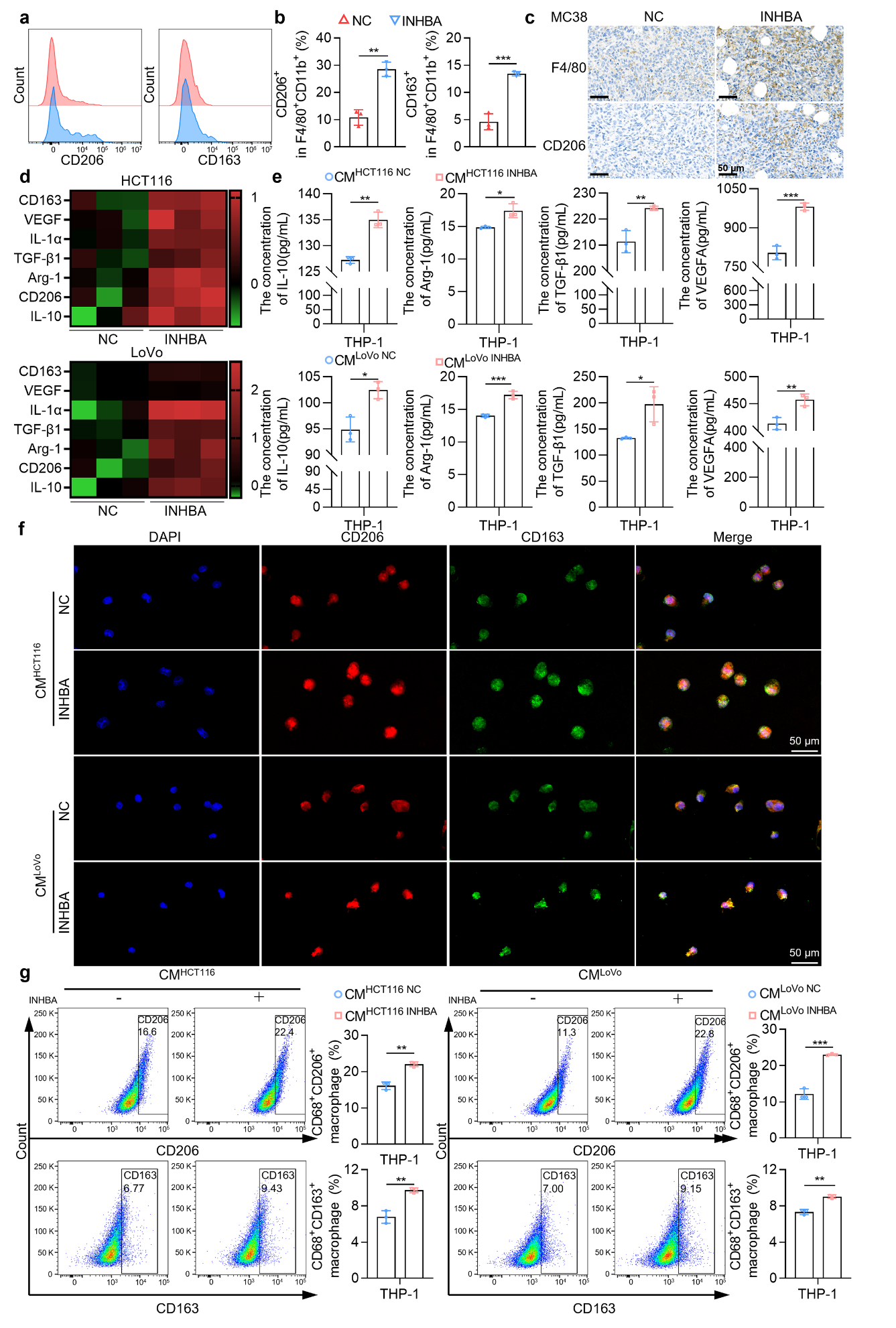


Supplementary Fig. 12. INHBA overexpression promotes the M2 polarization of TAMs in the TME.

(a) Flow cytometry analysis: Detection of the proportions of F4/80⁺CD11b⁺CD206⁺ and F4/80⁺CD11b⁺CD163⁺ macrophages in mouse syngeneic tumor samples with INHBA overexpression. (b) Flow cytometry statistical results: Statistical results of flow cytometry analysis, showing the effects of INHBA overexpression on the proportions of macrophage subpopulations. (c) Immunohistochemical experiments: Detection of the correlation between INHBA expression and the expression of F4/80 and CD206 in mice. Scale bar: 50 µm. (d) qPCR experiments: Detection of the expression changes of M2 polarization-related genes in TAMs after treatment with CM derived from human CRC cells with INHBA overexpression. Results are presented as log2-transformed values. (e) ELISA detection: Measurement of the expression levels of IL-10, Arg-1, TGF-β1, and VEGFA in human macrophages treated with different CMs. (f) Immunofluorescence experiments: Detection of the fluorescence intensity of CD206 (red fluorescence) and CD163 (green fluorescence) in human macrophages treated with different CMs. Scale bar: 50 µm. (g) Flow cytometry analysis: Detection of the proportions of CD68⁺CD206⁺ macrophages and CD68⁺CD163⁺ macrophages in human macrophages treated with different CMs. For all the statistical plots, the data are presented as the means ± SD; n = 3 independent experiments. *P < 0.05, **P < 0.01, ***P < 0.001.


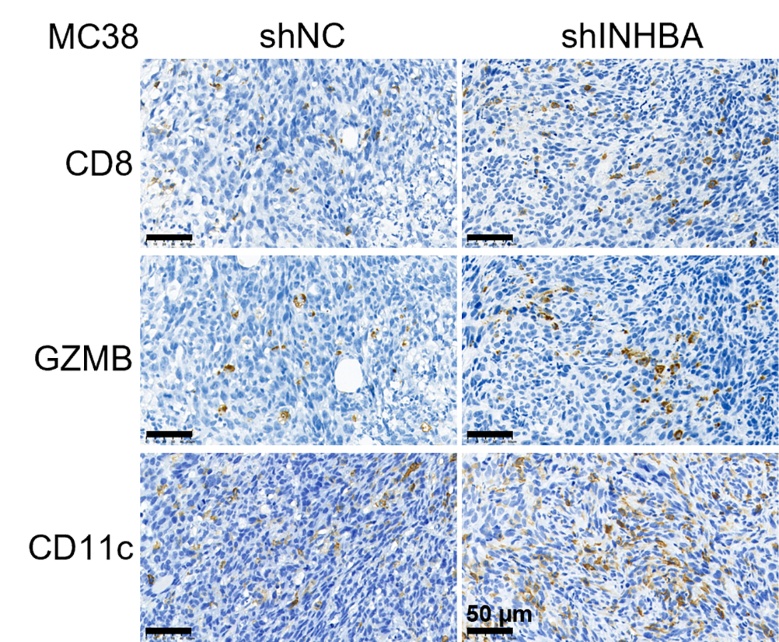


Supplementary Fig. 13. Knock-down of INHBA enhances CD8⁺ T-cell and DC infiltration in MC38 tumors.

IHC shows that INHBA knockdown increases the infiltration of CD8⁺ T cells and DCs in MC38 syngeneic mouse tumors. Scale bar: 50 µm.


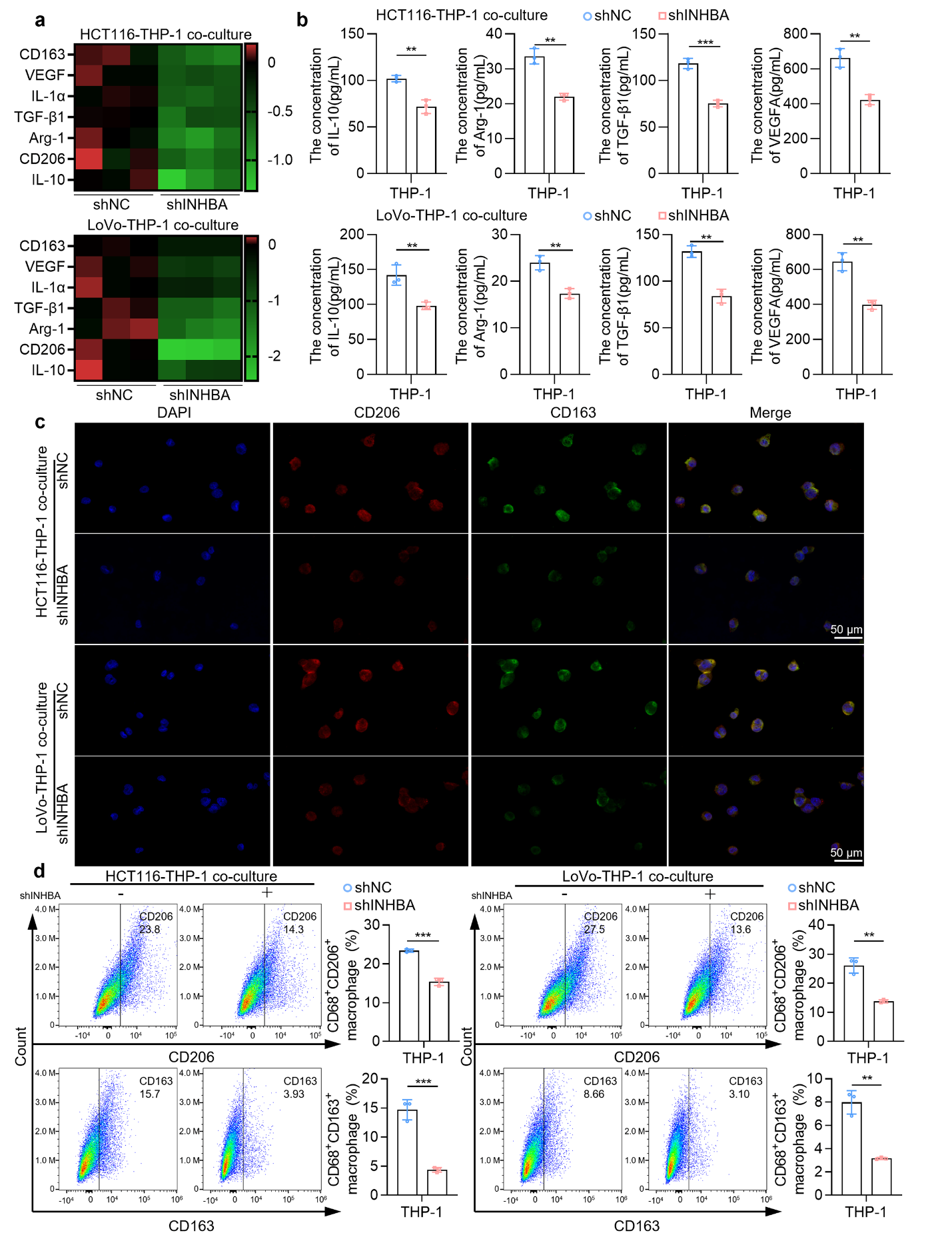


Supplementary Fig .14. INHBA knockdown inhibits M2 polarization of TAMs in the TME via a transwell co-culture system.

CRC cells with INHBA knockdown were seeded in 24-mm-diameter inserts containing 0.4-μm pores, transferred to 6-well plates pre-seeded with THP-1 cells that had been induced with 100 ng/ml PMA for 24 hours, and co-cultured for an additional 24 hours. The resulting macrophages were subjected to the following assays: (a) qPCR experiments: Detection of the expression changes of M2 polarization-related genes in TAMs; results are shown as log2-transformed values. (b) ELISA detection: Measurement of the expression levels of IL-10, Arg-1, TGF-β1 and VEGFA in human macrophages. (c) Immunofluorescence experiments: Detection of the fluorescence intensity of CD206 (red fluorescence) and CD163 (green fluorescence) in human macrophages. Scale bar: 50 µm. (d) Flow cytometry analysis: Detection of proportions of CD68⁺CD206⁺ and CD68⁺CD163⁺ macrophages among human macrophages. For all the statistical plots, the data are presented as the means ± SD; n = 3 independent experiments. **P < 0.01, ***P < 0.001.


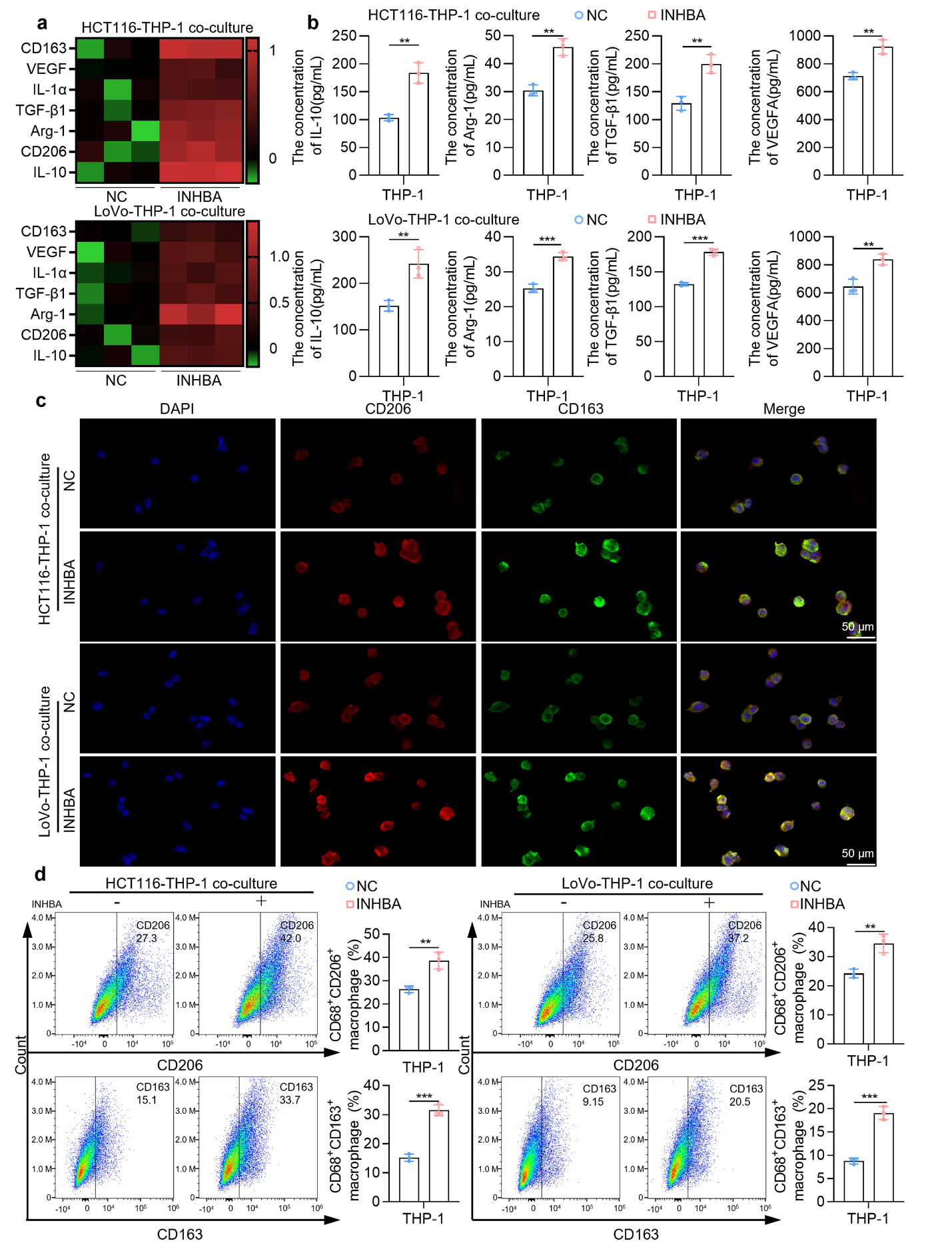


Supplementary Fig. 15. INHBA overexpression promotes M2 polarization of TAMs in the TME via a transwell co-culture system.

CRC cells with INHBA overexpression were seeded in 24-mm-diameter inserts containing 0.4-μm pores, transferred to 6-well plates pre-seeded with THP-1 cells that had been induced with 100 ng/ml PMA for 24 hours, and co-cultured for an additional 24 hours. The resulting macrophages were subjected to the following assays: (a) qPCR experiments: Detection of the expression changes of M2 polarization-related genes in TAMs; results are shown as log2-transformed values. (b) ELISA detection: Measurement of the expression levels of IL-10, Arg-1, TGF-β1 and VEGFA in human macrophages. (c) Immunofluorescence experiments: Detection of the fluorescence intensity of CD206 (red fluorescence) and CD163 (green fluorescence) in human macrophages. Scale bar: 50 µm. (d) Flow cytometry analysis: Detection of proportions of CD68⁺CD206⁺ and CD68⁺CD163⁺ macrophages among human macrophages. For all the statistical plots, the data are presented as the means ± SD; n = 3 independent experiments. **P < 0.01, ***P < 0.001.


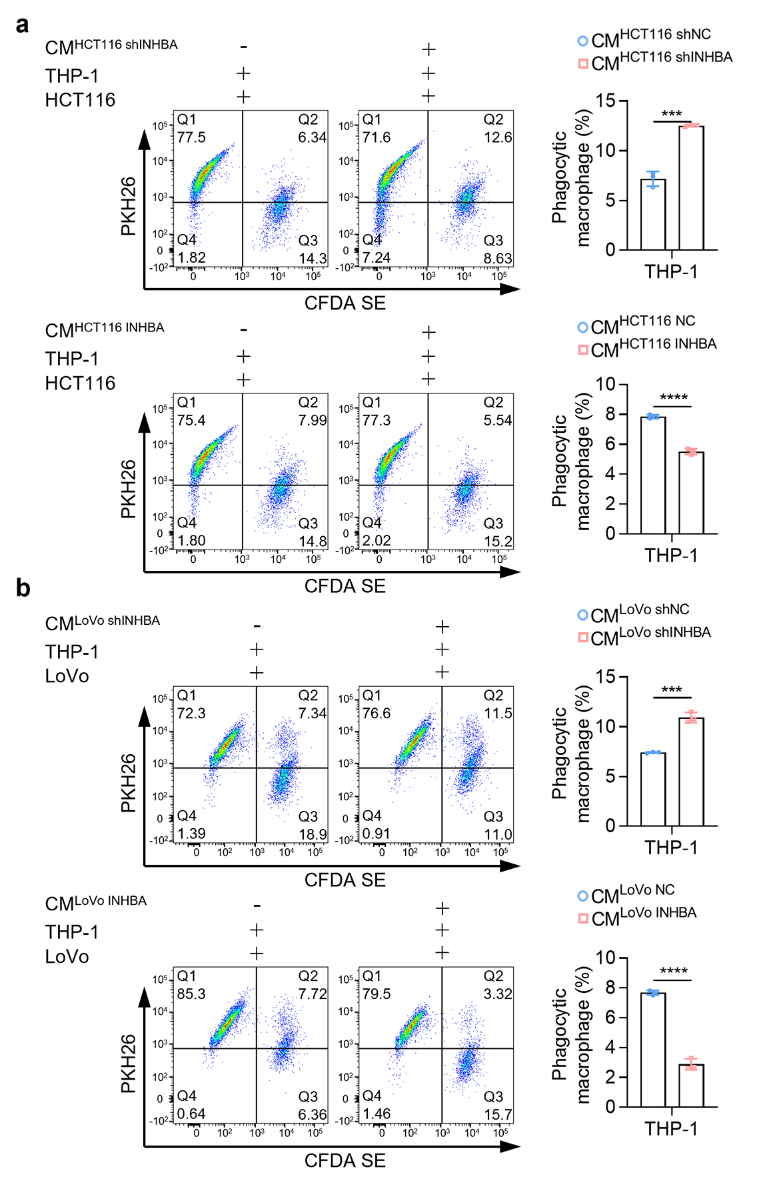


Supplementary Fig. 16. Flow cytometry analysis of the phagocytic capacity of macrophages against tumor cells after treatment with CM from CRC cells with INHBA knockdown or overexpression.

(a) CM from HCT116 cells with INHBA knockdown or overexpression was used to stimulate THP-1 cells that had been differentiated into macrophages by 24-hour PMA treatment. These induced macrophages were then co-cultured with HCT116 cells, and their tumor-phagocytic capacity was evaluated by flow cytometry. (b) CM from LoVo cells with INHBA knockdown or overexpression was used to stimulate THP-1 cells that had been differentiated into macrophages by 24-hour PMA treatment. These induced macrophages were then co-cultured with LoVo cells, and their tumor-phagocytic capacity was evaluated by flow cytometry. For all the statistical plots, the data are presented as the means ± SD; n = 3 independent experiments. ***P < 0.001, ****P < 0.0001.


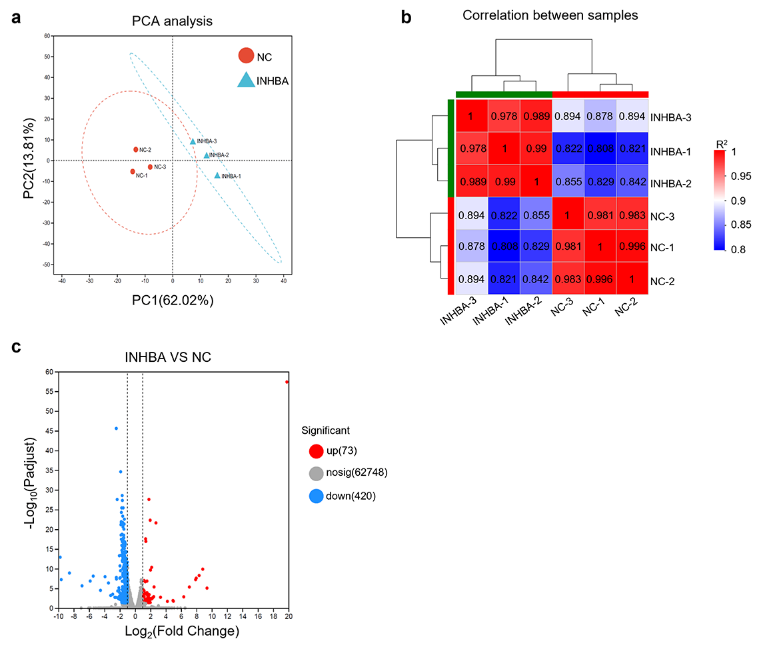


Supplementary Fig. 17. Transcriptome sequencing results of HCT116 cells with INHBA overexpression and control.

(a) PCA results of transcriptome sequencing. (b) Heatmap of sample correlation. (c) Volcano plot of differential expression.


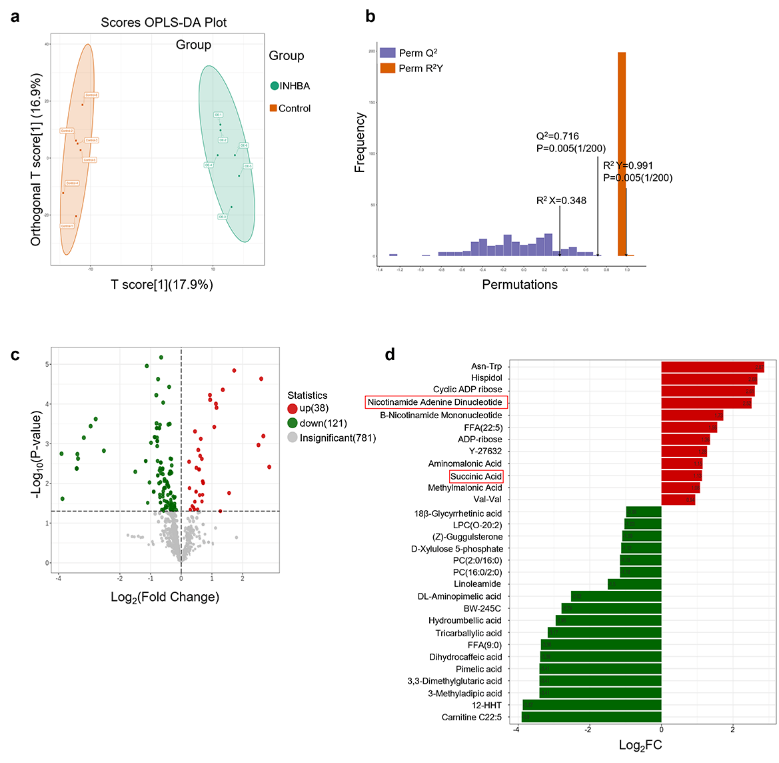


Supplementary Fig. 18. Results of widely targeted metabolomics sequencing in HCT116 cells with INHBA overexpression and control.

(a) OPLS-DA results. (b) Validation results of the OPLS-DA model. (c) Volcano plot of differential metabolites. (d) Bar chart of differential metabolites.


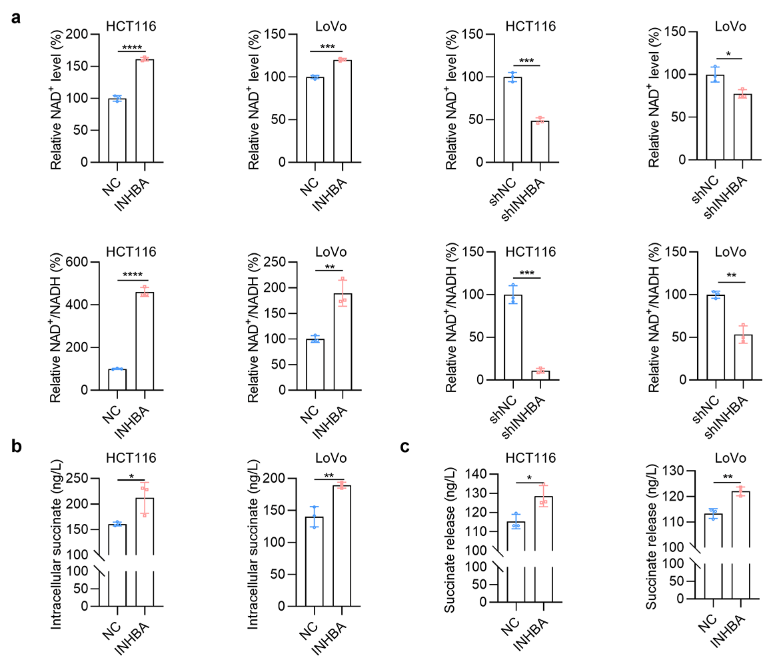


Supplementary Fig. 19. ELISA detection of NAD⁺ and succinate levels after INHBA overexpression or knockdown.

(a) Intracellular NAD⁺ and NAD⁺/NADH levels in CRC cells after INHBA overexpression or knockdown. (b) Intracellular succinate levels in CRC cells after INHBA overexpression. (c) Extracellular release of succinate in CRC cells after INHBA overexpression. For all the statistical plots, the data are presented as the means ± SD; n = 3 independent experiments. *P < 0.05, **P < 0.01, ***P < 0.001, ****P < 0.0001.


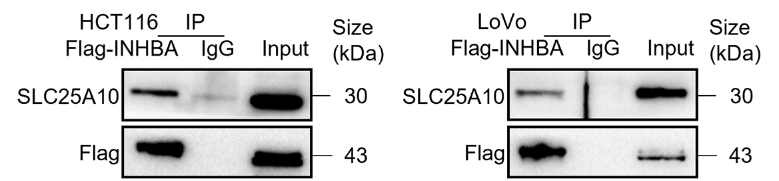


Supplementary Fig. 20. IP experiments to detect the interaction between INHBA and SLC25A10 in CRC cells.

IP: Flag-INHBA indicates IP experiments using anti-Flag antibody to detect interaction proteins of INHBA. IgG serves as a negative control. Input represents the total cell lysate.


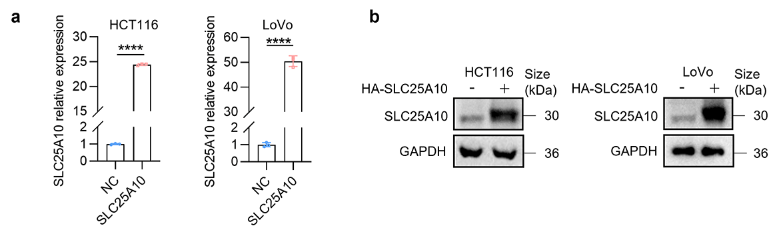


Supplementary Fig. 21. Construction of human CRC cell models with SLC25A10 overexpression.

(a) qPCR experiments demonstrate the successful construction of human CRC cell models with SLC25A10 overexpression. (b) Western Blot experiments demonstrate the successful construction of human CRC cell models with SLC25A10 overexpression. For all the statistical plots, the data are presented as the means ± SD; n = 3 independent experiments. ****P < 0.0001.


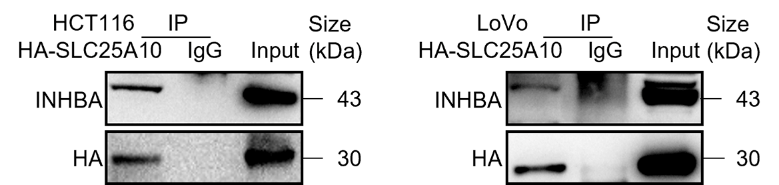


Supplementary Fig. 22. IP experiments to detect the interaction between SLC25A10 and INHBA in CRC cells.

IP: HA-SLC25A10 indicates IP experiments using anti-HA antibody to detect interaction proteins of SLC25A10. IgG serves as a negative control. Input represents the total cell lysate.


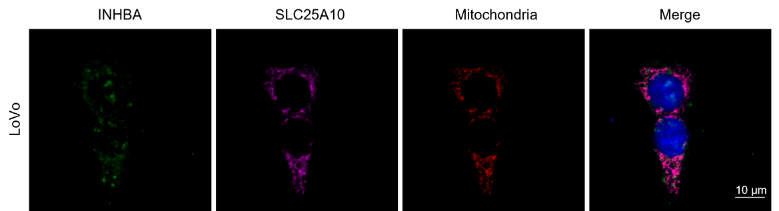


Supplementary Fig. 23. Immunofluorescence assay to demonstrate mitochondrial co-localization of INHBA and SLC25A10 in LoVo cells.

After transfecting LoVo cells with the pDsRed2-Mito plasmid to label mitochondria, immunofluorescence was observed by laser confocal microscopy to verify the mitochondrial co-localization of INHBA and SLC25A10.


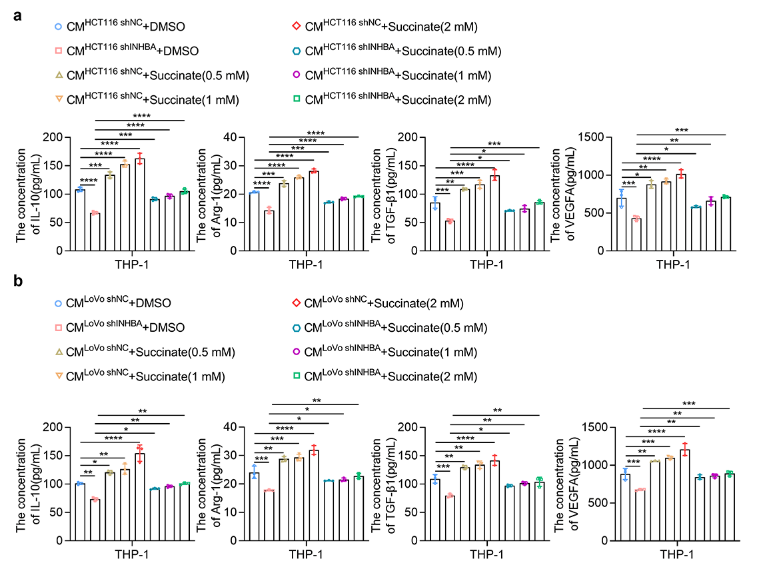


Supplementary Fig. 24. Exogenous succinate supplementation dose-dependently reverses the CM-induced reduction in macrophage secretion of IL-10, Arg-1, TGF-β1, and VEGFA from INHBA-knockdown cells.

(a) After PMA-induced differentiation, THP-1 macrophages were stimulated with CM from INHBA-knockdown HCT116 cells supplemented with 0.5, 1, or 2 mM exogenous succinate, followed by ELISA quantification of IL-10, Arg-1, TGF-β1, and VEGFA. (b) After PMA-induced differentiation, THP-1 macrophages were stimulated with CM from INHBA-knockdown LoVo cells supplemented with 0.5, 1, or 2 mM exogenous succinate, followed by ELISA quantification of IL-10, Arg-1, TGF-β1, and VEGFA. For all the statistical plots, the data are presented as the means ± SD; n = 3 independent experiments. *P < 0.05, **P < 0.01, ***P < 0.001, ****P < 0.0001.


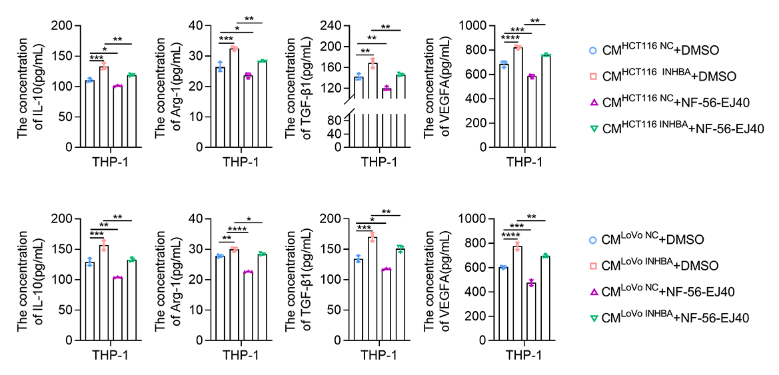


Supplementary Fig. 25. Exogenous addition of NF-56-EJ40 partially reverses the increased secretion of IL-10, Arg-1, TGF-β1, and VEGFA in macrophages induced by CM from INHBA-overexpressing cells.

Exogenous addition of NF-56-EJ40 at 1 μM, followed by ELISA detection of IL-10, Arg-1, TGF-β1, and VEGFA expression in macrophages. For all the statistical plots, the data are presented as the means ± SD; n = 3 independent experiments. *P < 0.05, **P < 0.01, ***P < 0.001, ****P < 0.0001.


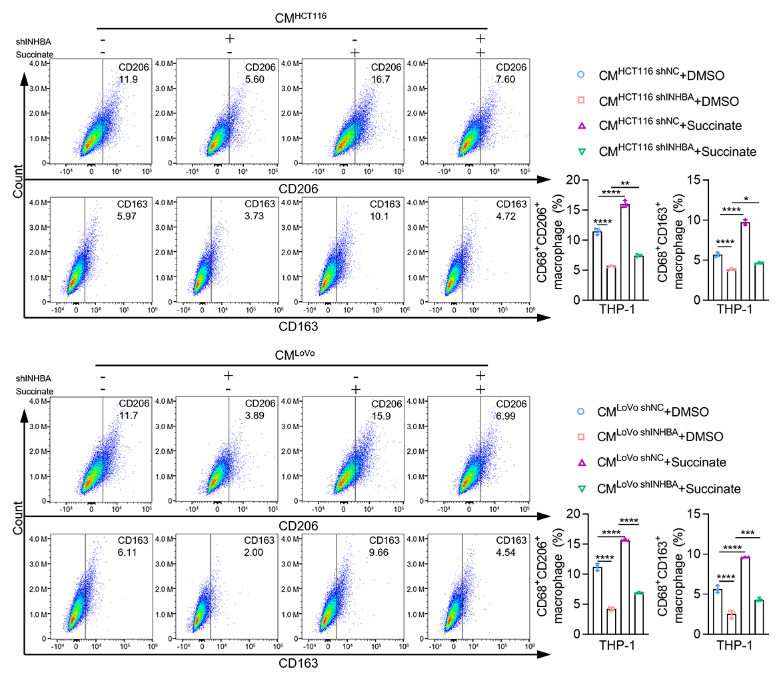


Supplementary Fig. 26. Exogenous supplementation of succinate partially reverses the decreased proportions of CD68⁺CD206⁺ and CD68⁺CD163⁺ macrophages induced by CM from INHBA-knockdown cells.

INHBA was knocked down in cells, and exogenous succinate was added. Flow cytometry was used to detect the proportions of CD68⁺CD206⁺ and CD68⁺CD163⁺ macrophages. For all the statistical plots, the data are presented as the means ± SD; n = 3 independent experiments. *P < 0.05, **P < 0.01, ***P < 0.001, ****P < 0.0001.


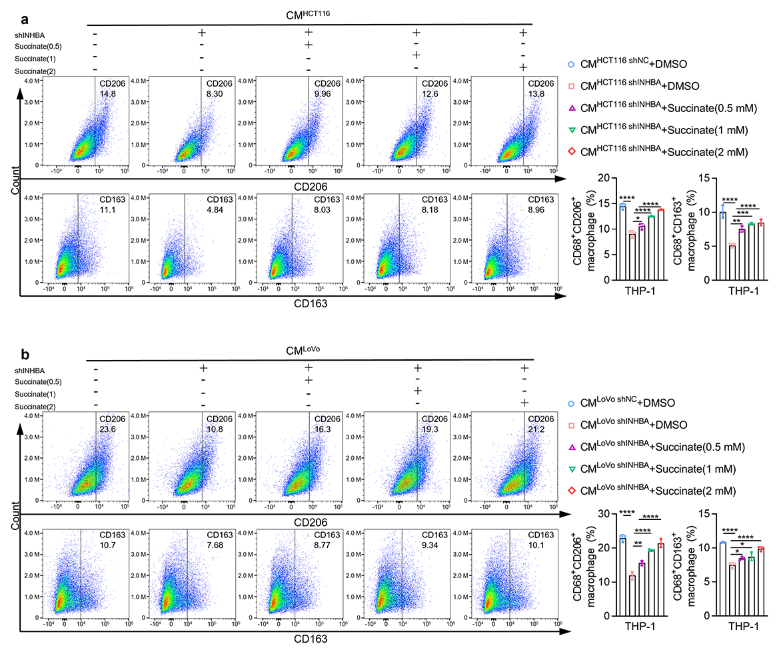


Supplementary Fig. 27. Exogenous succinate supplementation dose-dependently reverses the CM-induced reduction in CD68⁺CD206⁺ and CD68⁺CD163⁺ macrophage proportions from INHBA-knockdown cells.

(a) After PMA-induced differentiation, THP-1 macrophages were stimulated with CM from INHBA-knockdown HCT116 cells supplemented with 0.5, 1, or 2 mM exogenous succinate, followed by flow cytometry analysis of the proportions of CD68⁺CD206⁺ and CD68⁺CD163⁺ macrophages. (b) After PMA-induced differentiation, THP-1 macrophages were stimulated with CM from INHBA-knockdown LoVo cells supplemented with 0.5, 1, or 2 mM exogenous succinate, followed by flow cytometry analysis of the proportions of CD68⁺CD206⁺ and CD68⁺CD163⁺ macrophages. For all the statistical plots, the data are presented as the means ± SD; n = 3 independent experiments. *P < 0.05, **P < 0.01, ***P < 0.001, ****P < 0.0001.


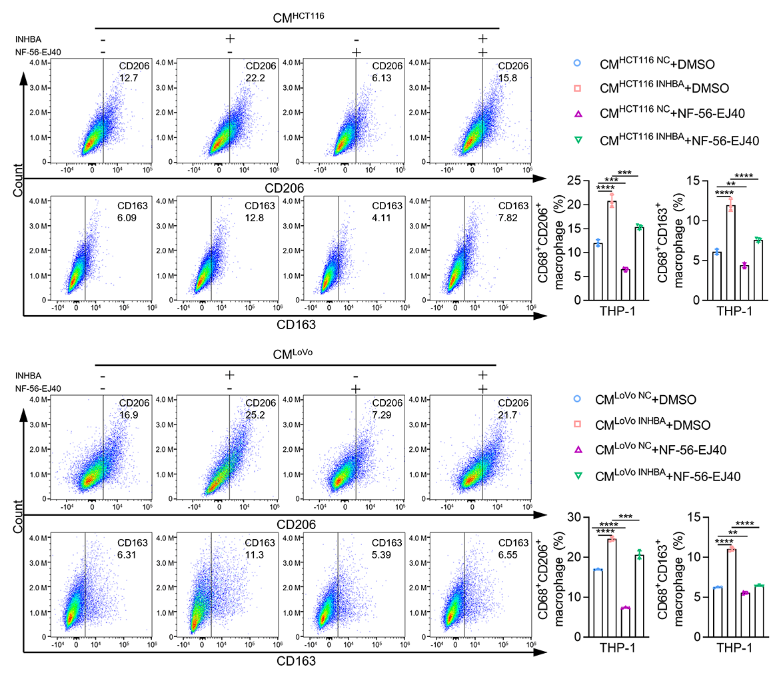


Supplementary Fig. 28. Exogenous addition of NF-56-EJ40 partially reverses the increased proportions of CD68⁺CD206⁺ and CD68⁺CD163⁺ macrophages induced by CM from INHBA-overexpressing cells.

INHBA was overexpressed in cells, and exogenous NF-56-EJ40 at 1 μM was added. Flow cytometry was used to detect the proportions of CD68⁺CD206⁺ and CD68⁺CD163⁺ macrophages. For all the statistical plots, the data are presented as the means ± SD; n = 3 independent experiments. **P < 0.01, ***P < 0.001, ****P < 0.0001.


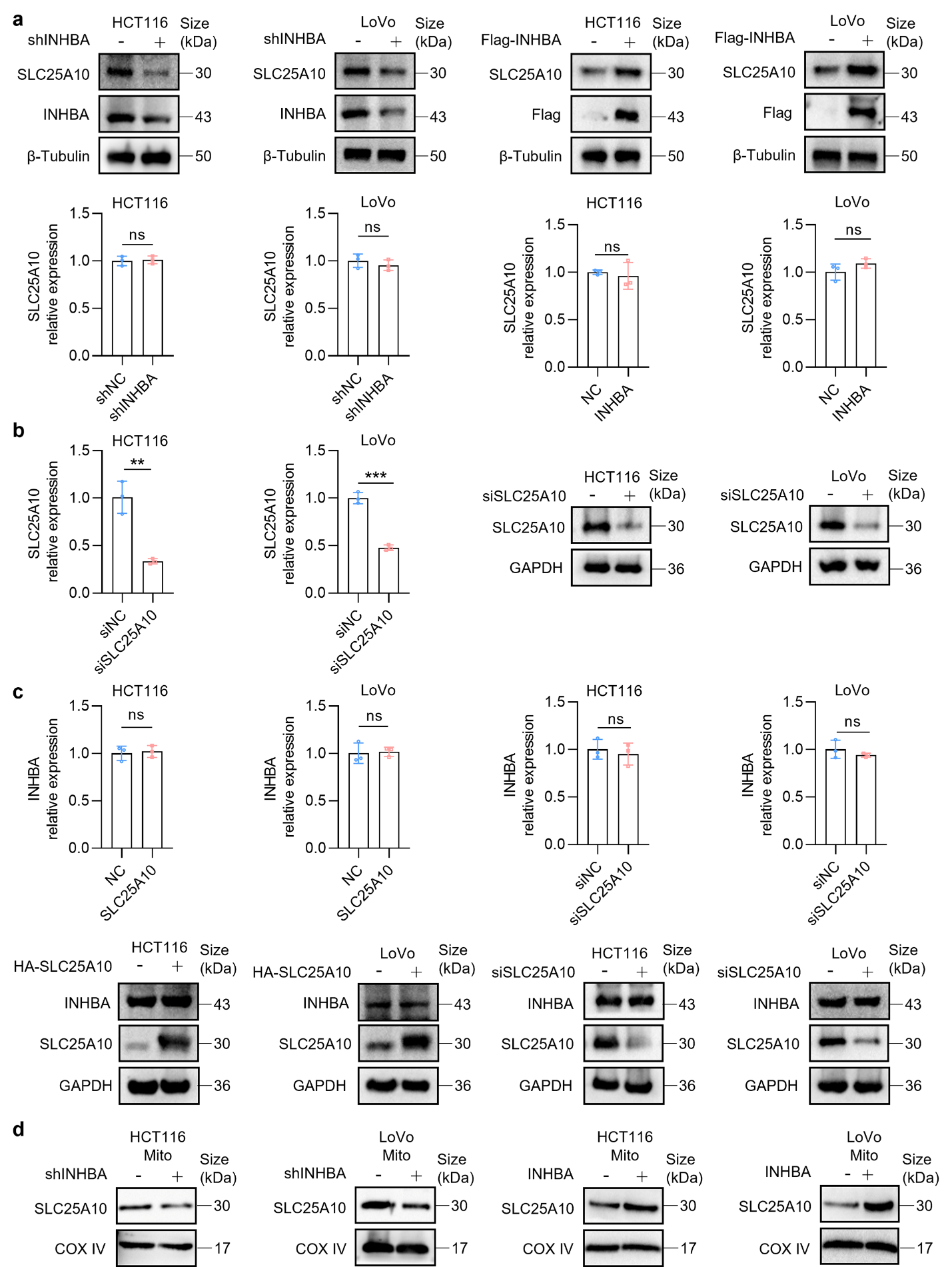


Supplementary Fig. 29. INHBA positively regulates the total protein and mitochondrial protein levels of SLC25A10.

(a) Western Blot and qPCR experiments: Verification of the effects of INHBA knockdown or overexpression on SLC25A10 protein and mRNA levels using Western Blot and qPCR experiments. (b) Validation of SLC25A10 knockdown model: Confirmation of the successful construction of the SLC25A10 knockdown model in human CRC cells using qPCR and Western Blot experiments. (c) Effects of SLC25A10 on INHBA: Verification of the effects of SLC25A10 overexpression or knockdown on INHBA mRNA and protein levels using qPCR and Western Blot experiments. (d) Validation of mitochondrial SLC25A10 protein levels: Confirmation of the effects of INHBA knockdown or overexpression on mitochondrial SLC25A10 protein levels using Western Blot experiments. For all the statistical plots, the data are presented as the means ± SD; n = 3 independent experiments. **P < 0.01, ***P < 0.001, ns = no significance.


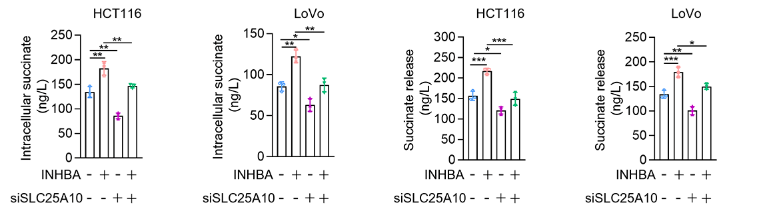


Supplementary Fig. 30. Knockdown of SLC25A10 partially reverses the increased intracellular accumulation and release of succinate caused by INHBA overexpression.

INHBA was overexpressed in cells, while SLC25A10 was knocked down. ELISA was used to detect the intracellular accumulation and release of succinate in CRC cells. For all the statistical plots, the data are presented as the means ± SD; n = 3 independent experiments. *P < 0.05, **P < 0.01, ***P < 0.001.


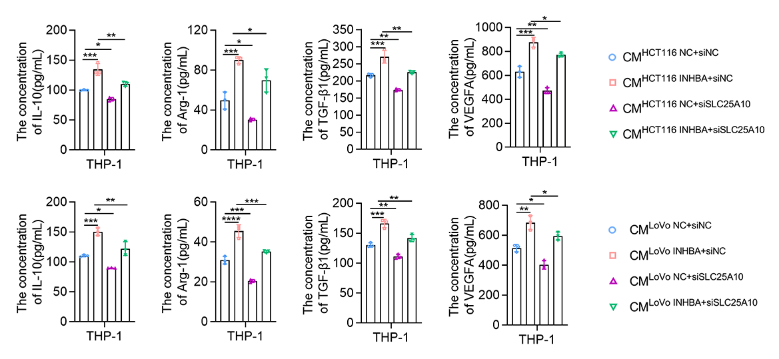


Supplementary Fig. 31. Knockdown of SLC25A10 partially reverses the increased secretion of IL-10, Arg-1, TGF-β1, and VEGFA in macrophages induced by CM from INHBA-overexpressing cells.

Macrophages were cultured with CM derived from human CRC cells that overexpressed INHBA and had SLC25A10 knocked down. ELISA was used to detect the expression of IL-10, Arg-1, TGF-β1, and VEGFA. For all the statistical plots, the data are presented as the means ± SD; n = 3 independent experiments. *P < 0.05, **P < 0.01, ***P < 0.001, ****P < 0.0001.


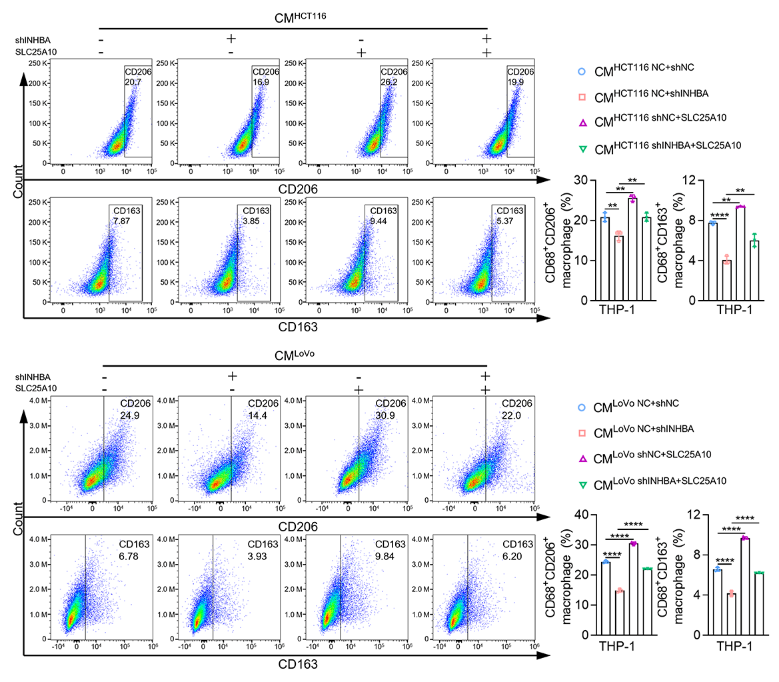


Supplementary Fig. 32. Restoration of SLC25A10 expression partially reverses the decreased proportions of CD68⁺CD206⁺ and CD68⁺CD163⁺ macrophages induced by CM from INHBA-knockdown cells.

Macrophages were cultured with CM derived from human CRC cells that had INHBA knocked down and SLC25A10 expression restored. Flow cytometry was used to detect the proportions of CD68⁺CD206⁺ and CD68⁺CD163⁺ macrophages. For all the statistical plots, the data are presented as the means ± SD; n = 3 independent experiments. **P < 0.01, ****P < 0.0001.


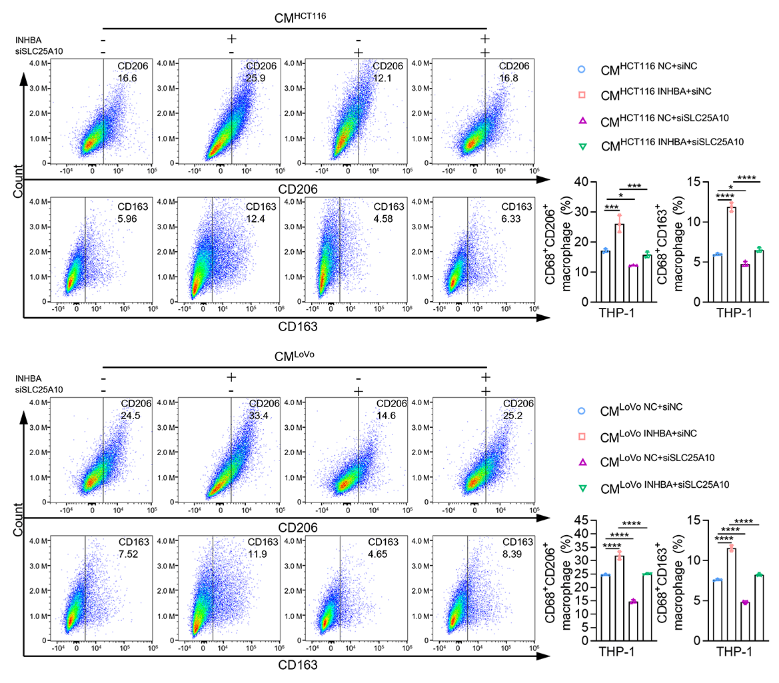


Supplementary Fig. 33. Knockdown of SLC25A10 partially reverses the increased proportions of CD68⁺CD206⁺ and CD68⁺CD163⁺ macrophages induced by CM from INHBA-overexpressing cells.

Macrophages were cultured with CM derived from human CRC cells that overexpressed INHBA and had SLC25A10 knocked down. Flow cytometry was used to detect the proportions of CD68⁺CD206⁺ and CD68⁺CD163⁺ macrophages. For all the statistical plots, the data are presented as the means ± SD; n = 3 independent experiments. *P < 0.05, ***P < 0.001, ****P < 0.0001.


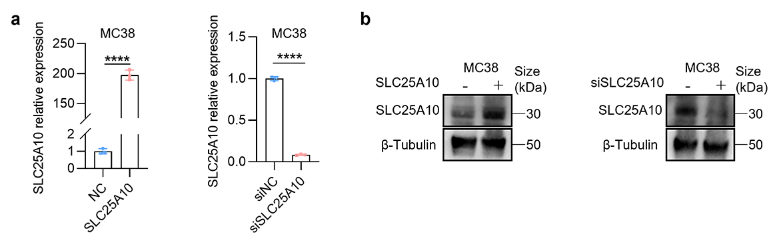


Supplementary Fig. 34. Construction of mouse CRC cell models with SLC25A10 overexpression and knockdown.

(a) qPCR experiments demonstrate the successful construction of mouse CRC cell models with SLC25A10 overexpression and knockdown. (b) Western Blot experiments demonstrate the successful construction of mouse CRC cell models with SLC25A10 overexpression and knockdown. For all the statistical plots, the data are presented as the means ± SD; n = 3 independent experiments. ****P < 0.0001.


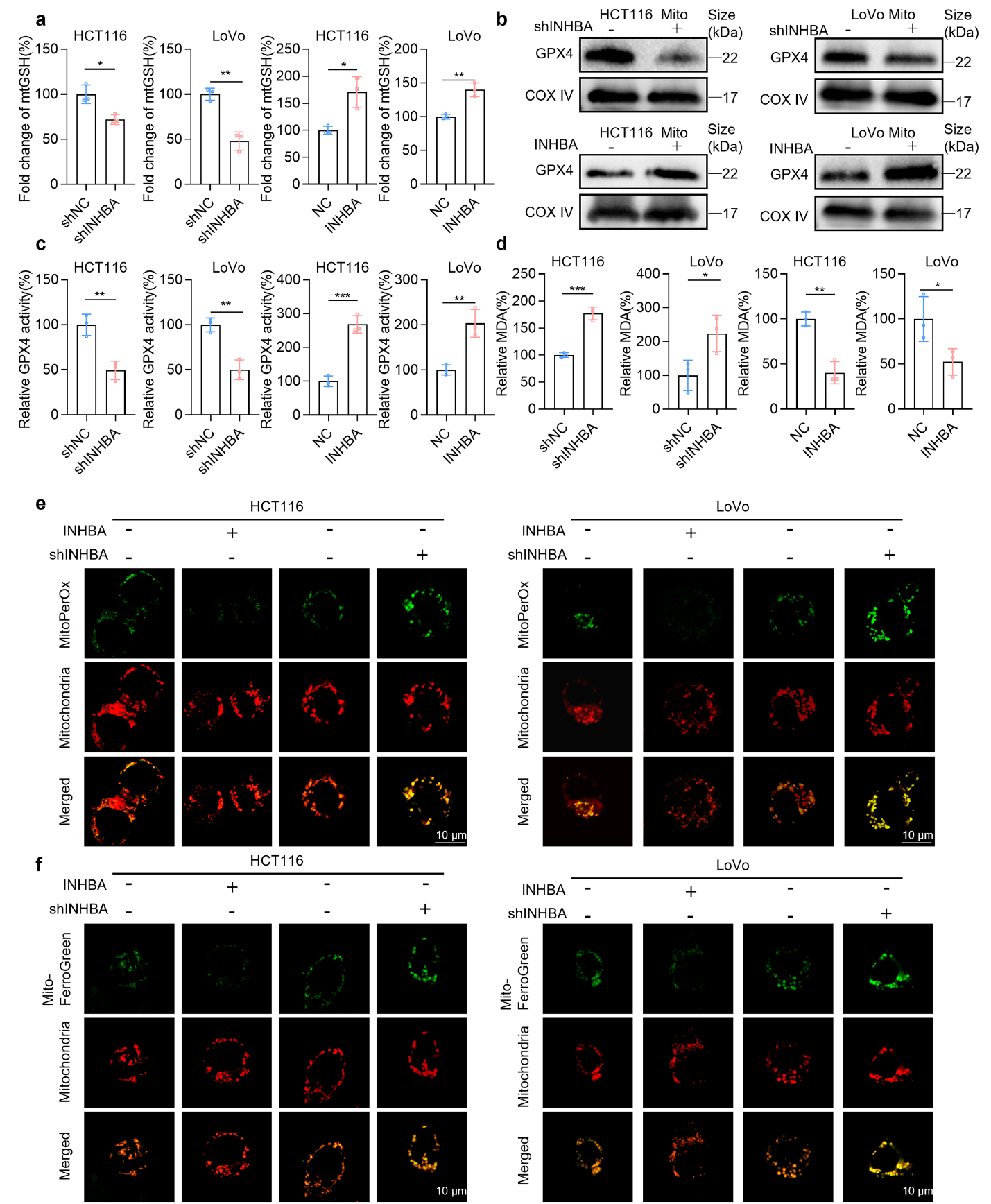


Supplementary Fig. 35. INHBA inhibits mitochondrial ferroptosis by activating the mtGSH/GPX4 axis.

(a) After knocking down or overexpressing INHBA in human CRC cells, mitochondria were isolated with a mitochondrial separation kit and mtGSH levels were measured by a Reduced Glutathione (GSH) Colorimetric Assay Kit. (b) After knocking down or overexpressing INHBA in human CRC cells, mitochondria were isolated with a mitochondrial separation kit and mitochondrial GPX4 protein expression was detected by Western Blot. (c) INHBA was knocked down or overexpressed in human CRC cells, and GPX4 enzymatic activity was measured using the Glutathione Peroxidase 4 (GPX4) Activity Assay Kit. (d) INHBA was knocked down or overexpressed in human CRC cells, and MDA was quantified by HPLC. After overexpressing or knocking down INHBA in human CRC cells, the following fluorescent probes were used for detection: (e) Detection of lipid peroxidation levels: Measurement of lipid peroxidation levels in the mitochondrial inner membrane using MitoPerOx. (f) Detection of ferrous ion changes: Measurement of changes in ferrous ions in mitochondria using Mito-FerroGreen. For all the statistical plots, the data are presented as the means ± SD; n = 3 independent experiments. *P < 0.05, **P < 0.01, ***P < 0.001.


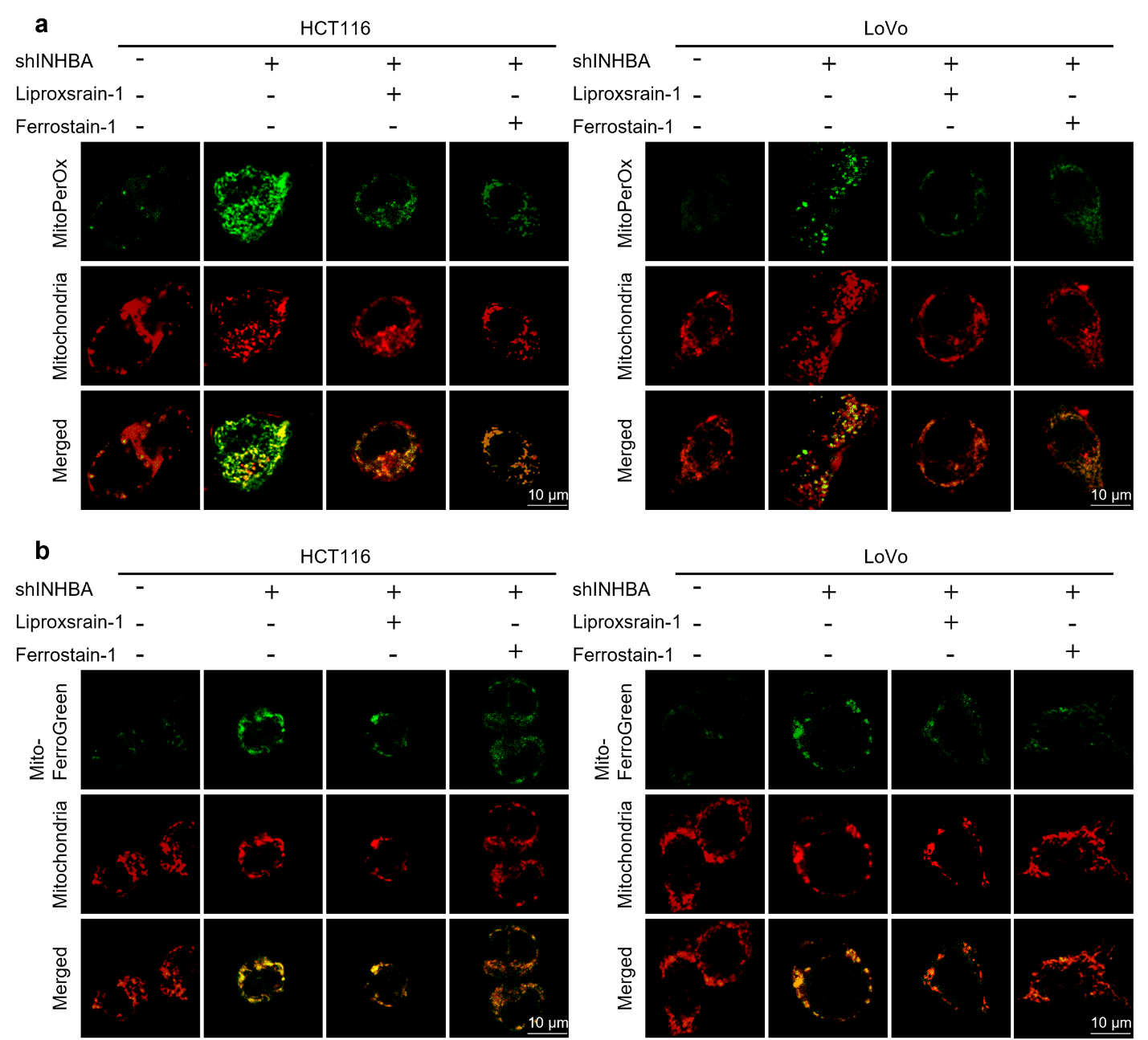


Supplementary Fig. 36. INHBA knockdown–induced mitochondrial lipid peroxidation and Fe²⁺ overload are rescued by ferroptosis inhibitors Liproxstatin-1 and Ferrostatin-1.

(a) INHBA was knocked down in human CRC cells in the presence of the ferroptosis inhibitor Liproxstatin-1 (MCE, USA; 1 μM for HCT116 cells, 10 μM for LoVo cells) or Ferrostatin-1 (MCE, USA; 0.1 μM for HCT116 cells, 1 μM for LoVo cells). Mitochondrial inner-membrane lipid peroxidation was then assessed with MitoPerOx. (b) INHBA was knocked down in human CRC cells in the presence of the ferroptosis inhibitor Liproxstatin-1 (MCE, USA; 1 μM for HCT116 cells, 10 μM for LoVo cells) or Ferrostatin-1 (MCE, USA; 0.1 μM for HCT116 cells, 1 μM for LoVo cells). Mitochondrial Fe²⁺ levels were monitored using Mito-FerroGreen.


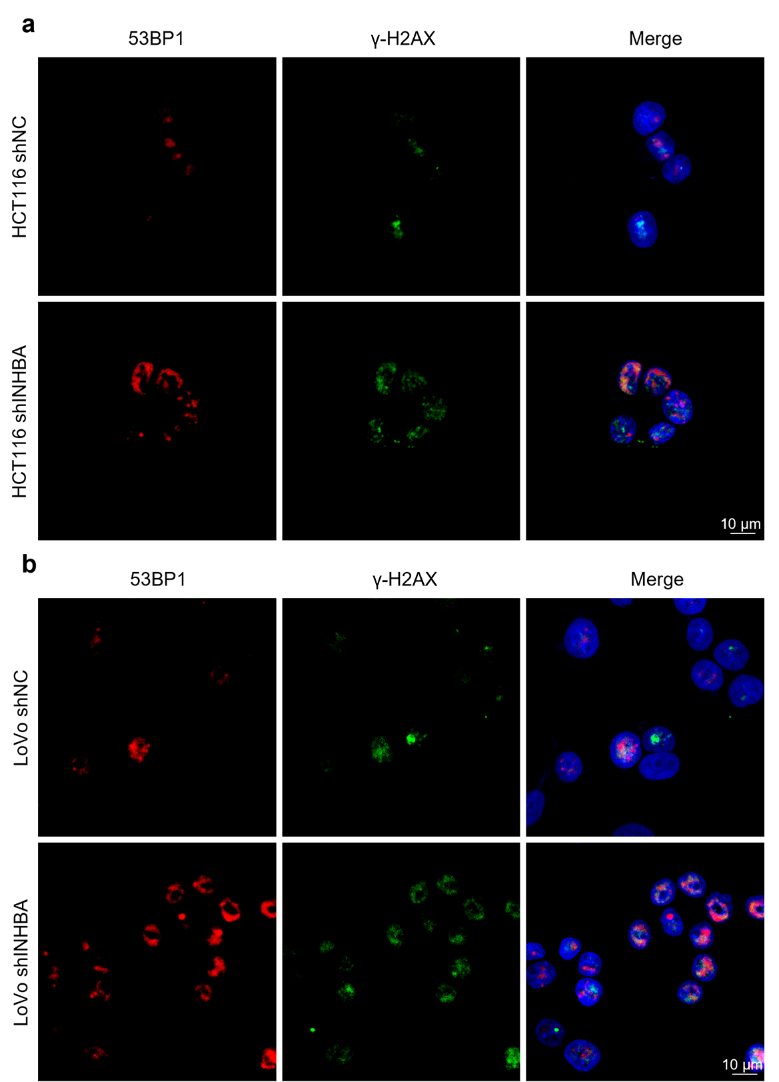


Supplementary Fig. 37. Immunofluorescence assay for detection of γ-H2AX/53BP1 co-localized DNA damage foci in INHBA-knockdown CRC cells.

Confocal immunofluorescence analysis of γ-H2AX/53BP1 co-localized DNA damage foci in INHBA-knockdown (a) HCT116 and (b) LoVo CRC cells using anti-γ-H2AX and anti-53BP1 antibodies.


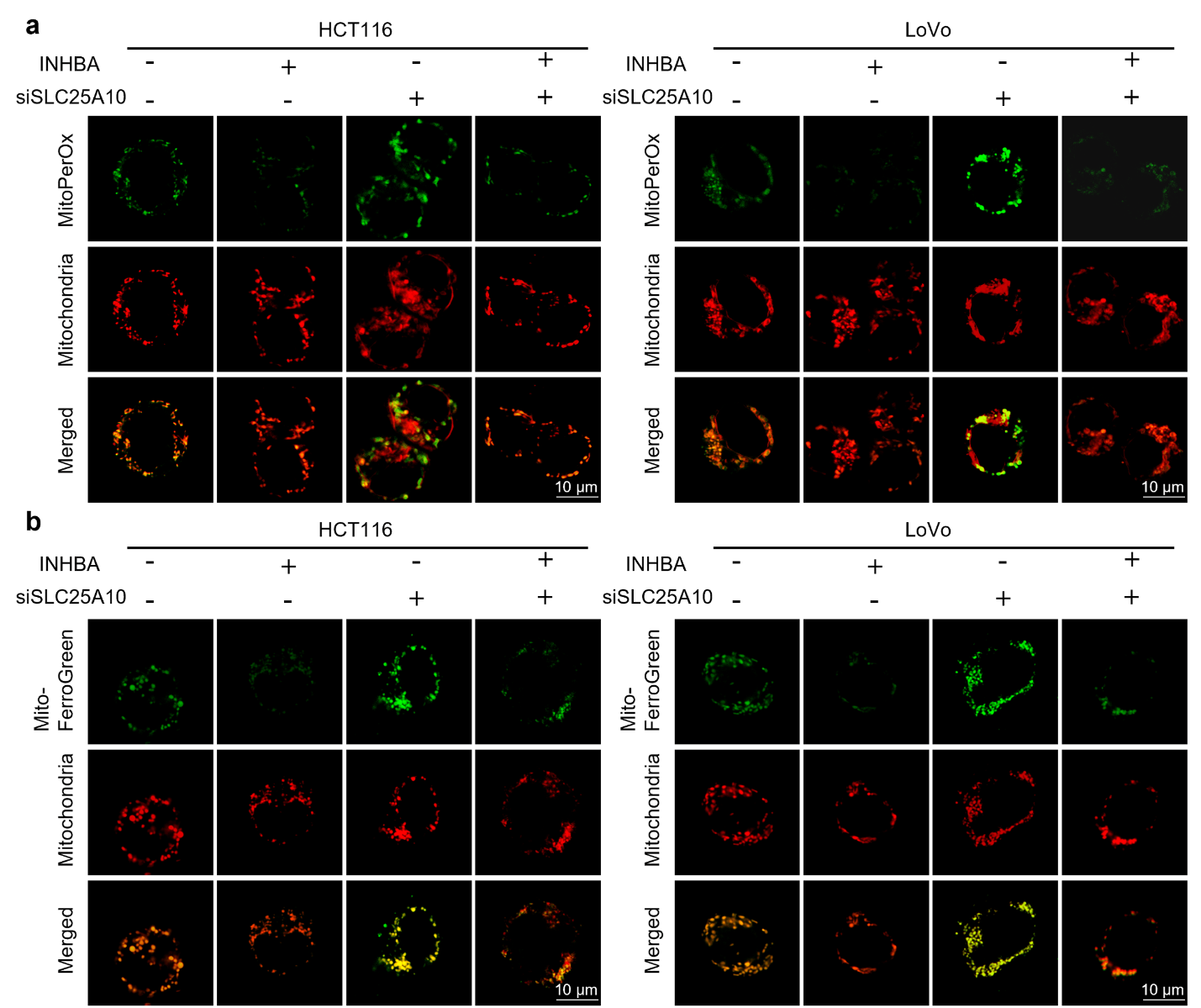


Supplementary Fig. 38. Knockdown of SLC25A10 partially reverses the decreased levels of lipid peroxidation and ferrous ions in CRC cells caused by INHBA overexpression.

In human CRC cells with INHBA overexpression and SLC25A10 knockdown: (a) Detection of lipid peroxidation levels: Lipid peroxidation levels in the mitochondrial inner membrane were detected using MitoPerOx. (b) Detection of ferrous ion changes: Changes in ferrous ions in mitochondria were detected using Mito-FerroGreen.


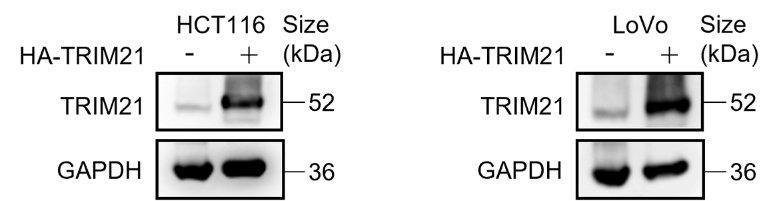


Supplementary Fig. 39. Western Blot experiments demonstrate the successful construction of CRC cell models with HA-TRIM21 overexpression.


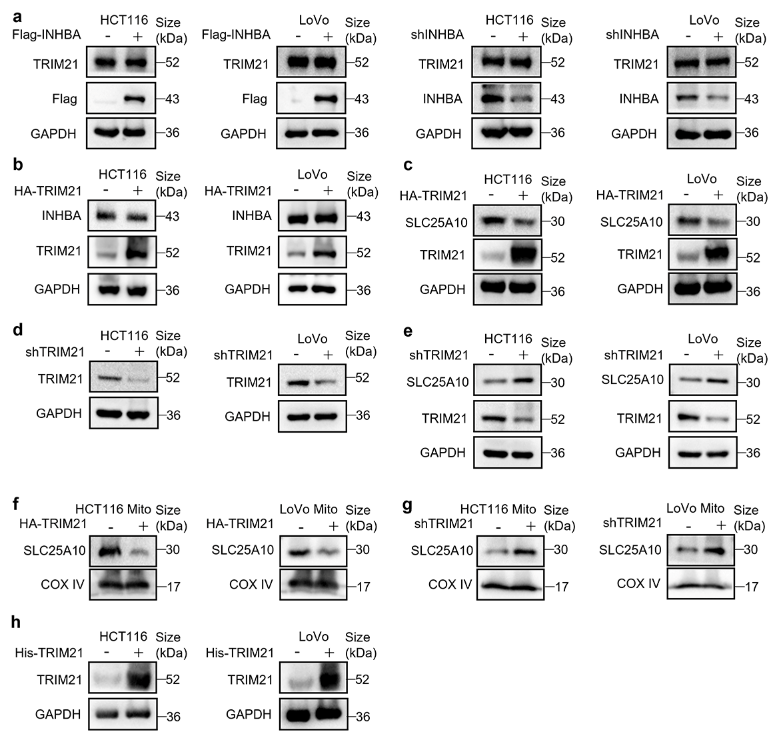


Supplementary Fig. 40. Regulatory relationships between INHBA, TRIM21, and SLC25A10 proteins.

(a) Effect of INHBA on TRIM21 protein levels: Overexpression or knockdown of INHBA in CRC cells, followed by detection of TRIM21 protein levels using Western Blot. (b) Effect of TRIM21 on INHBA protein levels: Overexpression of TRIM21 in CRC cells, followed by detection of INHBA protein levels using Western Blot. (c) Effect of TRIM21 on SLC25A10 protein levels: Overexpression of TRIM21 in CRC cells, followed by detection of SLC25A10 protein levels using Western Blot. (d) Western blotting confirmed efficient knockdown of TRIM21 in CRC cells. (e) Effect of TRIM21 knockdown on SLC25A10 protein levels. (f) Effect of TRIM21 overexpression on mitochondrial SLC25A10 protein levels: After overexpressing TRIM21 in CRC cells, mitochondria were isolated, and SLC25A10 protein levels in mitochondria were detected using Western Blot. (g) Effect of TRIM21 knockdown on mitochondrial SLC25A10 protein levels: After knocking down TRIM21 in CRC cells, mitochondria were isolated, and SLC25A10 protein levels in mitochondria were detected using Western Blot. (h) Validation of His-TRIM21 overexpression model: Confirmation of successful construction of CRC cell models with His-TRIM21 overexpression using Western Blot.


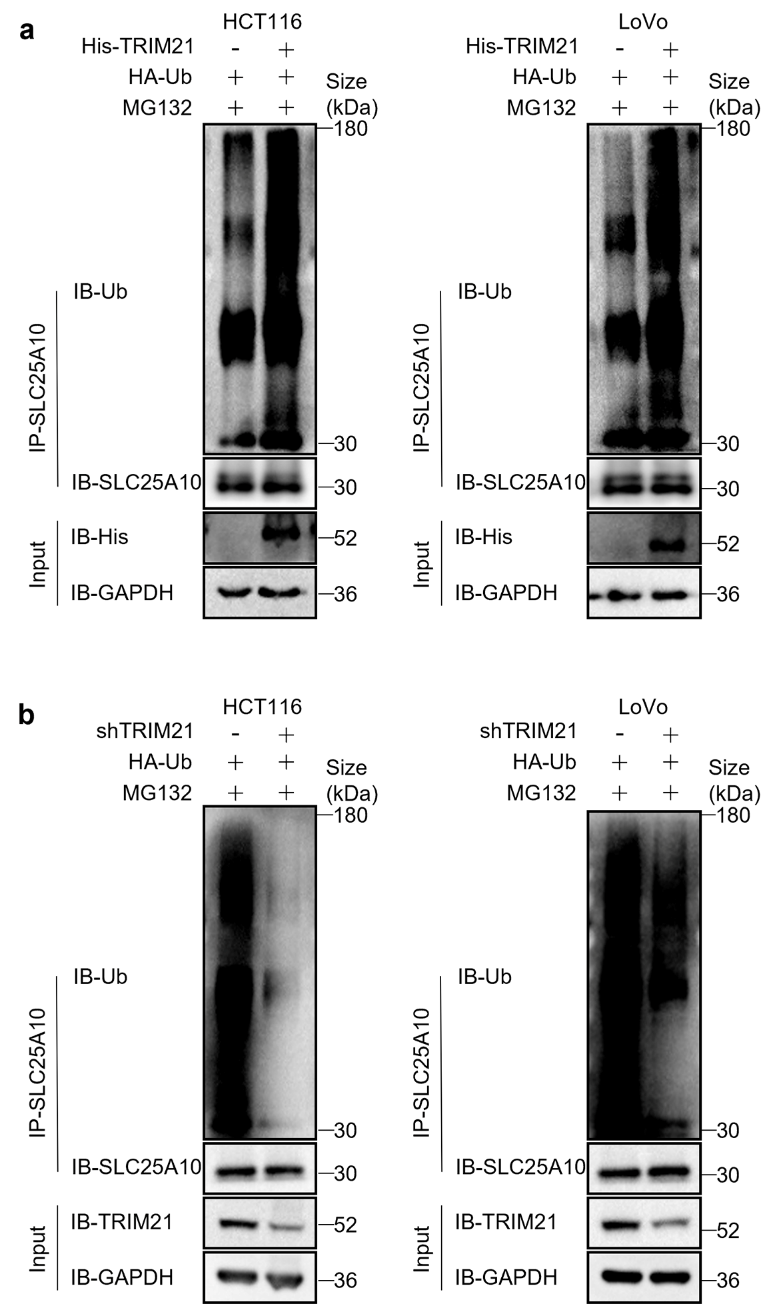


Supplementary Fig. 41. TRIM21 promotes the ubiquitination of SLC25A10 protein.

(a) In CRC cells, His-NC or His-TRIM21 and HA-Ub expression vectors were co-transfected, respectively. After 48 hours, cells were treated with MG132 for 4 hours. IP experiments were performed using anti-SLC25A10, and the ubiquitination level of SLC25A10 was detected using anti-Ub. (b) In CRC cells, shNC or shTRIM21 and HA-Ub expression vectors were co-transfected, respectively. After 48 hours, cells were treated with MG132 for 4 hours. IP experiments were performed using anti-SLC25A10, and the ubiquitination level of SLC25A10 was detected using anti-Ub.


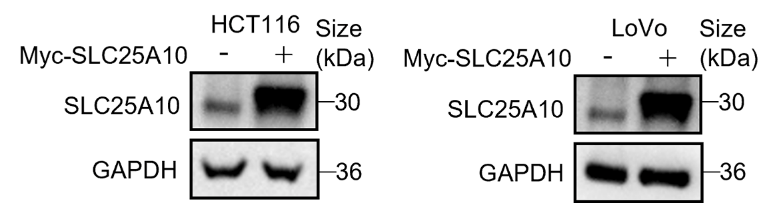


Supplementary Fig. 42. Western Blot experiments demonstrate the successful construction of CRC cell models with Myc-SLC25A10 overexpression.


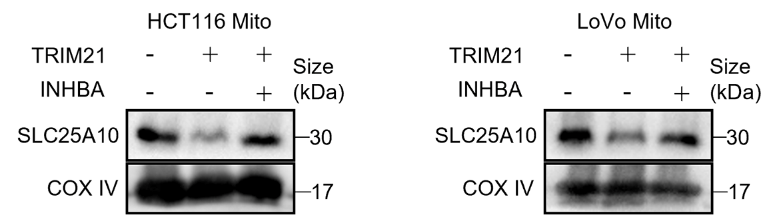


Supplementary Fig. 43. Western Blot experiments demonstrate that INHBA inhibits TRIM21-mediated ubiquitination and degradation of mitochondrial SLC25A10 protein.

In CRC cells, TRIM21 was overexpressed along with INHBA. Mitochondria were isolated using a mitochondrial separation kit, and the effects on mitochondrial SLC25A10 protein levels were detected by Western Blot.


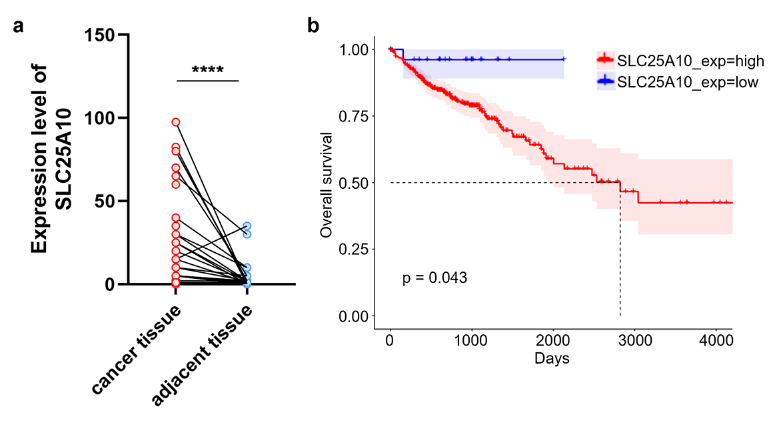


Supplementary Fig. 44. SLC25A10 is up-regulated in CRC and predicts poor prognosis.

(a) SLC25A10 protein expression is significantly higher in CRC tissues than in matched adjacent normal tissues. (b) Kaplan–Meier analysis of TCGA-COAD data shows that high SLC25A10 mRNA expression is associated with shorter OS.

Supplementary Table 1. Differential expression of INHBA in Colon cancer and adjacent tissues.

|  | n | INHBA expression | | Chi-square  Value | p value |
| --- | --- | --- | --- | --- | --- |
|  |  | High(%) | Low(%) |  |  |
| Colon cancer | 96 | 60 | 36 | 32.288 | <0.001 |
| Adjacent tissues | 74 | 14 | 60 |  |  |

* Statistically significant(p<0.05)

Supplementary Table 2. Correlation between INHBA expression and clinicopathological characteristics.

|  | variables | INHBA expression | | total | χ2 | p value |
| --- | --- | --- | --- | --- | --- | --- |
|  |  | low | high |  |  |  |
| Age (year) |  |  |  |  | 0.109 | 0.741 |
|  | <65 | 12 | 22 | 34 |  |  |
|  | ＞=65 | 24 | 38 | 62 |  |  |
| Sex |  |  |  |  | 2.504 | 0.114 |
|  | Female | 15 | 35 | 50 |  |  |
|  | male | 21 | 25 | 46 |  |  |
| Grade |  |  |  |  | 1.160 | 0.282 |
|  | I-II | 31 | 48 | 79 |  |  |
|  | III-IV | 4 | 12 | 16 |  |  |
| T stage |  |  |  |  | 6.759 | 0.034 |
|  | I-II | 5 | 1 | 6 |  |  |
|  | III | 27 | 45 | 72 |  |  |
|  | IV | 4 | 13 | 17 |  |  |
| N stage |  |  |  |  | 0.487 | 0.485 |
|  | N0 | 23 | 34 | 57 |  |  |
|  | N1- N2 | 13 | 26 | 39 |  |  |
| TNM stage |  |  |  |  | 0.487 | 0.485 |
|  | I-II | 23 | 34 | 57 |  |  |
|  | III- IV | 13 | 26 | 39 |  |  |

* Statistically significant(p<0.05)

Supplementary Table 3. Univariate and multivariate analyses of the factors correlated with overall survival in colon cancer patients.

Variables in the Equation

| Variables | Univariate analysis | | |  | Multivariate analysis | | |  |
| --- | --- | --- | --- | --- | --- | --- | --- | --- |
|  | HR | 95%CI | p value |  | HR | 95%CI | p value |  |
| Expression | 3.228 | 1.520-6.857 | 0.002 |  | 3.058 | 1.426-6.559 | 0.004 |  |
| Sex | 0.695 | 0.371-1.302 | 0.256 |  |  |  |  |  |
| Grade | 2.704 | 1.281-5.708 | 0.009 |  | 3.000 | 1.371-6.564 | 0.006 |  |
| Age | 1.631 | 0.814-3.266 | 0.167 |  |  |  |  |  |
| T stage | 1.612 | 0.864-3.009 | 0.133 |  |  |  |  |  |
| N stage | 2.562 | 1.374-4.779 | 0.003 |  | 2.391 | 1.251-4.567 | 0.008 |  |
| TNM stage | 2.562 | 1.374-4.779 | 0.003 |  |  |  |  |  |

* Statistically significant(p<0.05)

Supplementary Table 4. INHBA interactome in HCT116 cells (Co-IP/LC-MS).

| Accession | Protein names | Gene names | MW [kDa] | Protein score | Sequence coverage (%) | Unique Peptides | Peptides | PSMs | Abundances |
| --- | --- | --- | --- | --- | --- | --- | --- | --- | --- |
| Q13885 | Tubulin beta-2A chain | TUBB2A | 49.9 | 1330 | 48 | 1 | 17 | 33 | 5.11e+06 |
| Q9BQE3 | Tubulin alpha-1C chain | TUBA1C | 49.9 | 1186 | 52 | 1 | 17 | 29 | 2.08e+08 |
| P68366 | Tubulin alpha-4A chain | TUBA4A | 49.9 | 1185 | 46 | 2 | 15 | 28 | 1.17e+08 |
| P19474 | E3 ubiquitin-protein ligase TRIM21 | TRIM21 | 54.1 | 842 | 37 | 18 | 18 | 27 | 1.28e+09 |
| Q9NYF8 | Bcl-2-associated transcription factor 1 | BCLAF1 | 106.1 | 798 | 22 | 16 | 17 | 25 | 7.60e+08 |
| Q15942 | Zyxin | ZYX | 61.2 | 676 | 40 | 15 | 15 | 18 | 3.87e+08 |
| Q92804 | TATA-binding protein-associated factor 2N | TAF15 | 61.8 | 547 | 20 | 7 | 9 | 15 | 1.83e+09 |
| Q14247 | Src substrate cortactin | CTTN | 61.5 | 509 | 24 | 10 | 10 | 11 | 2.33e+08 |
| Q15424 | Scaffold attachment factor B1 | SAFB | 102.6 | 440 | 18 | 12 | 12 | 13 | 2.89e+08 |
| P55795 | Heterogeneous nuclear ribonucleoprotein H2 | HNRNPH2 | 49.2 | 415 | 21 | 1 | 8 | 10 | 3.40e+06 |
| Q96QC0 | Serine/threonine-protein phosphatase 1 regulatory subunit 10 | PPP1R10 | 99 | 405 | 16 | 12 | 12 | 13 | 1.20e+08 |
| Q562R1 | Beta-actin-like protein 2 | ACTBL2 | 42 | 379 | 18 | 1 | 6 | 16 | 1.06e+07 |
| Q16630 | Cleavage and polyadenylation specificity factor subunit 6 | CPSF6 | 59.2 | 311 | 12 | 4 | 4 | 6 | 5.09e+07 |
| P16989 | Y-box-binding protein 3 | YBX3 | 40.1 | 291 | 24 | 3 | 5 | 6 | 3.49e+07 |
| Q9UKV8 | Protein argonaute-2 | AGO2 | 97.1 | 273 | 9 | 7 | 7 | 9 | 4.65e+07 |
| P36873 | Serine/threonine-protein phosphatase PP1-gamma catalytic subunit | PPP1CC | 37 | 264 | 21 | 2 | 6 | 7 | 1.37e+08 |
| Q9NVI7 | ATPase family AAA domain-containing protein 3A | ATAD3A | 71.3 | 256 | 17 | 10 | 10 | 10 | 1.37e+08 |
| Q8WUA2 | Peptidyl-prolyl cis-trans isomerase-like 4 | PPIL4 | 57.2 | 254 | 14 | 5 | 5 | 5 | 4.09e+07 |
| P78332 | RNA-binding protein 6 | RBM6 | 128.6 | 237 | 6 | 5 | 6 | 7 | 5.30e+07 |
| P62136 | Serine/threonine-protein phosphatase PP1-alpha catalytic subunit | PPP1CA | 37.5 | 236 | 18 | 1 | 5 | 6 | 1.07e+07 |
| O43823 | A-kinase anchor protein 8 | AKAP8 | 76.1 | 223 | 8 | 4 | 4 | 5 | 3.43e+07 |
| A6NHL2 | Tubulin alpha chain-like 3 | TUBAL3 | 49.9 | 209 | 7 | 1 | 3 | 5 |  |
| P15104 | Glutamine synthetase | GLUL | 42 | 208 | 14 | 4 | 4 | 5 | 4.36e+07 |
| P98175 | RNA-binding protein 10 | RBM10 | 103.5 | 183 | 6 | 4 | 5 | 5 | 1.96e+07 |
| O95218 | Zinc finger Ran-binding domain-containing protein 2 | ZRANB2 | 37.4 | 174 | 22 | 6 | 6 | 6 | 1.01e+08 |
| O14639 | Actin-binding LIM protein 1 | ABLIM1 | 87.6 | 174 | 7 | 4 | 4 | 4 | 4.58e+07 |
| P62995 | Transformer-2 protein homolog beta | TRA2B | 33.6 | 172 | 15 | 3 | 3 | 3 | 4.06e+07 |
| O15372 | Eukaryotic translation initiation factor 3 subunit H | EIF3H | 39.9 | 172 | 26 | 6 | 6 | 6 | 8.97e+07 |
| O14744 | Protein arginine N-methyltransferase 5 | PRMT5 | 72.6 | 165 | 14 | 8 | 8 | 8 | 9.51e+07 |
| Q10570 | Cleavage and polyadenylation specificity factor subunit 1 | CPSF1 | 160.8 | 163 | 3 | 4 | 4 | 4 | 1.00e+07 |
| P31431 | Syndecan-4 | SDC4 | 21.6 | 160 | 14 | 3 | 3 | 3 | 2.77e+07 |
| Q9UBQ5 | Eukaryotic translation initiation factor 3 subunit K | EIF3K | 25 | 160 | 28 | 5 | 5 | 5 | 8.97e+08 |
| Q13243 | Serine/arginine-rich splicing factor 5 | SRSF5 | 31.2 | 159 | 12 | 2 | 3 | 4 | 2.41e+07 |
| Q06787 | Fragile X messenger ribonucleoprotein 1 | FMR1 | 71.1 | 157 | 2 | 1 | 1 | 2 | 8.41e+06 |
| Q14192 | Four and a half LIM domains protein 2 | FHL2 | 32.2 | 143 | 18 | 4 | 4 | 5 | 4.30e+07 |
| Q15654 | Thyroid receptor-interacting protein 6 | TRIP6 | 50.3 | 143 | 13 | 4 | 4 | 5 | 4.29e+07 |
| P52292 | Importin subunit alpha-1 | KPNA2 | 57.8 | 141 | 3 | 1 | 1 | 2 | 2.15e+06 |
| Q96C00 | Zinc finger and BTB domain-containing protein 9 | ZBTB9 | 50.6 | 141 | 17 | 6 | 6 | 6 | 7.73e+07 |
| Q92504 | Zinc transporter SLC39A7 | SLC39A7 | 50.1 | 140 | 8 | 3 | 3 | 5 | 9.53e+07 |
| A0A075B6R9 | Probable non-functional immunoglobulin kappa variable 2D-24 | IGKV2D-24 | 13.1 | 135 | 13 | 1 | 2 | 4 | 7.70e+09 |
| Q86V81 | THO complex subunit 4 | ALYREF | 26.9 | 132 | 12 | 2 | 2 | 2 | 1.89e+07 |
| O43852 | Calumenin | CALU | 37.1 | 126 | 12 | 3 | 3 | 4 | 2.28e+07 |
| P49761 | Dual specificity protein kinase CLK3 | CLK3 | 73.5 | 125 | 7 | 4 | 4 | 5 | 5.31e+07 |
| Q9BXP5 | Serrate RNA effector molecule homolog | SRRT | 100.6 | 117 | 5 | 3 | 3 | 3 | 4.34e+07 |
| Q99439 | Calponin-2 | CNN2 | 33.7 | 111 | 17 | 4 | 4 | 4 | 4.81e+07 |
| O15427 | Monocarboxylate transporter 4 | SLC16A3 | 49.4 | 110 | 7 | 3 | 3 | 3 | 2.96e+07 |
| O15042 | U2 snRNP-associated SURP motif-containing protein | U2SURP | 118.2 | 109 | 4 | 4 | 4 | 4 | 3.96e+07 |
| Q09161 | Nuclear cap-binding protein subunit 1 | NCBP1 | 91.8 | 108 | 4 | 3 | 3 | 3 | 9.58e+06 |
| P47895 | Retinaldehyde dehydrogenase 3 | ALDH1A3 | 56.1 | 105 | 13 | 4 | 4 | 4 | 2.11e+07 |
| Q08170 | Serine/arginine-rich splicing factor 4 | SRSF4 | 56.6 | 104 | 6 | 1 | 3 | 4 | 2.23e+07 |
| Q13501 | Sequestosome-1 | SQSTM1 | 47.7 | 103 | 8 | 3 | 3 | 3 | 2.46e+07 |
| O95429 | BAG family molecular chaperone regulator 4 | BAG4 | 49.6 | 102 | 6 | 2 | 2 | 2 | 1.27e+07 |
| Q9UGU0 | Transcription factor 20 | TCF20 | 211.6 | 102 | 3 | 4 | 4 | 4 | 2.01e+07 |
| Q12797 | Aspartyl/asparaginyl beta-hydroxylase | ASPH | 85.8 | 99 | 3 | 2 | 2 | 2 | 1.48e+07 |
| Q6Y7W6 | GRB10-interacting GYF protein 2 | GIGYF2 | 150 | 96 | 3 | 3 | 3 | 3 | 1.29e+07 |
| Q16629 | Serine/arginine-rich splicing factor 7 | SRSF7 | 27.4 | 95 | 17 | 3 | 4 | 4 | 9.86e+07 |
| O60884 | DnaJ homolog subfamily A member 2 | DNAJA2 | 45.7 | 92 | 9 | 3 | 3 | 3 | 2.19e+07 |
| Q9BZR9 | E3 ubiquitin-protein ligase TRIM8 | TRIM8 | 61.4 | 90 | 4 | 2 | 2 | 2 | 1.06e+07 |
| Q92945 | Far upstream element-binding protein 2 | KHSRP | 73.1 | 89 | 4 | 2 | 2 | 2 | 1.33e+07 |
| Q14257 | Reticulocalbin-2 | RCN2 | 36.9 | 88 | 7 | 2 | 2 | 2 | 1.90e+07 |
| Q9BQA1 | Methylosome protein WDR77 | WDR77 | 36.7 | 88 | 4 | 1 | 1 | 2 | 9.76e+05 |
| P04181 | Ornithine aminotransferase, mitochondrial | OAT | 48.5 | 84 | 4 | 2 | 2 | 2 | 1.56e+07 |
| Q8NEY8 | Periphilin-1 | PPHLN1 | 52.7 | 83 | 10 | 5 | 5 | 5 | 7.31e+07 |
| P38159 | RNA-binding motif protein, X chromosome | RBMX | 42.3 | 79 | 7 | 3 | 3 | 3 | 4.19e+07 |
| P50993 | Sodium/potassium-transporting ATPase subunit alpha-2 | ATP1A2 | 112.2 | 78 | 2 | 2 | 2 | 2 | 1.45e+07 |
| Q86VP6 | Cullin-associated NEDD8-dissociated protein 1 | CAND1 | 136.3 | 77 | 1 | 1 | 1 | 1 | 2.20e+06 |
| Q6UN15 | Pre-mRNA 3'-end-processing factor FIP1 | FIP1L1 | 66.5 | 77 | 2 | 1 | 1 | 1 | 1.42e+07 |
| P25205 | DNA replication licensing factor MCM3 | MCM3 | 90.9 | 76 | 3 | 3 | 3 | 3 | 1.19e+07 |
| P17480 | Nucleolar transcription factor 1 | UBTF | 89.4 | 75 | 5 | 3 | 3 | 3 | 1.54e+07 |
| Q92615 | La-related protein 4B | LARP4B | 80.5 | 75 | 4 | 2 | 3 | 3 | 1.53e+07 |
| Q04637 | Eukaryotic translation initiation factor 4 gamma 1 | EIF4G1 | 175.4 | 75 | 2 | 4 | 4 | 4 | 1.37e+07 |
| Q99417 | c-Myc-binding protein | MYCBP | 12 | 74 | 35 | 3 | 3 | 3 | 1.85e+07 |
| P07910 | Heterogeneous nuclear ribonucleoproteins C1/C2 | HNRNPC | 33.7 | 73 | 9 | 2 | 2 | 2 | 1.07e+07 |
| Q9Y5Y6 | Suppressor of tumorigenicity 14 protein | ST14 | 94.7 | 73 | 3 | 2 | 2 | 2 | 1.07e+07 |
| Q8IWX8 | Calcium homeostasis endoplasmic reticulum protein | CHERP | 103.6 | 72 | 3 | 2 | 2 | 3 | 2.42e+07 |
| Q15365 | Poly(rC)-binding protein 1 | PCBP1 | 37.5 | 71 | 6 | 1 | 2 | 2 | 2.16e+07 |
| P22695 | Cytochrome b-c1 complex subunit 2, mitochondrial | UQCRC2 | 48.4 | 71 | 4 | 1 | 1 | 1 | 2.51e+06 |
| O95633 | Follistatin-related protein 3 | FSTL3 | 27.6 | 70 | 13 | 2 | 2 | 2 | 6.60e+06 |
| Q8NEJ9 | Neuroguidin | NGDN | 35.9 | 69 | 6 | 1 | 1 | 1 |  |
| Q9P2E9 | Ribosome-binding protein 1 | RRBP1 | 152.4 | 68 | 2 | 2 | 2 | 2 | 4.68e+06 |
| Q9BRL6 | Serine/arginine-rich splicing factor 8 | SRSF8 | 32.3 | 68 | 2 | 1 | 1 | 1 | 1.81e+07 |
| Q9UBX3 | Mitochondrial dicarboxylate carrier | SLC25A10 | 31.3 | 68 | 11 | 2 | 2 | 2 | 8.10e+06 |
| P22830 | Ferrochelatase, mitochondrial | FECH | 47.8 | 67 | 5 | 1 | 1 | 1 |  |
| P10155 | RNA-binding protein RO60 | RO60 | 60.6 | 67 | 2 | 1 | 1 | 1 | 2.13e+06 |
| Q8WU68 | Splicing factor U2AF 26 kDa subunit | U2AF1L4 | 25.7 | 65 | 5 | 1 | 1 | 1 |  |
| Q9C037 | E3 ubiquitin-protein ligase TRIM4 | TRIM4 | 57.4 | 65 | 7 | 3 | 3 | 3 | 6.15e+07 |
| P35568 | Insulin receptor substrate 1 | IRS1 | 131.5 | 65 | 2 | 2 | 2 | 2 | 1.32e+07 |
| Q8WXF0 | Serine/arginine-rich splicing factor 12 | SRSF12 | 30.5 | 64 | 4 | 1 | 1 | 1 | 1.27e+07 |
| Q9H307 | Pinin | PNN | 81.6 | 64 | 3 | 2 | 2 | 2 | 6.05e+06 |
| Q9NZ01 | Very-long-chain enoyl-CoA reductase | TECR | 36 | 64 | 10 | 3 | 3 | 3 | 2.74e+07 |
| Q13200 | 26S proteasome non-ATPase regulatory subunit 2 | PSMD2 | 100.1 | 63 | 3 | 2 | 2 | 2 | 8.00e+06 |
| P26368 | Splicing factor U2AF 65 kDa subunit | U2AF2 | 53.5 | 63 | 9 | 2 | 2 | 2 | 6.56e+06 |
| Q92743 | Serine protease HTRA1 | HTRA1 | 51.3 | 63 | 4 | 2 | 2 | 2 | 1.34e+07 |
| P28290 | Protein ITPRID2 | ITPRID2 | 138.3 | 62 | 2 | 2 | 2 | 2 | 3.88e+06 |
| Q68E01 | Integrator complex subunit 3 | INTS3 | 118 | 62 | 2 | 1 | 1 | 1 |  |
| Q9Y678 | Coatomer subunit gamma-1 | COPG1 | 97.7 | 61 | 2 | 2 | 2 | 2 | 5.21e+06 |
| Q8TCF1 | AN1-type zinc finger protein 1 | ZFAND1 | 30.8 | 61 | 12 | 3 | 3 | 3 | 2.11e+07 |
| Q13595 | Transformer-2 protein homolog alpha | TRA2A | 32.7 | 61 | 6 | 2 | 2 | 2 | 1.17e+07 |
| Q02978 | Mitochondrial 2-oxoglutarate/malate carrier protein | SLC25A11 | 34 | 58 | 8 | 2 | 2 | 2 | 1.23e+07 |
| P07858 | Cathepsin B | CTSB | 37.8 | 58 | 6 | 2 | 2 | 2 | 1.46e+07 |
| Q9ULC5 | Long-chain-fatty-acid--CoA ligase 5 | ACSL5 | 75.9 | 57 | 2 | 1 | 1 | 1 | 3.13e+06 |
| Q7Z2W4 | Zinc finger CCCH-type antiviral protein 1 | ZC3HAV1 | 101.4 | 57 | 2 | 2 | 2 | 2 | 1.61e+07 |
| Q9UBR2 | Cathepsin Z | CTSZ | 33.8 | 57 | 3 | 1 | 1 | 1 | 2.03e+07 |
| O60762 | Dolichol-phosphate mannosyltransferase subunit 1 | DPM1 | 29.6 | 56 | 8 | 2 | 2 | 2 | 1.05e+07 |
| Q86UK7 | E3 ubiquitin-protein ligase ZNF598 | ZNF598 | 98.6 | 56 | 2 | 1 | 1 | 1 | 2.54e+06 |
| Q9Y295 | Developmentally-regulated GTP-binding protein 1 | DRG1 | 40.5 | 55 | 4 | 1 | 1 | 1 | 3.02e+06 |
| Q96HC4 | PDZ and LIM domain protein 5 | PDLIM5 | 63.9 | 55 | 6 | 3 | 3 | 3 | 1.54e+07 |
| P06493 | Cyclin-dependent kinase 1 | CDK1 | 34.1 | 55 | 6 | 2 | 2 | 2 | 2.44e+07 |
| Q15393 | Splicing factor 3B subunit 3 | SF3B3 | 135.5 | 55 | 1 | 1 | 1 | 1 | 4.17e+06 |
| P49327 | Fatty acid synthase | FASN | 273.3 | 55 | 2 | 3 | 3 | 3 | 8.82e+06 |
| P30049 | ATP synthase subunit delta, mitochondrial | ATP5F1D | 17.5 | 54 | 8 | 1 | 1 | 1 | 3.70e+06 |
| Q9NX58 | Cell growth-regulating nucleolar protein | LYAR | 43.6 | 54 | 9 | 2 | 2 | 4 | 3.28e+07 |
| Q9H329 | Band 4.1-like protein 4B | EPB41L4B | 99.7 | 53 | 1 | 1 | 1 | 1 | 1.35e+06 |
| P36542 | ATP synthase subunit gamma, mitochondrial | ATP5F1C | 33 | 53 | 7 | 2 | 2 | 2 | 4.08e+06 |
| P60468 | Protein transport protein Sec61 subunit beta | SEC61B | 10 | 53 | 16 | 1 | 1 | 1 | 1.03e+07 |
| P40938 | Replication factor C subunit 3 | RFC3 | 40.5 | 53 | 5 | 1 | 1 | 1 | 3.00e+06 |
| Q03405 | Urokinase plasminogen activator surface receptor | PLAUR | 37 | 53 | 11 | 2 | 2 | 2 | 1.34e+07 |
| P13569 | Cystic fibrosis transmembrane conductance regulator | CFTR | 168 | 52 | 0 | 1 | 1 | 2 | 1.09e+07 |
| Q9NRR4 | Ribonuclease 3 | DROSHA | 159.2 | 52 | 1 | 1 | 1 | 1 | 2.78e+06 |
| O95819 | Mitogen-activated protein kinase kinase kinase kinase 4 | MAP4K4 | 142 | 52 | 1 | 2 | 2 | 2 | 6.29e+07 |
| O75533 | Splicing factor 3B subunit 1 | SF3B1 | 145.7 | 51 | 2 | 2 | 2 | 2 | 7.22e+06 |
| O14979 | Heterogeneous nuclear ribonucleoprotein D-like | HNRNPDL | 46.4 | 50 | 4 | 1 | 2 | 2 | 4.35e+06 |
| P07205 | Phosphoglycerate kinase 2 | PGK2 | 44.8 | 50 | 5 | 2 | 2 | 2 | 1.00e+07 |
| Q9H9B4 | Sideroflexin-1 | SFXN1 | 35.6 | 49 | 4 | 1 | 1 | 1 |  |
| P55209 | Nucleosome assembly protein 1-like 1 | NAP1L1 | 45.3 | 48 | 5 | 2 | 2 | 2 | 7.77e+06 |
| P08574 | Cytochrome c1, heme protein, mitochondrial | CYC1 | 35.4 | 48 | 4 | 1 | 1 | 1 | 7.16e+06 |
| P38935 | DNA-binding protein SMUBP-2 | IGHMBP2 | 109.1 | 47 | 1 | 1 | 1 | 1 | 6.95e+07 |
| P40937 | Replication factor C subunit 5 | RFC5 | 38.5 | 47 | 9 | 2 | 2 | 2 | 7.11e+06 |
| Q7KZF4 | Staphylococcal nuclease domain-containing protein 1 | SND1 | 101.9 | 47 | 2 | 1 | 1 | 1 | 3.41e+06 |
| Q9NXR1 | Nuclear distribution protein nudE homolog 1 | NDE1 | 37.7 | 46 | 4 | 1 | 1 | 1 | 9.79e+05 |
| P53618 | Coatomer subunit beta | COPB1 | 107.1 | 46 | 1 | 1 | 1 | 1 | 1.80e+06 |
| Q9NQY0 | Bridging integrator 3 | BIN3 | 29.6 | 46 | 7 | 2 | 2 | 2 | 2.18e+07 |
| P62314 | Small nuclear ribonucleoprotein Sm D1 | SNRPD1 | 13.3 | 46 | 11 | 1 | 1 | 1 | 3.53e+06 |
| Q92522 | Histone H1.10 | H1-10 | 22.5 | 46 | 5 | 1 | 1 | 1 | 1.83e+07 |
| Q9UKA9 | Polypyrimidine tract-binding protein 2 | PTBP2 | 57.5 | 46 | 2 | 1 | 1 | 1 | 2.32e+07 |
| P35250 | Replication factor C subunit 2 | RFC2 | 39.1 | 45 | 5 | 2 | 2 | 2 | 9.04e+06 |
| O60841 | Eukaryotic translation initiation factor 5B | EIF5B | 138.7 | 45 | 1 | 1 | 1 | 1 | 2.38e+06 |
| Q16531 | DNA damage-binding protein 1 | DDB1 | 126.9 | 44 | 1 | 1 | 1 | 1 | 5.18e+06 |
| Q9UGI8 | Testin | TES | 48 | 44 | 3 | 1 | 1 | 1 | 4.60e+06 |
| Q15208 | Serine/threonine-protein kinase 38 | STK38 | 54.2 | 44 | 2 | 1 | 1 | 1 | 4.33e+06 |
| O75477 | Erlin-1 | ERLIN1 | 39.1 | 44 | 3 | 1 | 1 | 1 | 6.66e+06 |
| Q5HYM0 | Probable ribonuclease ZC3H12B | ZC3H12B | 94.1 | 43 | 2 | 2 | 2 | 2 | 5.46e+06 |
| Q6XZF7 | Dynamin-binding protein | DNMBP | 177.2 | 43 | 1 | 1 | 1 | 1 | 1.26e+06 |
| Q96IU4 | Putative protein-lysine deacylase ABHD14B | ABHD14B | 22.3 | 43 | 5 | 1 | 1 | 1 | 1.07e+06 |
| P06730 | Eukaryotic translation initiation factor 4E | EIF4E | 25.1 | 42 | 6 | 2 | 2 | 2 | 1.11e+07 |
| Q9H3P2 | Negative elongation factor A | NELFA | 57.2 | 42 | 2 | 1 | 1 | 1 |  |
| P25789 | Proteasome subunit alpha type-4 | PSMA4 | 29.5 | 42 | 4 | 1 | 1 | 1 | 2.53e+06 |
| P35052 | Glypican-1 | GPC1 | 61.6 | 42 | 5 | 2 | 2 | 2 | 1.22e+07 |
| P02743 | Serum amyloid P-component | APCS | 25.4 | 41 | 3 | 1 | 1 | 1 | 6.62e+06 |
| Q9UG63 | ATP-binding cassette sub-family F member 2 | ABCF2 | 71.2 | 41 | 2 | 1 | 1 | 1 | 2.75e+06 |
| Q13155 | Aminoacyl tRNA synthase complex-interacting multifunctional protein 2 | AIMP2 | 35.3 | 41 | 3 | 1 | 1 | 1 | 1.43e+06 |
| C9JLW8 | Mapk-regulated corepressor-interacting protein 1 | MCRIP1 | 10.9 | 41 | 11 | 1 | 1 | 1 | 4.77e+06 |
| Q8NAB2 | Kelch repeat and BTB domain-containing protein 3 | KBTBD3 | 69.8 | 41 | 1 | 1 | 1 | 1 | 7.27e+06 |
| P23284 | Peptidyl-prolyl cis-trans isomerase B | PPIB | 23.7 | 41 | 4 | 1 | 1 | 1 | 3.57e+06 |
| Q86Z14 | Beta-klotho | KLB | 119.7 | 41 | 1 | 1 | 1 | 2 | 1.37e+08 |
| Q6ZXV5 | Protein O-mannosyl-transferase TMTC3 | TMTC3 | 103.9 | 40 | 1 | 1 | 1 | 1 | 2.57e+06 |
| P43686 | 26S proteasome regulatory subunit 6B | PSMC4 | 47.3 | 40 | 6 | 2 | 2 | 2 | 1.49e+07 |
| O60573 | Eukaryotic translation initiation factor 4E type 2 | EIF4E2 | 28.3 | 40 | 3 | 1 | 1 | 1 | 3.14e+06 |
| Q7Z333 | Probable helicase senataxin | SETX | 302.7 | 40 | 0 | 1 | 1 | 1 | 4.30e+06 |
| Q96T51 | RUN and FYVE domain-containing protein 1 | RUFY1 | 79.8 | 39 | 3 | 2 | 2 | 2 | 1.61e+07 |
| P62841 | Small ribosomal subunit protein uS19 | RPS15 | 17 | 39 | 8 | 1 | 1 | 2 | 6.60e+07 |
| Q9UPQ9 | Trinucleotide repeat-containing gene 6B protein | TNRC6B | 193.9 | 39 | 1 | 1 | 1 | 1 | 7.93e+06 |
| Q96PX9 | Pleckstrin homology domain-containing family G member 4B | PLEKHG4B | 139.6 | 38 | 1 | 1 | 1 | 2 | 4.27e+06 |
| P46459 | Vesicle-fusing ATPase | NSF | 82.5 | 38 | 2 | 1 | 1 | 1 |  |
| P06396 | Gelsolin | GSN | 85.6 | 38 | 2 | 1 | 1 | 1 | 6.07e+06 |
| P06703 | Protein S100-A6 | S100A6 | 10.2 | 37 | 9 | 1 | 1 | 1 | 2.49e+07 |
| Q8NH93 | Olfactory receptor 1L3 | OR1L3 | 36.6 | 37 | 2 | 1 | 1 | 1 | 5.63e+06 |
| Q96SB3 | Neurabin-2 | PPP1R9B | 89.3 | 37 | 1 | 1 | 1 | 1 | 2.28e+06 |
| P16298 | Serine/threonine-protein phosphatase 2B catalytic subunit beta isoform | PPP3CB | 59 | 36 | 2 | 1 | 1 | 1 | 1.69e+07 |
| Q00341 | Vigilin | HDLBP | 141.4 | 36 | 1 | 1 | 1 | 1 |  |
| Q9C0J8 | pre-mRNA 3' end processing protein WDR33 | WDR33 | 145.8 | 36 | 0 | 1 | 1 | 1 | 3.49e+06 |
| Q9BQ24 | Zinc finger FYVE domain-containing protein 21 | ZFYVE21 | 26.5 | 35 | 3 | 1 | 1 | 1 | 3.74e+06 |
| P00390 | Glutathione reductase, mitochondrial | GSR | 56.2 | 35 | 2 | 1 | 1 | 1 | 4.45e+06 |
| P26639 | Threonine--tRNA ligase 1, cytoplasmic | TARS1 | 83.4 | 35 | 1 | 1 | 1 | 1 | 1.31e+06 |
| Q9BVP2 | Guanine nucleotide-binding protein-like 3 | GNL3 | 62 | 35 | 2 | 1 | 1 | 1 | 6.24e+06 |
| Q96DV4 | Large ribosomal subunit protein mL38 | MRPL38 | 44.6 | 35 | 2 | 1 | 1 | 1 | 2.02e+06 |
| Q14493 | Histone RNA hairpin-binding protein | SLBP | 31.3 | 35 | 4 | 1 | 1 | 1 | 1.05e+07 |
| Q96I24 | Far upstream element-binding protein 3 | FUBP3 | 61.6 | 35 | 1 | 1 | 1 | 1 | 5.79e+06 |
| P52434 | DNA-directed RNA polymerases I, II, and III subunit RPABC3 | POLR2H | 17.1 | 34 | 8 | 1 | 1 | 1 |  |
| Q8WYP3 | Ras and Rab interactor 2 | RIN2 | 100.1 | 34 | 1 | 1 | 1 | 1 | 2.40e+06 |
| P08621 | U1 small nuclear ribonucleoprotein 70 kDa | SNRNP70 | 51.5 | 34 | 3 | 1 | 1 | 1 | 6.55e+06 |
| Q9ULX6 | A-kinase anchor protein 8-like | AKAP8L | 71.6 | 34 | 2 | 1 | 1 | 1 | 1.93e+06 |
| P62318 | Small nuclear ribonucleoprotein Sm D3 | SNRPD3 | 13.9 | 34 | 7 | 1 | 1 | 1 | 3.98e+06 |
| Q14244 | Ensconsin | MAP7 | 84 | 33 | 1 | 1 | 1 | 1 |  |
| P49916 | DNA ligase 3 | LIG3 | 112.8 | 33 | 1 | 1 | 1 | 1 | 3.15e+06 |
| O14735 | CDP-diacylglycerol--inositol 3-phosphatidyltransferase | CDIPT | 23.5 | 33 | 6 | 1 | 1 | 1 | 2.26e+06 |
| Q13642 | Four and a half LIM domains protein 1 | FHL1 | 36.2 | 32 | 4 | 1 | 1 | 1 | 7.46e+06 |
| Q96T76 | MMS19 nucleotide excision repair protein homolog | MMS19 | 113.2 | 32 | 1 | 1 | 1 | 1 | 3.16e+06 |
| P02766 | Transthyretin | TTR | 15.9 | 32 | 4 | 1 | 1 | 1 | 2.26e+07 |
| Q8IYB3 | Serine/arginine repetitive matrix protein 1 | SRRM1 | 102.3 | 32 | 1 | 1 | 1 | 1 | 7.74e+06 |
| O15014 | Zinc finger protein 609 | ZNF609 | 151.1 | 32 | 1 | 1 | 1 | 1 | 4.45e+06 |
| P17980 | 26S proteasome regulatory subunit 6A | PSMC3 | 49.2 | 32 | 2 | 1 | 1 | 1 | 2.89e+06 |
| O00217 | NADH dehydrogenase [ubiquinone] iron-sulfur protein 8, mitochondrial | NDUFS8 | 23.7 | 31 | 4 | 1 | 1 | 1 | 1.67e+06 |
| Q8NE71 | ATP-binding cassette sub-family F member 1 | ABCF1 | 95.9 | 31 | 1 | 1 | 1 | 1 | 3.03e+06 |
| Q9Y580 | RNA-binding protein 7 | RBM7 | 30.5 | 31 | 7 | 1 | 1 | 1 | 1.89e+06 |
| O43837 | Isocitrate dehydrogenase [NAD] subunit beta, mitochondrial | IDH3B | 42.2 | 31 | 2 | 1 | 1 | 1 |  |
| Q6P1J9 | Parafibromin | CDC73 | 60.5 | 31 | 2 | 1 | 1 | 1 | 1.17e+06 |
| Q9H857 | 5'-nucleotidase domain-containing protein 2 | NT5DC2 | 60.7 | 30 | 2 | 1 | 1 | 1 | 1.24e+06 |
| Q96I25 | Splicing factor 45 | RBM17 | 44.9 | 30 | 2 | 1 | 1 | 1 | 9.46e+06 |
| O15234 | Protein CASC3 | CASC3 | 76.2 | 29 | 2 | 1 | 1 | 1 | 4.64e+06 |
| Q92552 | Small ribosomal subunit protein mS27 | MRPS27 | 47.6 | 29 | 3 | 1 | 1 | 1 | 2.71e+06 |
| P04183 | Thymidine kinase, cytosolic | TK1 | 25.5 | 29 | 8 | 1 | 1 | 1 | 3.23e+06 |
| P12259 | Coagulation factor V | F5 | 251.5 | 28 | 0 | 1 | 1 | 1 | 8.01e+06 |
| P22061 | Protein-L-isoaspartate(D-aspartate) O-methyltransferase | PCMT1 | 24.6 | 28 | 6 | 1 | 1 | 1 | 1.02e+07 |
| O15258 | Protein RER1 | RER1 | 22.9 | 28 | 7 | 1 | 1 | 1 | 3.74e+06 |
| Q86WI3 | Protein NLRC5 | NLRC5 | 204.5 | 28 | 0 | 1 | 1 | 1 | 7.39e+06 |
| P35998 | 26S proteasome regulatory subunit 7 | PSMC2 | 48.6 | 28 | 3 | 1 | 1 | 1 | 2.51e+06 |
| Q9P2J5 | Leucine--tRNA ligase, cytoplasmic | LARS1 | 134.4 | 28 | 1 | 1 | 1 | 1 | 1.65e+06 |
| Q07065 | Cytoskeleton-associated protein 4 | CKAP4 | 66 | 28 | 2 | 1 | 1 | 1 | 5.19e+06 |
| Q12789 | General transcription factor 3C polypeptide 1 | GTF3C1 | 238.7 | 27 | 0 | 1 | 1 | 1 | 5.00e+06 |
| Q9UMS4 | Pre-mRNA-processing factor 19 | PRPF19 | 55.1 | 27 | 2 | 1 | 1 | 1 | 5.45e+06 |
| Q9UDW1 | Cytochrome b-c1 complex subunit 9 | UQCR10 | 7.3 | 27 | 11 | 1 | 1 | 1 | 2.01e+06 |
| Q9P035 | Very-long-chain (3R)-3-hydroxyacyl-CoA dehydratase 3 | HACD3 | 43.1 | 27 | 3 | 1 | 1 | 1 | 2.69e+06 |
| Q7Z7A3 | Cytoplasmic tRNA 2-thiolation protein 1 | CTU1 | 36.4 | 26 | 4 | 1 | 1 | 1 | 5.50e+06 |
| P31689 | DnaJ homolog subfamily A member 1 | DNAJA1 | 44.8 | 26 | 3 | 1 | 1 | 1 | 2.65e+06 |
| P17028 | Zinc finger protein 24 | ZNF24 | 42.1 | 26 | 3 | 1 | 1 | 1 | 2.73e+06 |
| Q9BX97 | Plasmalemma vesicle-associated protein | PLVAP | 50.6 | 26 | 1 | 1 | 1 | 1 | 4.36e+07 |
| Q9NPQ8 | Synembryn-A | RIC8A | 59.7 | 25 | 1 | 1 | 1 | 1 | 3.04e+07 |
| Q9Y265 | RuvB-like 1 | RUVBL1 | 50.2 | 25 | 3 | 1 | 1 | 1 | 5.01e+06 |
| Q9NR12 | PDZ and LIM domain protein 7 | PDLIM7 | 49.8 | 25 | 2 | 1 | 1 | 1 | 6.28e+06 |
| Q9NZZ3 | Charged multivesicular body protein 5 | CHMP5 | 24.6 | 25 | 3 | 1 | 1 | 1 | 1.36e+07 |
| P49643 | DNA primase large subunit | PRIM2 | 58.8 | 25 | 2 | 1 | 1 | 1 |  |
| Q15459 | Splicing factor 3A subunit 1 | SF3A1 | 88.8 | 25 | 1 | 1 | 1 | 1 | 4.36e+06 |
| Q15029 | 116 kDa U5 small nuclear ribonucleoprotein component | EFTUD2 | 109.4 | 25 | 1 | 1 | 1 | 1 | 2.06e+06 |
| Q8WZ75 | Roundabout homolog 4 | ROBO4 | 107.4 | 24 | 1 | 1 | 1 | 1 | 1.38e+07 |
| O75487 | Glypican-4 | GPC4 | 62.4 | 24 | 3 | 1 | 1 | 1 | 5.87e+06 |
| Q13242 | Serine/arginine-rich splicing factor 9 | SRSF9 | 25.5 | 24 | 5 | 1 | 1 | 1 | 7.32e+06 |
| Q15464 | SH2 domain-containing adapter protein B | SHB | 55 | 24 | 3 | 1 | 1 | 1 | 4.95e+06 |
| Q96KP1 | Exocyst complex component 2 | EXOC2 | 104 | 24 | 1 | 1 | 1 | 1 |  |
| Q12849 | G-rich sequence factor 1 | GRSF1 | 53.1 | 24 | 2 | 1 | 1 | 1 | 4.75e+06 |
| P49759 | Dual specificity protein kinase CLK1 | CLK1 | 57.3 | 24 | 1 | 1 | 1 | 1 | 6.86e+06 |
| Q8IVT5 | Kinase suppressor of Ras 1 | KSR1 | 102.1 | 24 | 1 | 1 | 1 | 1 | 1.75e+07 |
| Q92830 | Histone acetyltransferase KAT2A | KAT2A | 93.9 | 23 | 1 | 1 | 1 | 1 | 3.30e+07 |
| P50238 | Cysteine-rich protein 1 | CRIP1 | 8.5 | 23 | 9 | 1 | 1 | 1 | 2.40e+08 |
| P49321 | Nuclear autoantigenic sperm protein | NASP | 85.2 | 23 | 1 | 1 | 1 | 1 | 2.50e+06 |
| O43684 | Mitotic checkpoint protein BUB3 | BUB3 | 37.1 | 23 | 2 | 1 | 1 | 1 | 5.02e+06 |
| O95639 | Cleavage and polyadenylation specificity factor subunit 4 | CPSF4 | 30.2 | 23 | 4 | 1 | 1 | 1 | 2.17e+06 |
| O60942 | mRNA-capping enzyme | RNGTT | 68.5 | 23 | 2 | 1 | 1 | 1 | 1.91e+06 |
| O75400 | Pre-mRNA-processing factor 40 homolog A | PRPF40A | 108.7 | 23 | 1 | 1 | 1 | 1 | 3.92e+06 |
| P62273 | Small ribosomal subunit protein uS14 | RPS29 | 6.7 | 23 | 20 | 1 | 1 | 1 | 7.63e+06 |
| Q09666 | Neuroblast differentiation-associated protein AHNAK | AHNAK | 628.7 | 23 | 0 | 1 | 1 | 1 |  |
| Q14527 | Helicase-like transcription factor | HLTF | 113.9 | 23 | 1 | 1 | 1 | 1 | 3.10e+06 |
| Q9NR30 | Nucleolar RNA helicase 2 | DDX21 | 87.3 | 23 | 1 | 1 | 1 | 1 | 2.44e+06 |
| O43291 | Kunitz-type protease inhibitor 2 | SPINT2 | 28.2 | 23 | 3 | 1 | 1 | 1 | 4.55e+06 |
| Q96ET8 | Golgi apparatus membrane protein TVP23 homolog C | TVP23C | 31.1 | 23 | 3 | 1 | 1 | 1 | 4.16e+07 |
| Q05BV3 | Echinoderm microtubule-associated protein-like 5 | EML5 | 219.3 | 23 | 0 | 1 | 1 | 1 | 3.04e+06 |
| Q5VTL8 | Pre-mRNA-splicing factor 38B | PRPF38B | 64.4 | 23 | 2 | 1 | 1 | 1 | 3.87e+06 |
| Q08J23 | RNA cytosine C(5)-methyltransferase NSUN2 | NSUN2 | 86.4 | 22 | 1 | 1 | 1 | 1 |  |
| P69905 | Hemoglobin subunit alpha | HBA1 | 15.2 | 22 | 11 | 1 | 1 | 1 | 4.19e+06 |
| P49458 | Signal recognition particle 9 kDa protein | SRP9 | 10.1 | 22 | 9 | 1 | 1 | 1 | 5.54e+06 |
| Q9UHX1 | Poly(U)-binding-splicing factor PUF60 | PUF60 | 59.8 | 22 | 2 | 1 | 1 | 1 | 2.92e+06 |
| Q08945 | FACT complex subunit SSRP1 | SSRP1 | 81 | 22 | 1 | 1 | 1 | 1 | 3.52e+06 |
| Q8IVM0 | Coiled-coil domain-containing protein 50 | CCDC50 | 35.8 | 21 | 3 | 1 | 1 | 1 | 7.35e+06 |
| P61619 | Protein transport protein Sec61 subunit alpha isoform 1 | SEC61A1 | 52.2 | 21 | 2 | 1 | 1 | 1 | 5.61e+06 |
| Q8NBS9 | Thioredoxin domain-containing protein 5 | TXNDC5 | 47.6 | 21 | 2 | 1 | 1 | 1 | 2.91e+06 |
| P09234 | U1 small nuclear ribonucleoprotein C | SNRPC | 17.4 | 21 | 11 | 1 | 1 | 1 | 3.59e+06 |
| Q9Y586 | Protein mab-21-like 2 | MAB21L2 | 40.9 | 21 | 2 | 1 | 1 | 1 | 8.88e+06 |
| P43307 | Translocon-associated protein subunit alpha | SSR1 | 32.2 | 21 | 3 | 1 | 1 | 1 | 8.23e+06 |
| O95470 | Sphingosine-1-phosphate lyase 1 | SGPL1 | 63.5 | 20 | 2 | 1 | 1 | 1 | 1.53e+07 |
| Q14739 | Delta(14)-sterol reductase LBR | LBR | 70.7 | 20 | 1 | 1 | 1 | 1 | 4.40e+06 |
| P55265 | Double-stranded RNA-specific adenosine deaminase | ADAR | 136 | 20 | 1 | 1 | 1 | 1 | 3.07e+06 |
| Q96RT1 | Erbin | ERBIN | 158.2 | 20 | 0 | 1 | 1 | 1 | 1.11e+07 |
| Q9UKP5 | A disintegrin and metalloproteinase with thrombospondin motifs 6 | ADAMTS6 | 125.2 | 20 | 1 | 1 | 1 | 1 | 4.94e+07 |
| Q6NXG1 | Epithelial splicing regulatory protein 1 | ESRP1 | 75.5 | 20 | 1 | 1 | 1 | 1 | 1.32e+06 |

Supplementary Table 5. Primer Sequences for qPCR.

| Gene Name | Primer Sequence |
| --- | --- |
| human INHBA | Forward primer: 5’- TGCTCCCTCTGGCTATCATG-3’ |
|  | Reverse primer: 5’-TGCGGTAGTGGTTGATGACT-3’ |
| human SLC25A10 | Forward primer: 5’-ctcgtgaagagggtctcagg-3’ |
|  | Reverse primer: 5’-tggcgacaaagtgagtgaag-3’ |
| human CD163 | Forward primer: 5’-GGCTTGCAGTTTCCTCAAGA-3’ |
|  | Reverse primer: 5’-GACACAGAAATTAGTTCAGCAGCA-3’ |
| human VEGF | Forward primer: 5’-CGGTATAAGTCCTGGAGCGT-3’ |
|  | Reverse primer: 5’-TTTAACTCAAGCTGCCTCGC-3’ |
| human IL-1α | Forward primer: 5’-TGCTACTTTATGGGCAGCAG-3’ |
|  | Reverse primer: 5’-GGTCGGCAGATCGTCTCTAAA-3’ |
| human TGF-β1 | Forward primer: 5’-CTTTCCTGCTTCTCATGGCC-3’ |
|  | Reverse primer: 5’-TCCAGGCTCCAAATGTAGGG-3’ |
| human Arg-1 | Forward primer: 5’-CCCTTTGCTGACATCCCTAA-3’ |
|  | Reverse primer: 5’-ATTGCCAAACTGTGGTCTCC-3’ |
| human CD206 | Forward primer: 5’-GGCCAAGCTTCTCTGGAATG-3’ |
|  | Reverse primer: 5’-ACAAAGGAGACCGGACTGTT-3’ |
| human IL-10 | Forward primer: 5’-GGCACCCAGTCTGAGAACAG-3’ |
|  | Reverse primer: 5’-TGGCAACCCAGGTAACCCTTA-3’ |
| human GAPDH | Forward primer: 5’-CGACCACTTTGTCAAGCTCA-3’ |
|  | Reverse primer: 5’-ACTGAGTGTGGCAGGGACTC-3’ |
| mouse INHBA | Forward primer: 5’-TTGCTCCCTCTGGCTATCAC-3’ |
|  | Reverse primer: 5’-CCCTCATGCGGTAGTGGTTA-3’ |
| mouse SLC25A10 | Forward primer: 5’-AGCAACTGGTCCTCAGCACT-3’ |
|  | Reverse primer: 5’-GTCCAAGCTTTGCTGTCTCC-3’ |
| mouse GAPDH | Forward primer: 5’-ATGGTGAAGGTCGGTGTGAA-3’ |
|  | Reverse primer: 5’-CATTCTCGGCCTTGACTGTG-3’ |

Supplementary Table 6. Antibodies were used in this study for Western Blot analysis.

| Antibodies | Source | Catalog |
| --- | --- | --- |
| Anti-INHBA | HUABIO | HA500271 |
| Anti-DYKDDDDK (Flag) | HUABIO | HA601185 |
| Anti-SLC25A10 | Proteintech | 12086-1-AP |
| Anti-GPX4 | HUABIO | ET1706-45 |
| Anti-MYC | Proteintech | 60003-2-Ig |
| Anti-HA | Proteintech | 66006-2-Ig |
| Anti-ubiquitin | Proteintech | 10201-2-AP |
| Anti-TRIM21 | Beyotime | AG3311 |
| Anti-His | OriGene | TA150088 |
| Anti-SLC25A10 | Solarbio | K108798P |
| Anti-GAPDH | HUABIO | ET1702-66 |
| Anti-COX IV | Proteintech | 11242-1-AP |
| Anti-ZO1 | Abiowell | AWA11245 |
| Anti-E-Cadherin | Abiowell | AWA10139 |
| Anti-N-Cadherin | HUABIO | ET1607-37 |
| Anti-Vimentin | ABclonal | A19607 |
| Anti-β-Tubulin | ImmunoWay | YM3030 |

Supplementary Table 7. Antibodies were used in this study for immunofluorescence analysis.

| Antibodies | Source | Catalog |
| --- | --- | --- |
| Anti-CD163 (cell) | AiFang biological | AF20010 |
| Anti-CD206 (cell) | CST | #24595 |
| Anti-Inhibin beta A (cell) | Abcam | ab97705 |
| Anti-SLC25A10 (cell) | Proteintech | 12086-1-AP |
| Anti-γ-H2AX (cell) | HUABIO | ET1602–2 |
| Anti-53BP1 (cell) | Abiowell | AWA11931 |
| Anti-Inhibin beta A (tissue) | Proteintech | 60352-1-Ig |
| Anti-CD68 (tissue) | GeneTex | GM087629 |

Supplementary Table 8. Antibodies were used in this study for immunohistochemical analysis.

| Antibodies | Source | Catalog |
| --- | --- | --- |
| Anti-F4/80 | Servicebio | GB11027 |
| Anti-CD206 | CST | #24595 |
| Anti-Ki-67 | AiFang biological | SAF008 |
| Anti-CD8 | AiFang biological | AFRM0004 |
| Anti-GZMB | AiFang biological | AFRM0352 |
| Anti-CD11c | AiFang biological | AFRM0294 |
| Anti-Inhibin beta A | Proteintech | 10651-1-AP |
| Anti-SLC25A10 | Proteintech | 12086-1-AP |

Supplementary Table 9. Antibodies were used in this study for flow cytometry analysis.

| Antibodies | Source | Catalog |
| --- | --- | --- |
| PE anti-human CD68 | BioLegend | 333808 |
| PE/Cyanine7 anti-human CD206 | BioLegend | 321124 |
| APC anti-human CD163 | BioLegend | 333610 |
| Alexa Fluor^®^ 488 Rat Anti-CD11b | BD | 557672 |
| PE Rat Anti-Mouse F4/80 | BD | 565410 |
| APC-Cy™7 Rat Anti-Mouse CD45 | BD | 557659 |
| PE/Cyanine7 anti-mouse CD163 | BioLegend | 155320 |
| Alexa Fluor™ 647 Rat Anti-Mouse CD206 | BD | 568808 |
