# Supplementary material for: Inhibin beta A drives colorectal cancer progression through macrophage M2 polarization and mitochondria-dependent ferroptosis suppression
Source: Signal Transduct Target Ther. 2025 Dec 26;10:420. doi: 10.1038/s41392-025-02518-y (PMC12741050; doi:10.1038/s41392-025-02518-y)
Supplement: Supplementary file 2 — the full uncropped Gels and Blots images [file 41392_2025_2518_MOESM2_ESM.pdf]

**Fig. 5c**

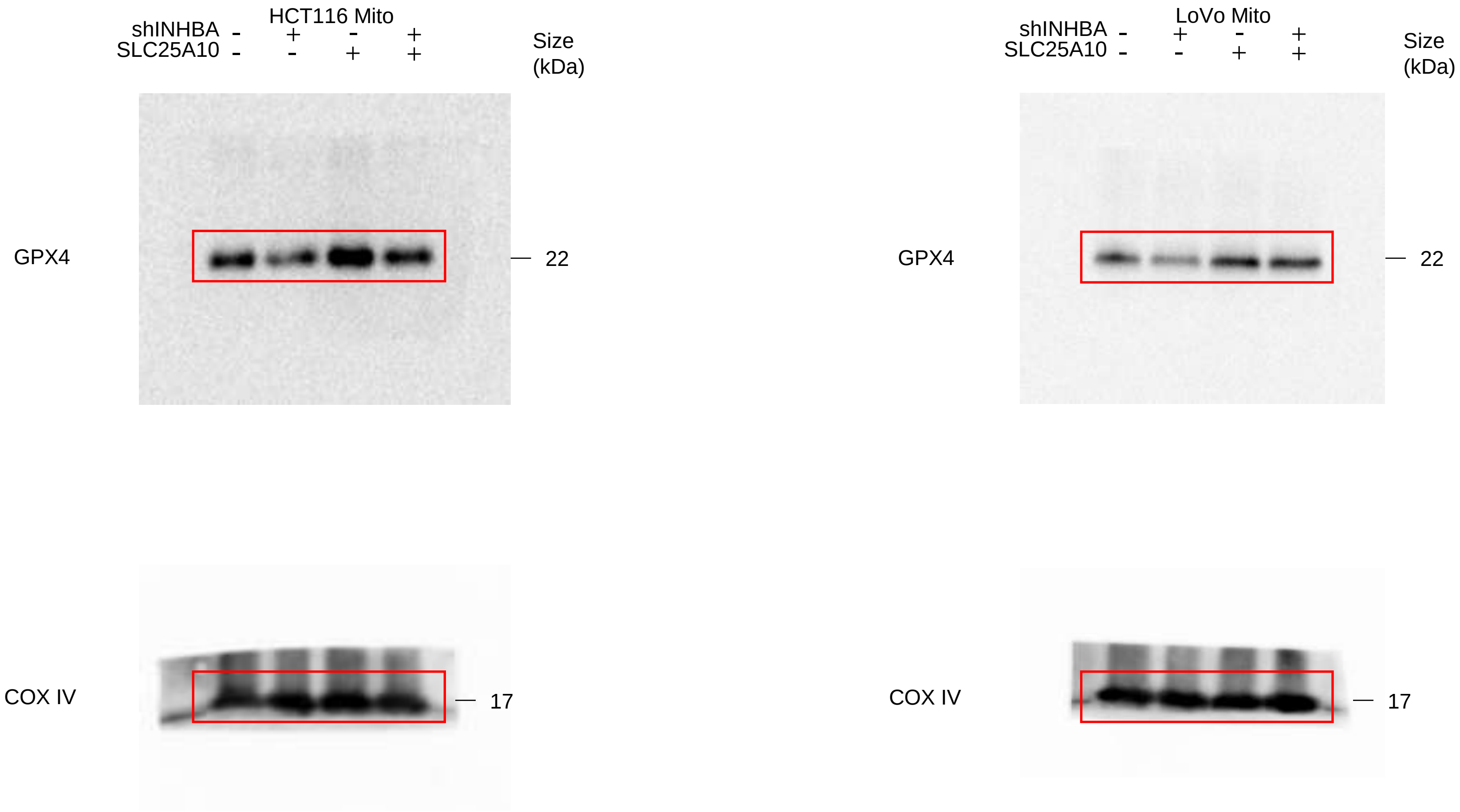
HCT116 Mito
shINHBA - + - +
SLC25A10 - - + +
Size (kDa)
GPX4
22
COX IV
17
LoVo Mito
shINHBA - + - +
SLC25A10 - - + +
Size (kDa)
GPX4
22
COX IV
17

**Fig. 5e**

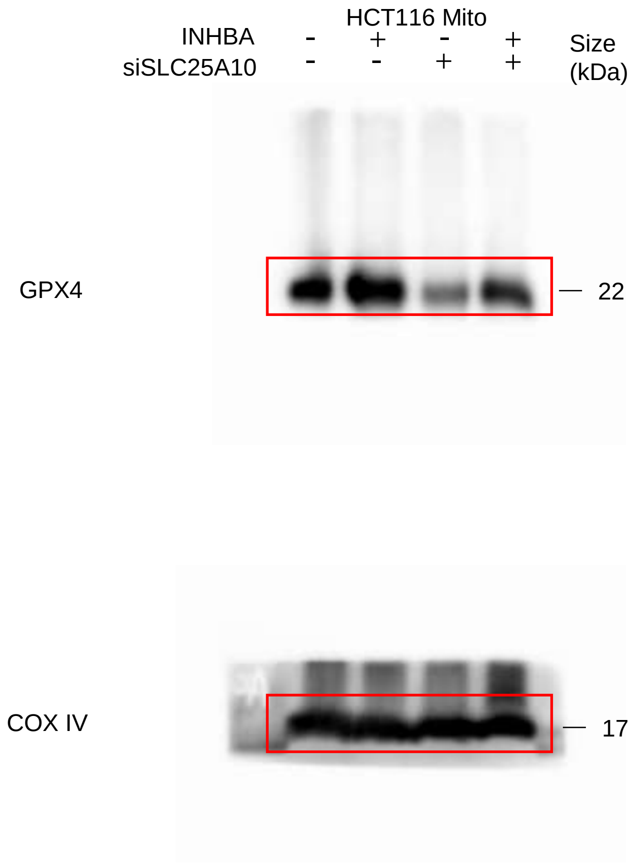

HCT116 Mito
INHBA - + - +
siSLC25A10 - - + +
Size (kDa)
GPX4
22
COX IV
17


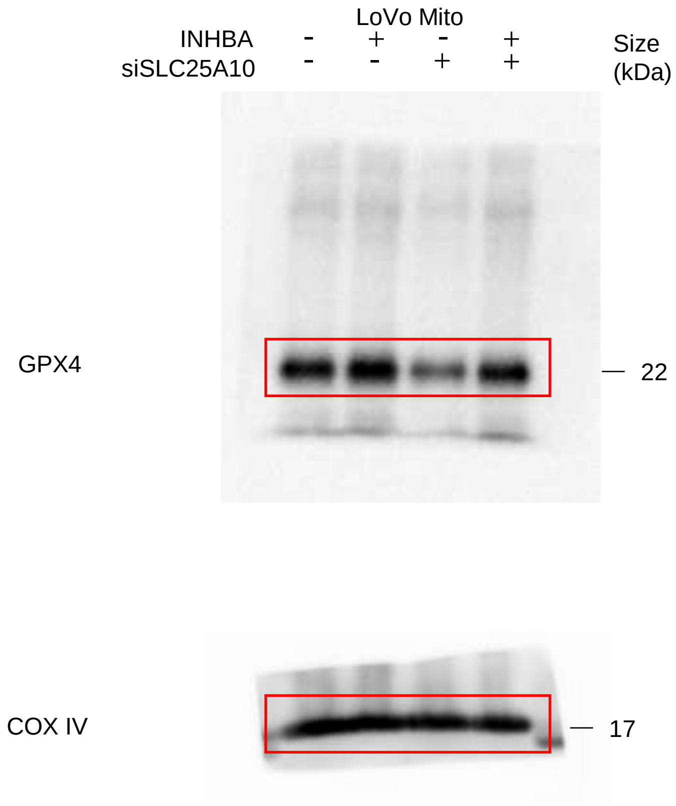

LoVo Mito
INHBA - + - +
siSLC25A10 - - + +
Size (kDa)
GPX4
22
COX IV
17

Fig. 6a

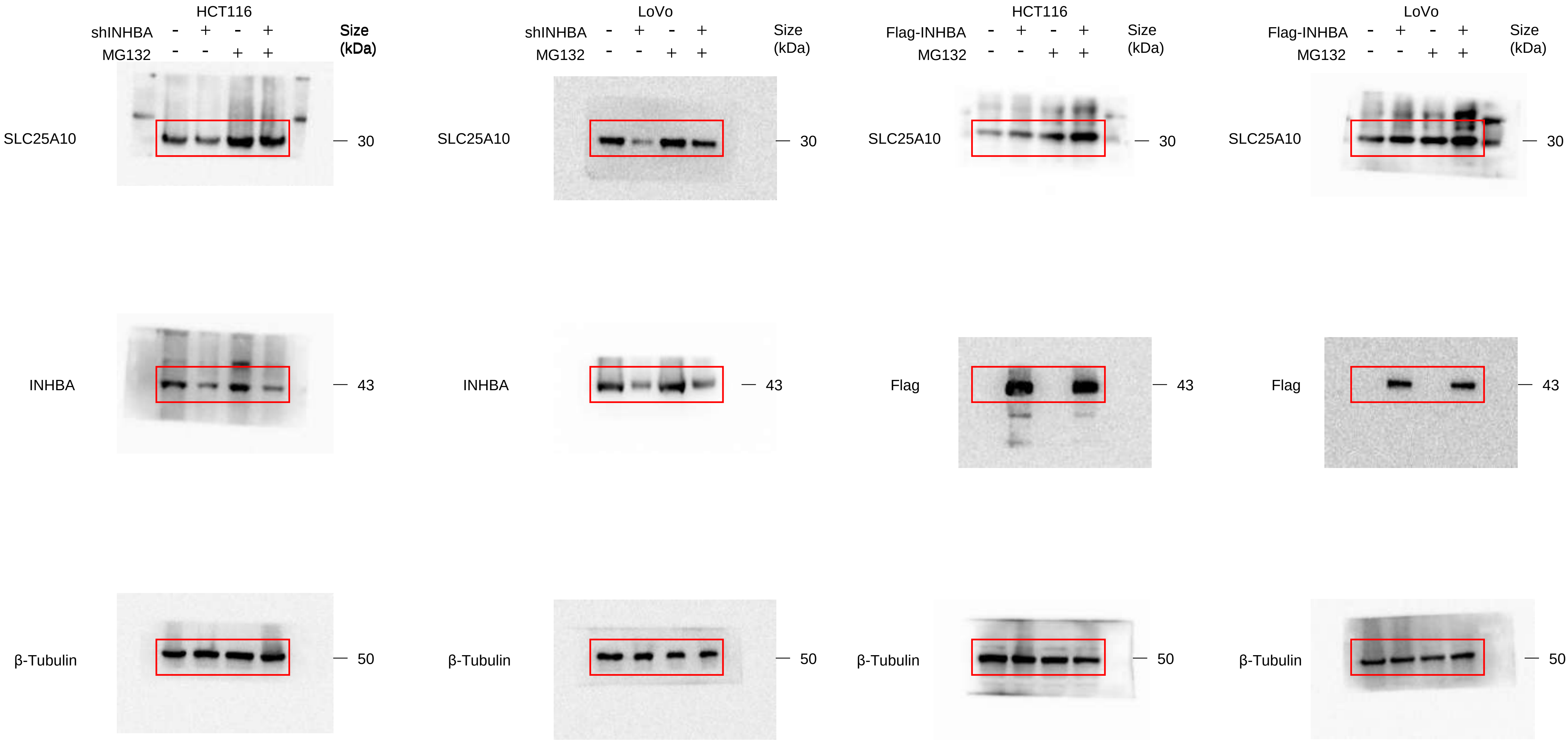
HCT116
shINHBA - + - +
MG132 - - + +
Size (kDa)
SLC25A10
30
INHBA
43
β-Tubulin
50
LoVo
shINHBA - + - +
MG132 - - + +
Size (kDa)
SLC25A10
30
INHBA
43
β-Tubulin
50
HCT116
Flag-INHBA - + - +
MG132 - - + +
Size (kDa)
SLC25A10
30
Flag
43
β-Tubulin
50
LoVo
Flag-INHBA - + - +
MG132 - - + +
Size (kDa)
SLC25A10
30
Flag
43
β-Tubulin
50

Fig. 6b

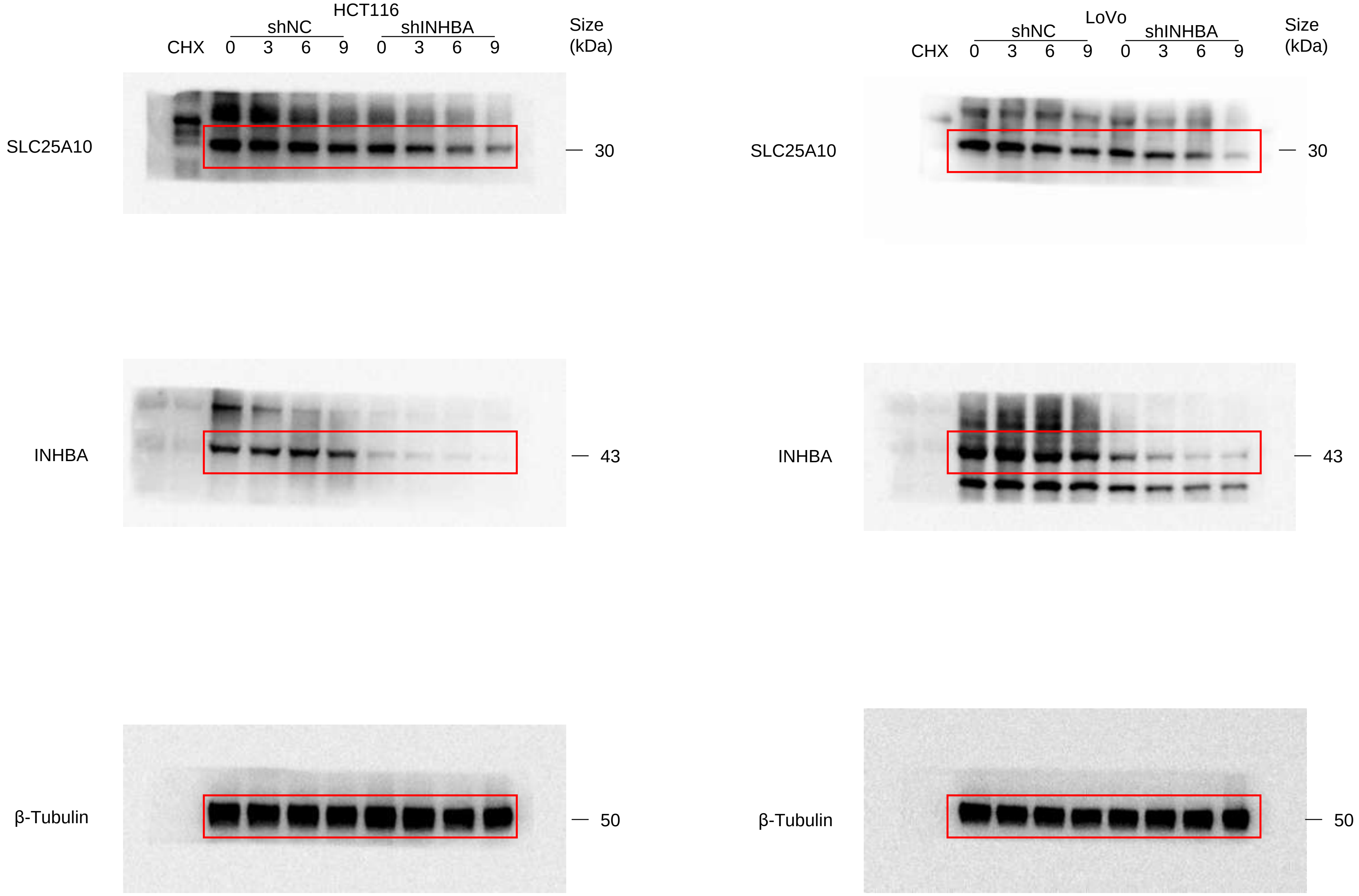
HCT116
shNC
shINHBA
CHX 0 3 6 9 0 3 6 9
Size (kDa)
SLC25A10
30
INHBA
43
β-Tubulin
50
LoVo
shNC
shINHBA
CHX 0 3 6 9 0 3 6 9
Size (kDa)
SLC25A10
30
INHBA
43
β-Tubulin
50

**Fig. 6c**

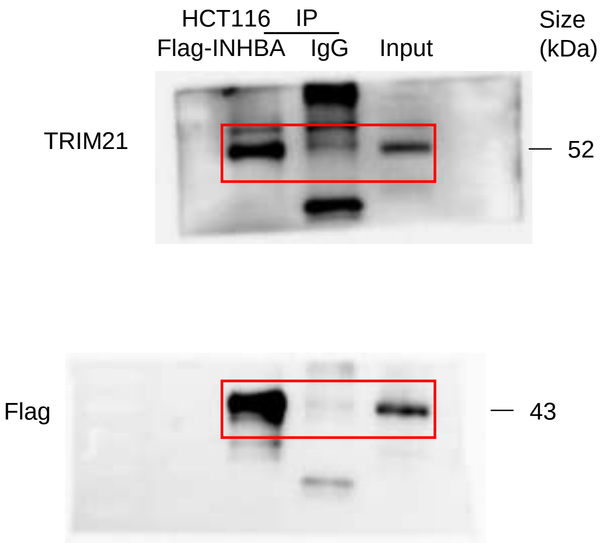
HCT116
IP
Flag-INHBA
IgG
Input
Size
(kDa)
TRIM21
52
Flag
43

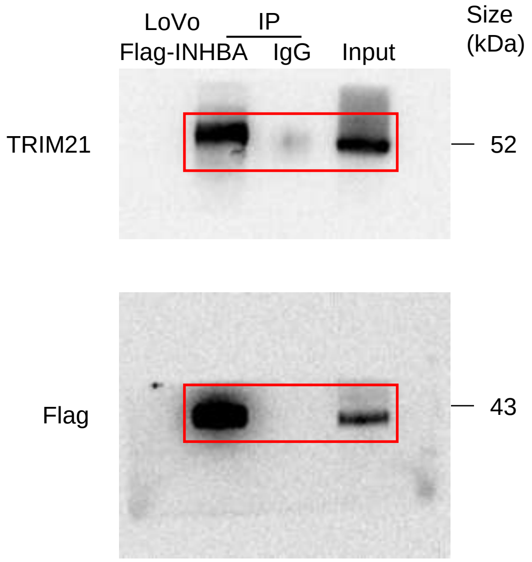
LoVo
IP
Flag-INHBA
IgG
Input
Size
(kDa)
TRIM21
52
Flag
43

Fig. 6d

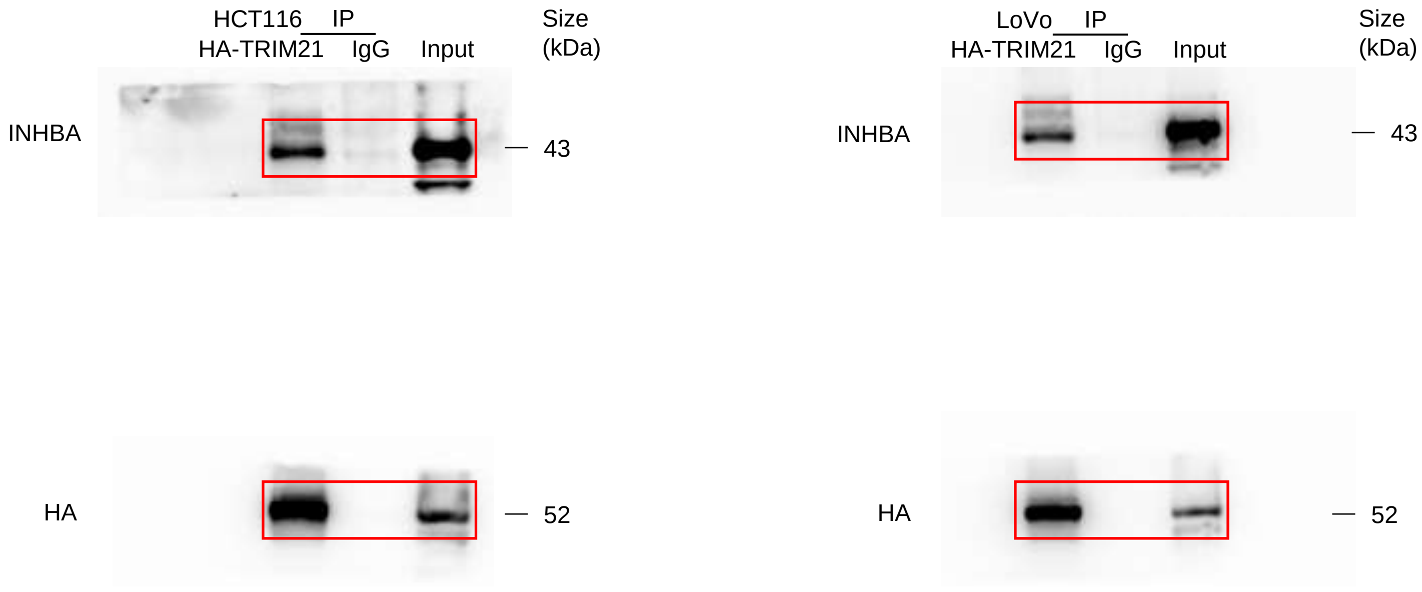
HCT116
IP
HA-TRIM21
IgG
Input
Size (kDa)
INHBA
43
HA
52
LoVo
IP
HA-TRIM21
IgG
Input
Size (kDa)
INHBA
43
HA
52

**Fig. 6e**

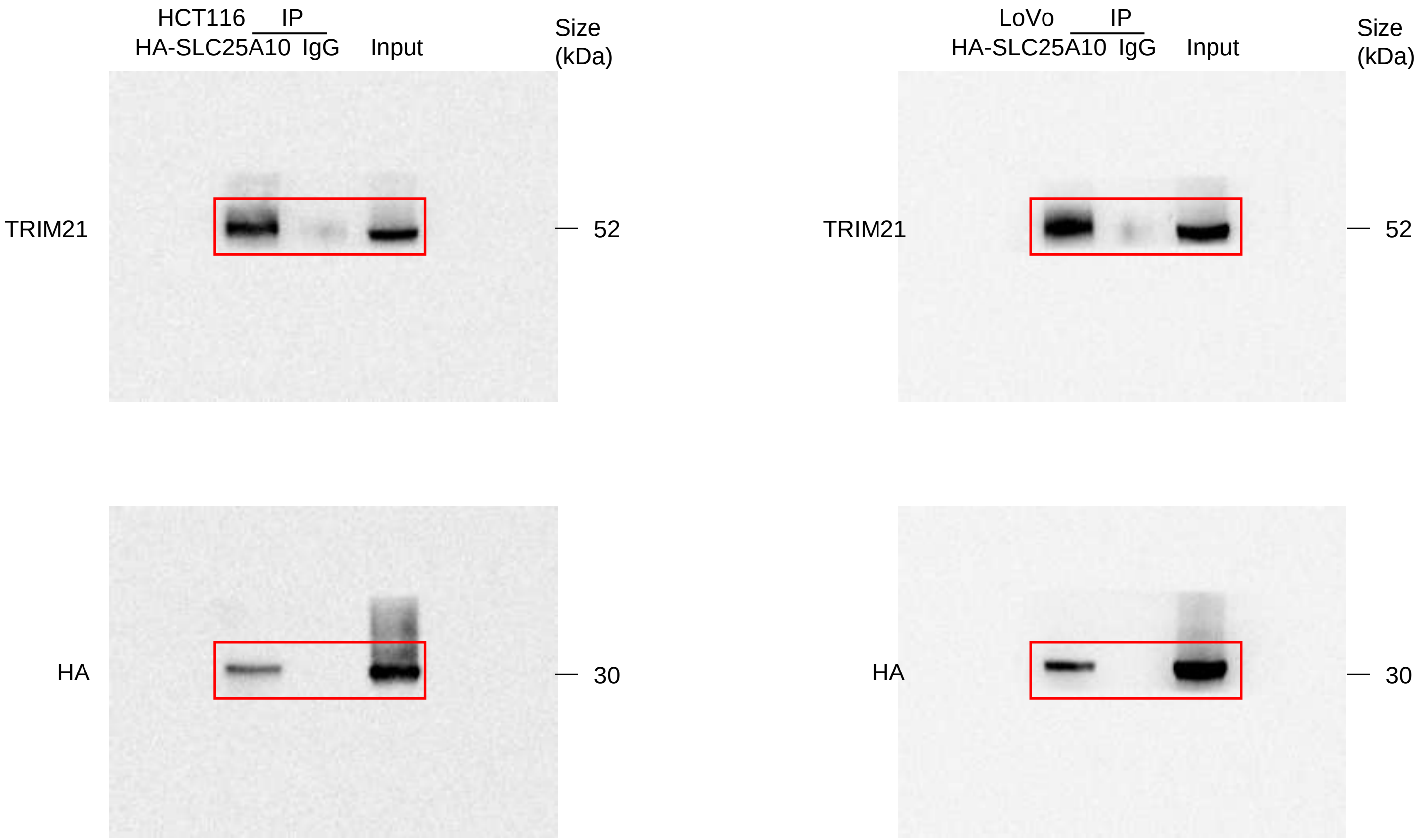
HCT116
IP
HA-SLC25A10
IgG
Input
Size (kDa)
TRIM21
52
HA
30
LoVo
IP
HA-SLC25A10
IgG
Input
Size (kDa)
TRIM21
52
HA
30

**Fig. 6f**

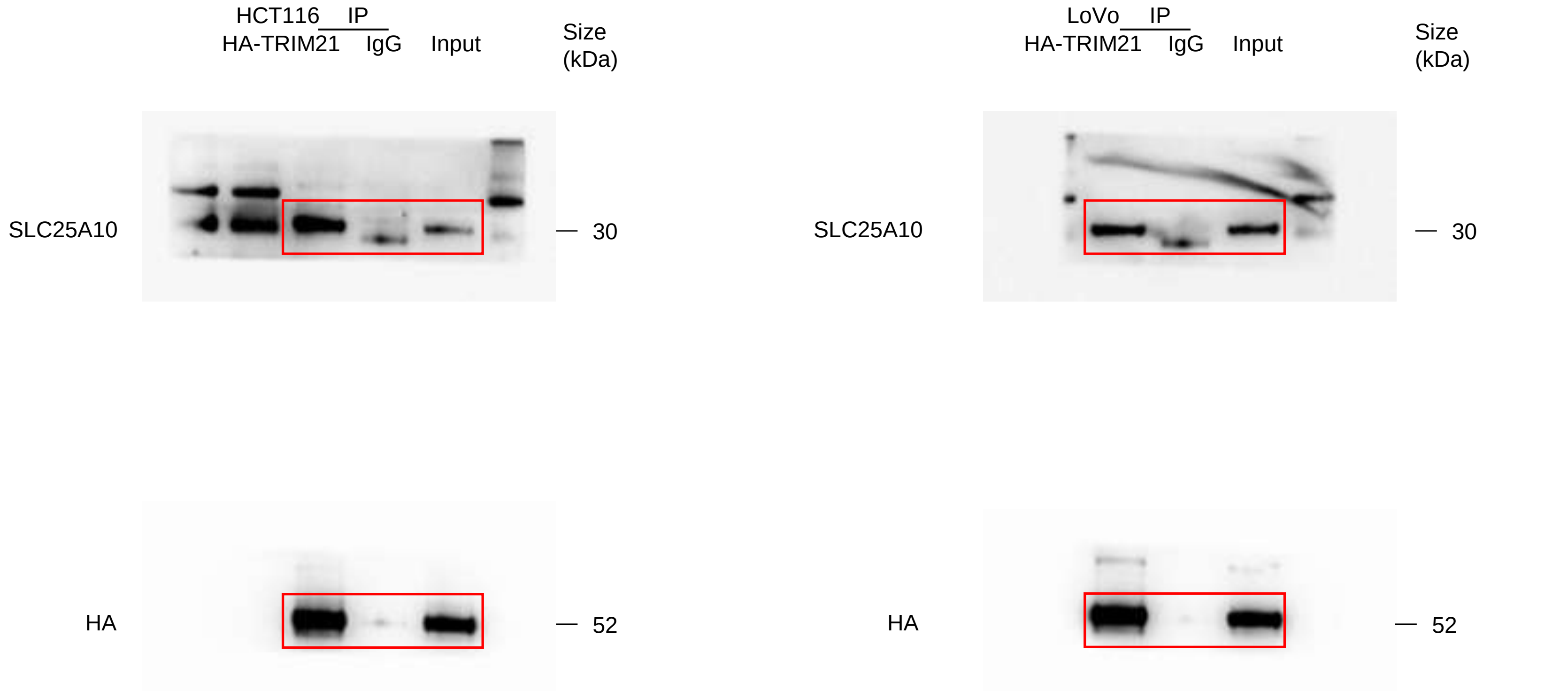
HCT116
IP
HA-TRIM21
IgG
Input
Size (kDa)
SLC25A10
30
HA
52
LoVo
IP
HA-TRIM21
IgG
Input
Size (kDa)
SLC25A10
30
HA
52

**Fig. 6g**

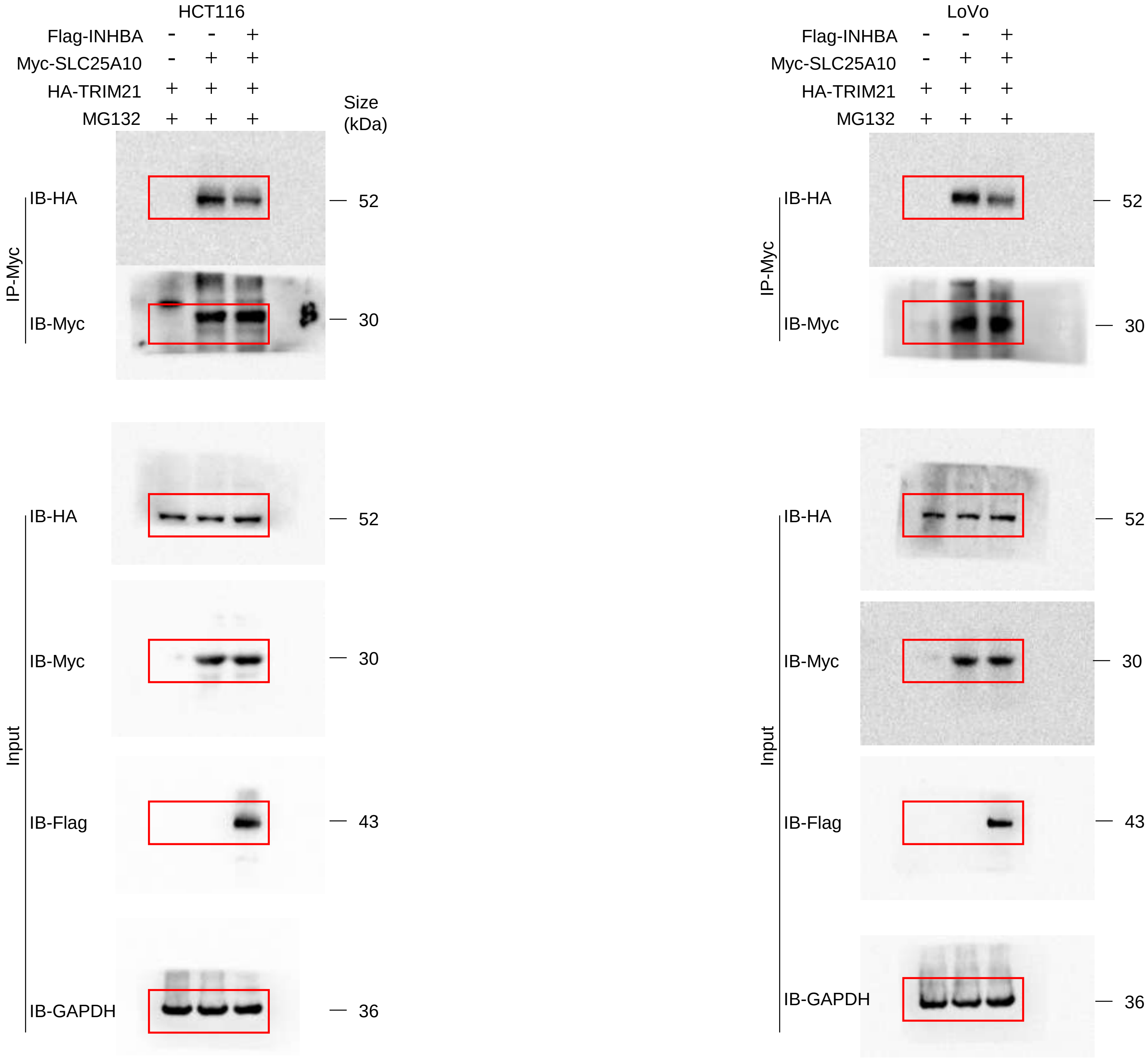
HCT116
Flag-INHBA - - +
Myc-SLC25A10 - + +
HA-TRIM21 + + +
MG132 + + +
Size (kDa)
IP-Myc
IB-HA
52
IB-Myc
30
Input
IB-HA
52
IB-Myc
30
IB-Flag
43
IB-GAPDH
36
LoVo
Flag-INHBA - - +
Myc-SLC25A10 - + +
HA-TRIM21 + + +
MG132 + + +
IP-Myc
IB-HA
52
IB-Myc
30
Input
IB-HA
52
IB-Myc
30
IB-Flag
43
IB-GAPDH
36

**Fig. 6h**

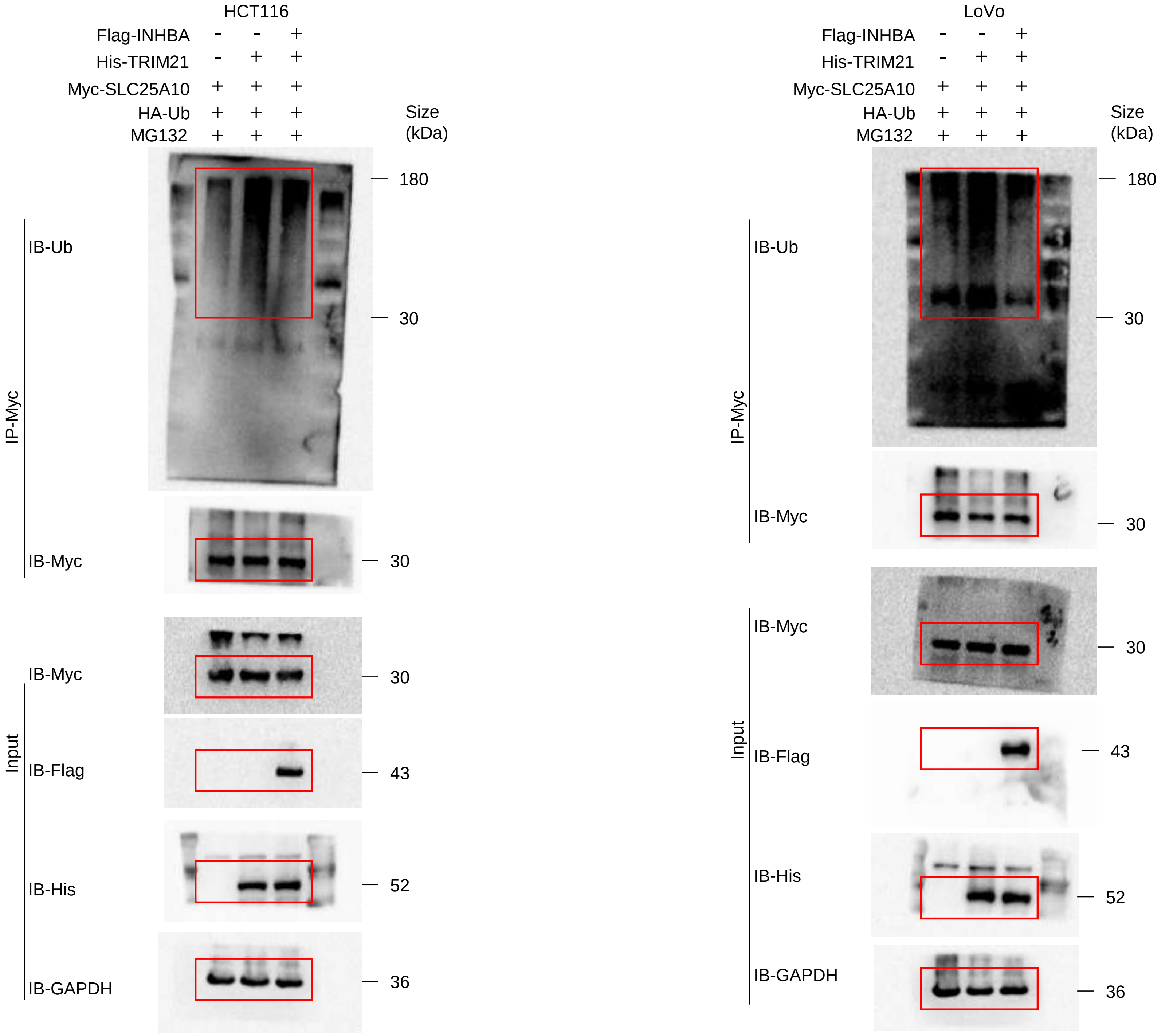

HCT116
Flag-INHBA - - +
His-TRIM21 - + +
Myc-SLC25A10 + + +
HA-Ub + + +
MG132 + + +
Size (kDa)
180
30
IB-Ub
IB-Myc
IP-Myc
IB-Myc
IB-Flag
IB-His
IB-GAPDH
Input
30
30
43
52
36
LoVo
Flag-INHBA - - +
His-TRIM21 - + +
Myc-SLC25A10 + + +
HA-Ub + + +
MG132 + + +
Size (kDa)
180
30
IB-Ub
IB-Myc
IP-Myc
IB-Myc
IB-Flag
IB-His
IB-GAPDH
Input
30
30
43
52
36

Fig. 6i

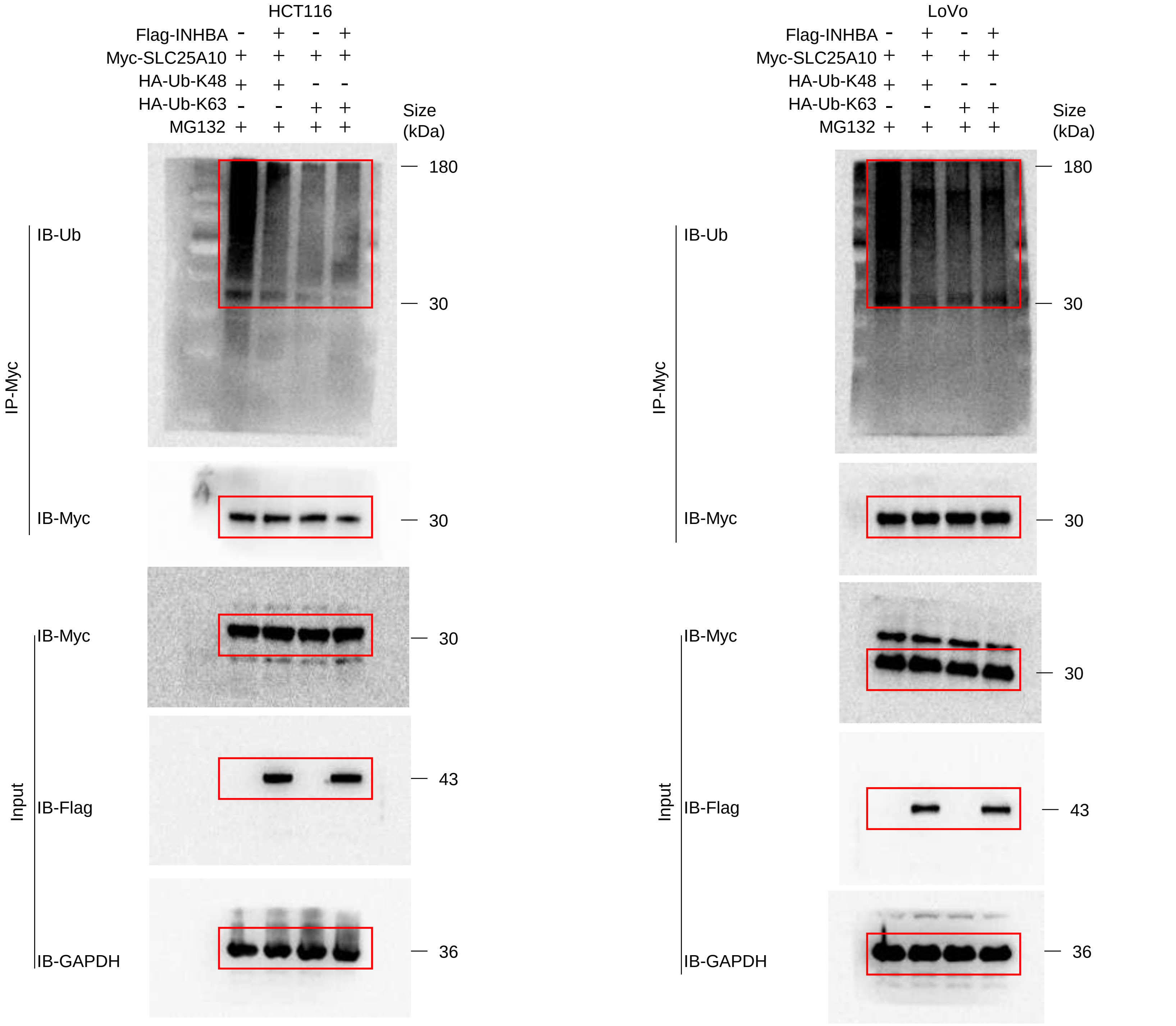
HCT116
LoVo
Flag-INHBA - + - +
Myc-SLC25A10 + + + +
HA-Ub-K48 + + - -
HA-Ub-K63 - - + +
MG132 + + + +
Size (kDa)
180
30
43
36
IP-Myc
Input
IB-Ub
IB-Myc
IB-Flag
IB-GAPDH

**Fig. 6j**

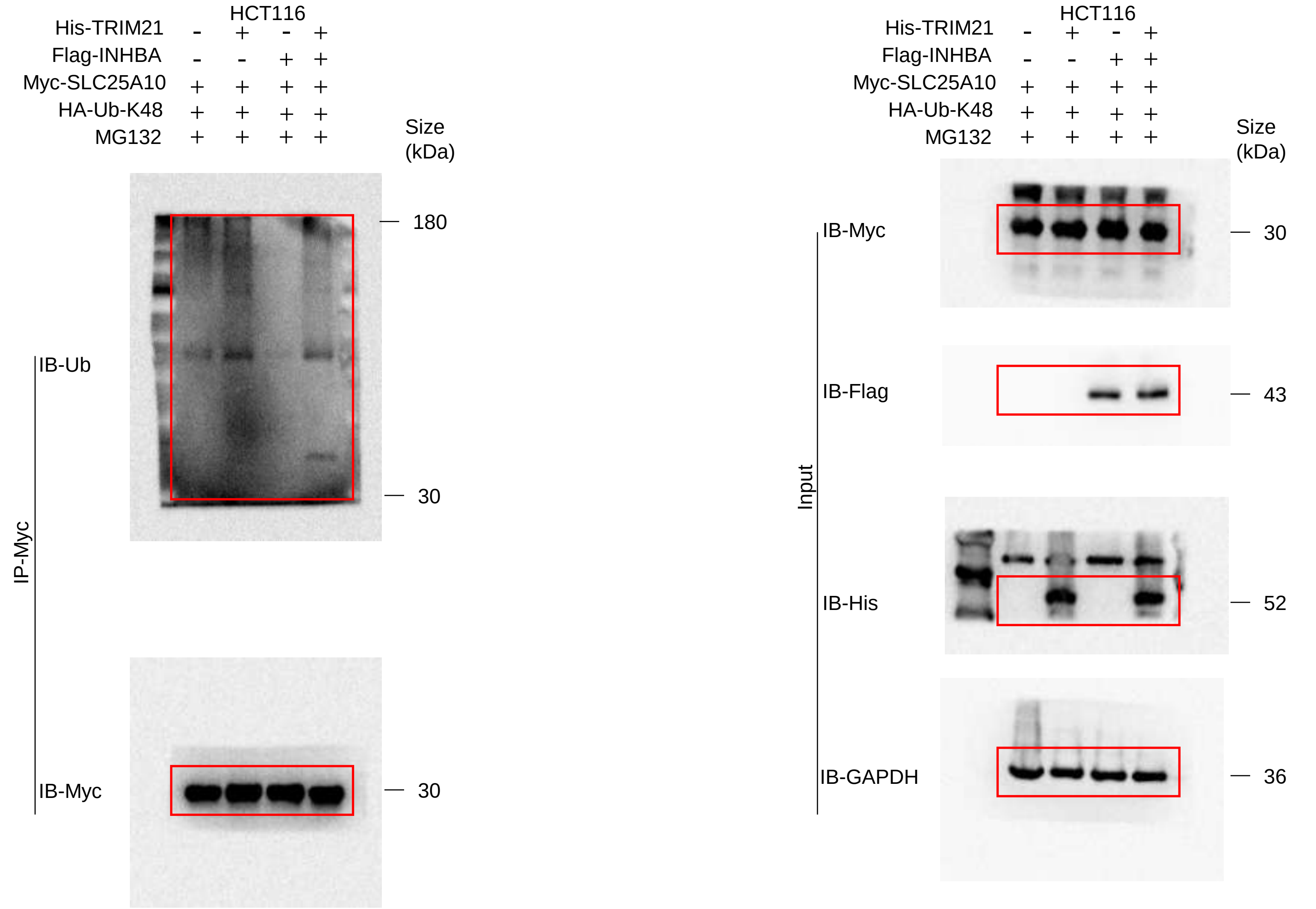
HCT116
His-TRIM21 - + - +
Flag-INHBA - - + +
Myc-SLC25A10 + + + +
HA-Ub-K48 + + + +
MG132 + + + +
Size (kDa)
IB-Ub
180
30
IP-Myc
IB-Myc
30
HCT116
His-TRIM21 - + - +
Flag-INHBA - - + +
Myc-SLC25A10 + + + +
HA-Ub-K48 + + + +
MG132 + + + +
Size (kDa)
IB-Myc
30
IB-Flag
43
Input
IB-His
52
IB-GAPDH
36

Fig. 6j

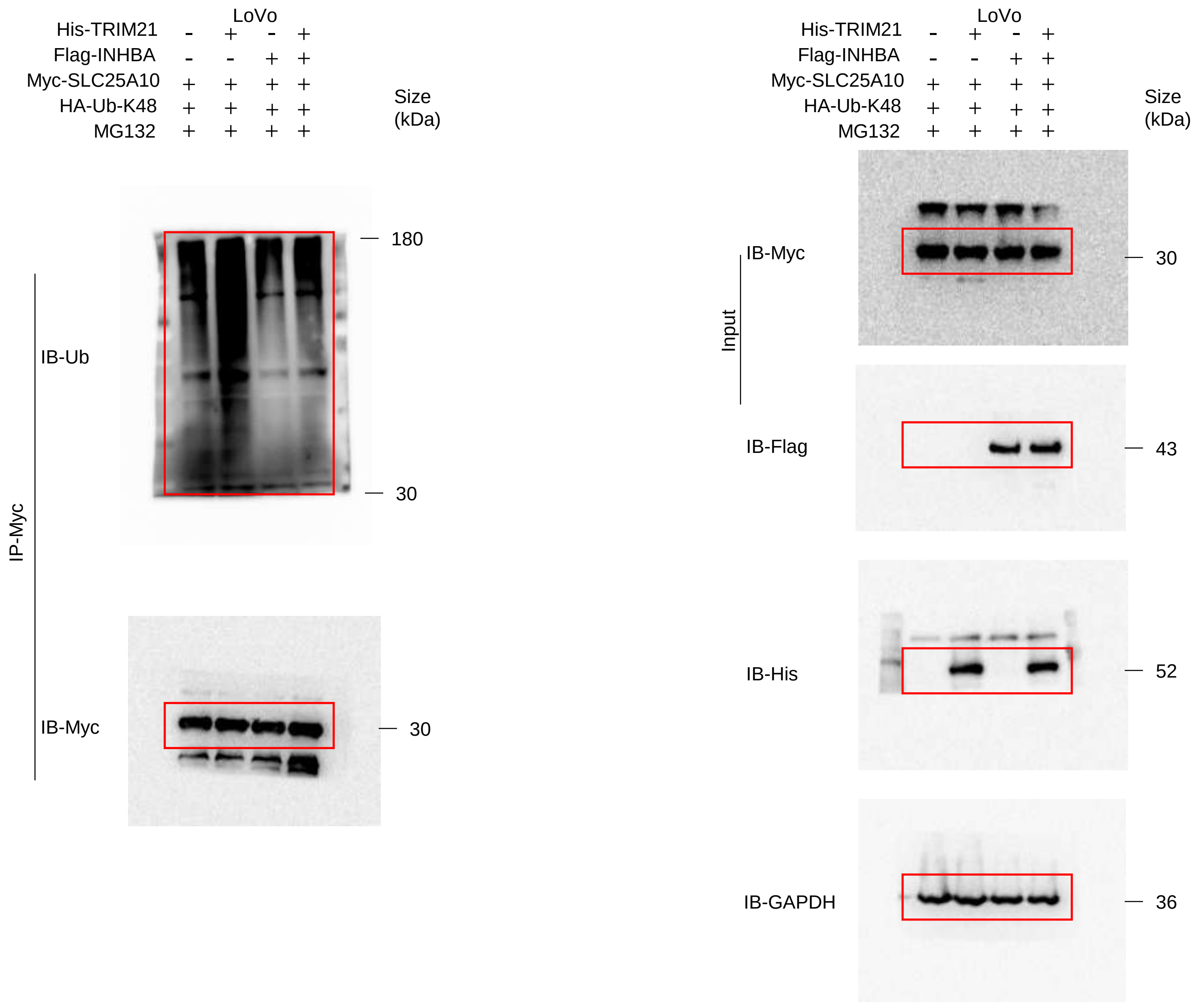

LoVo
His-TRIM21 - + - +
Flag-INHBA - - + +
Myc-SLC25A10 + + + +
HA-Ub-K48 + + + +
MG132 + + + +
Size (kDa)
IP-Myc
IB-Ub
180
30
IB-Myc
30
LoVo
His-TRIM21 - + - +
Flag-INHBA - - + +
Myc-SLC25A10 + + + +
HA-Ub-K48 + + + +
MG132 + + + +
Size (kDa)
Input
IB-Myc
30
IB-Flag
43
IB-His
52
IB-GAPDH
36

**Fig. S3b**

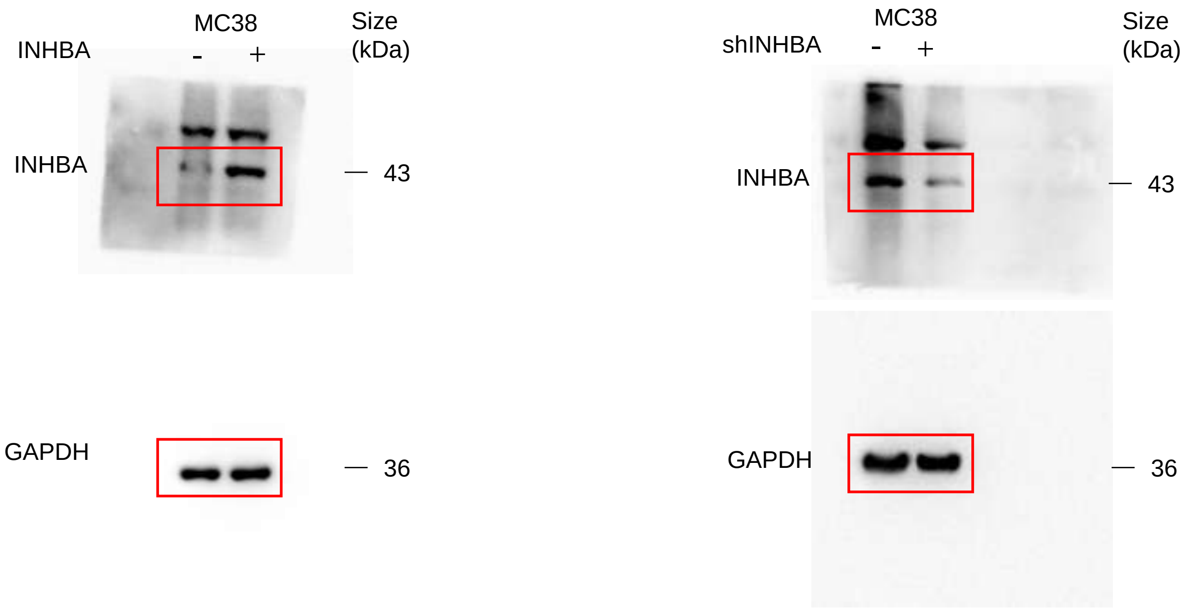
MC38
INHBA
-
+
Size
(kDa)
INHBA
43
GAPDH
36
MC38
shINHBA
-
+
Size
(kDa)
INHBA
43
GAPDH
36

**Fig. S5**

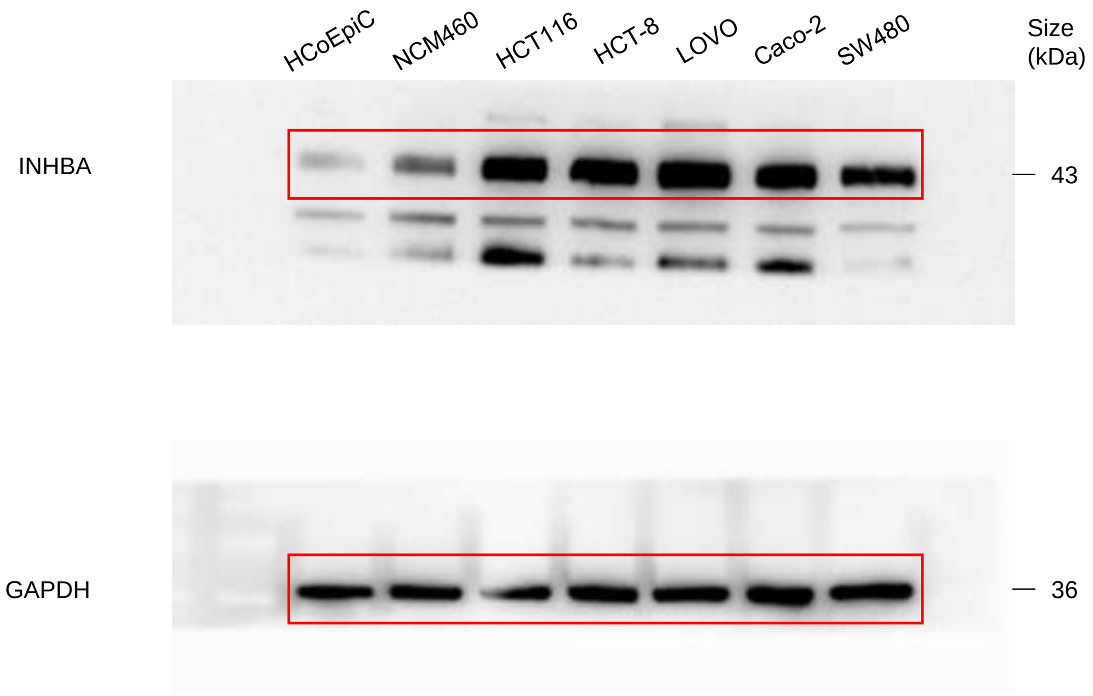
HCoEpiC
NCM460
HCT116
HCT-8
LOVO
Caco-2
SW480
Size
(kDa)
INHBA
43
GAPDH
36

**Fig. S6b**

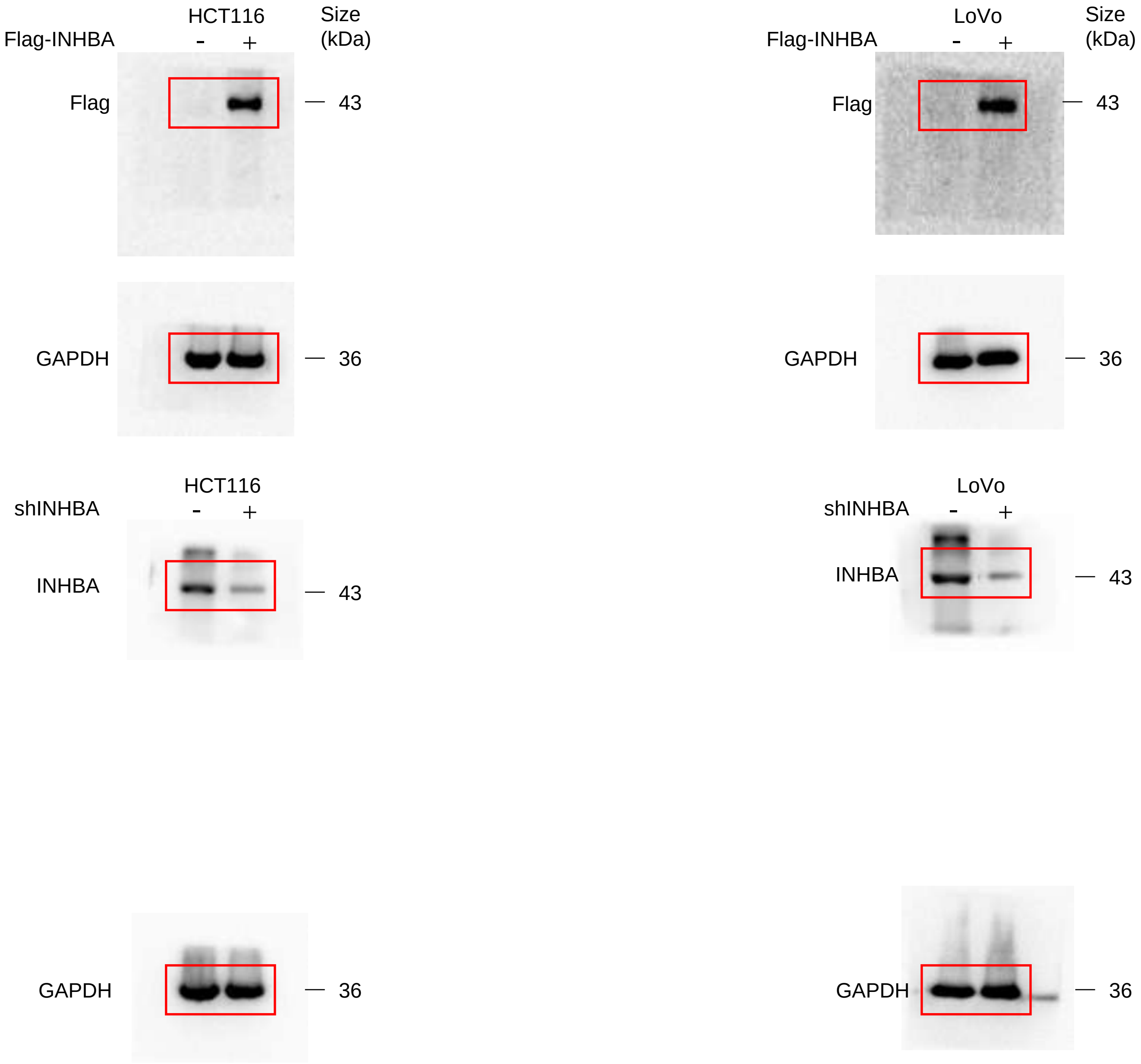
HCT116
Flag-INHBA
- +
Size
(kDa)
Flag
43
GAPDH
36
LoVo
Flag-INHBA
- +
Size
(kDa)
Flag
43
GAPDH
36
HCT116
shINHBA
- +
INHBA
43
LoVo
shINHBA
- +
INHBA
43
GAPDH
36
GAPDH
36

**Fig. S7a**

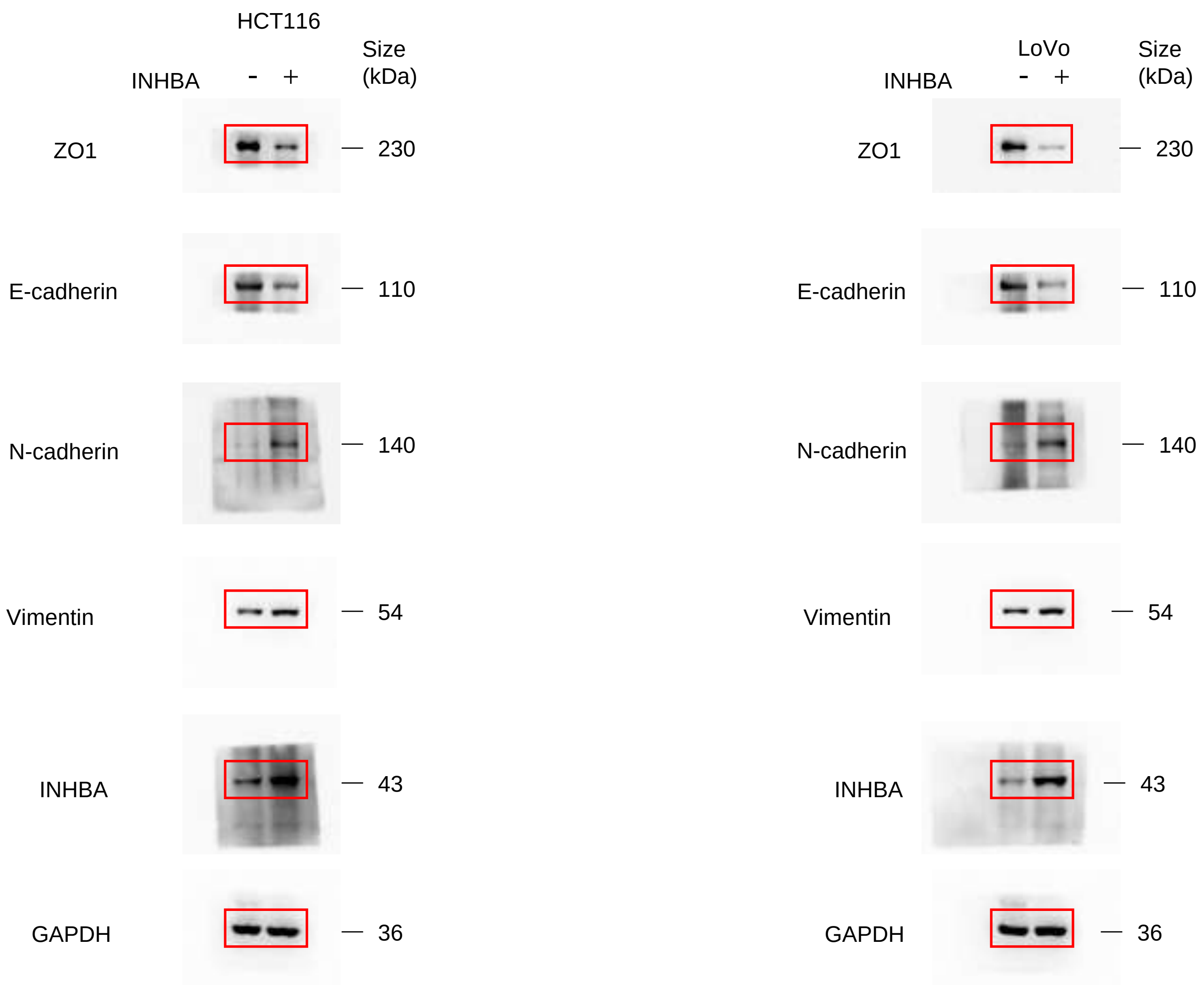

HCT116
INHBA - +
Size (kDa)
ZO1
230
E-cadherin
110
N-cadherin
140
Vimentin
54
INHBA
43
GAPDH
36
LoVo
INHBA - +
Size (kDa)
ZO1
230
E-cadherin
110
N-cadherin
140
Vimentin
54
INHBA
43
GAPDH
36

**Fig. S7b**

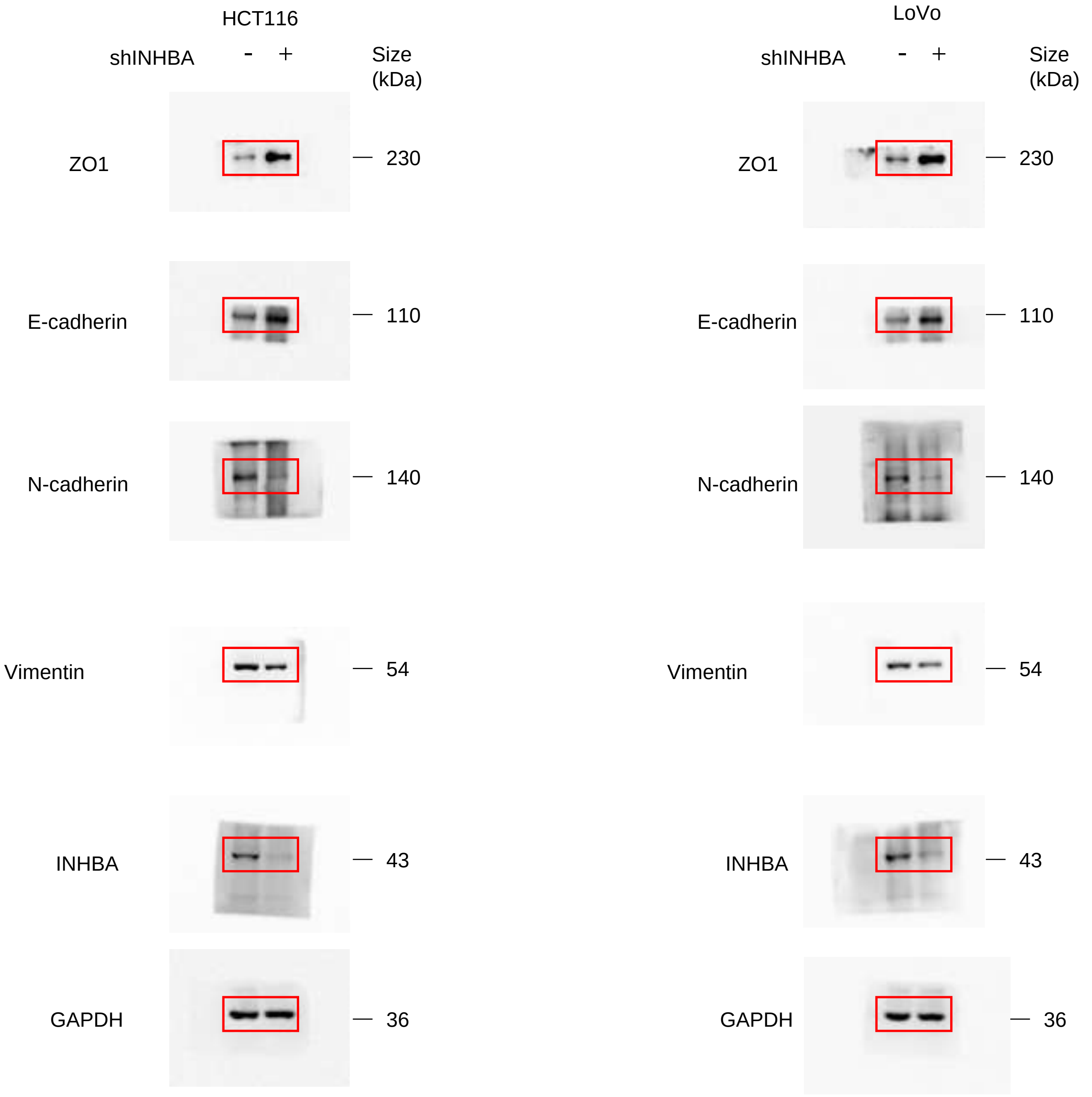
HCT116
shINHBA
-
+
Size
(kDa)
ZO1
230
E-cadherin
110
N-cadherin
140
Vimentin
54
INHBA
43
GAPDH
36
LoVo
shINHBA
-
+
Size
(kDa)
ZO1
230
E-cadherin
110
N-cadherin
140
Vimentin
54
INHBA
43
GAPDH
36

**Fig. S20**

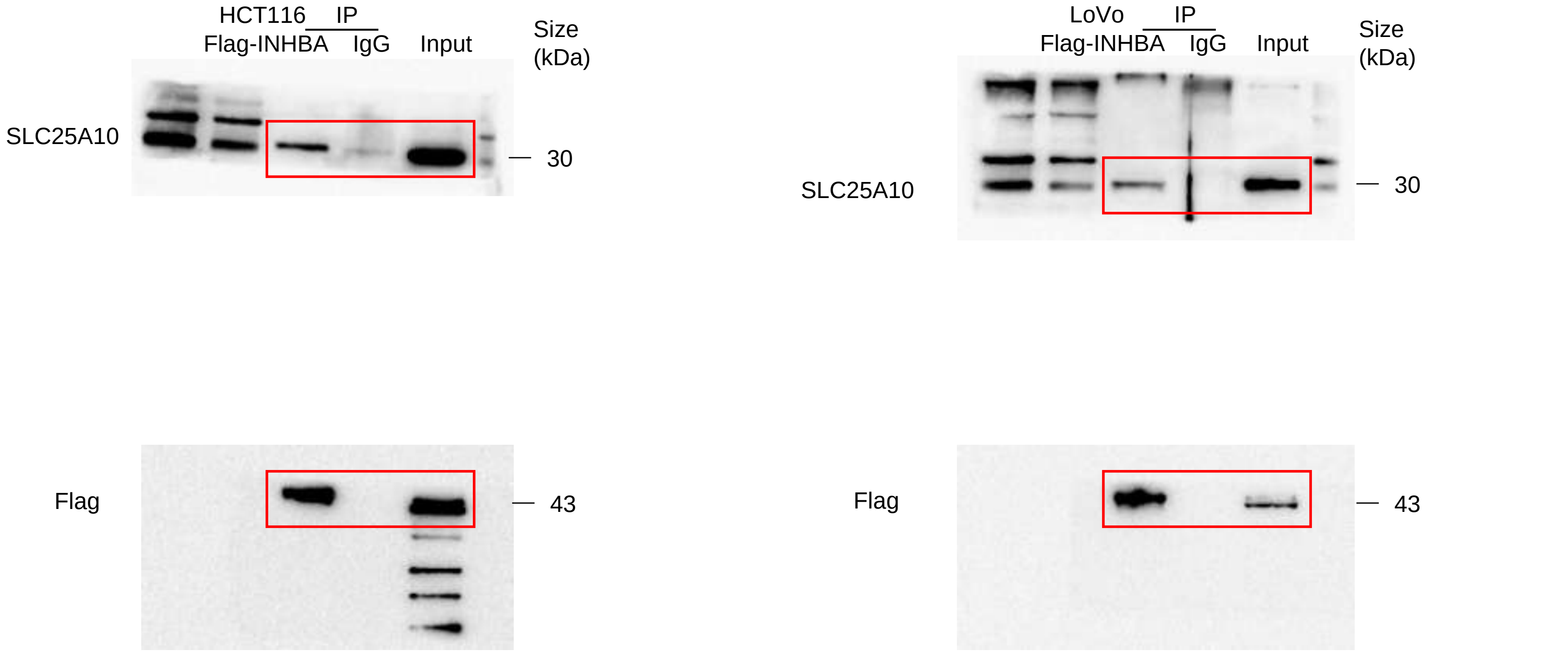
HCT116
IP
Flag-INHBA
IgG
Input
Size (kDa)
SLC25A10
30
Flag
43
LoVo
IP
Flag-INHBA
IgG
Input
Size (kDa)
SLC25A10
30
Flag
43

**Fig. S21b**

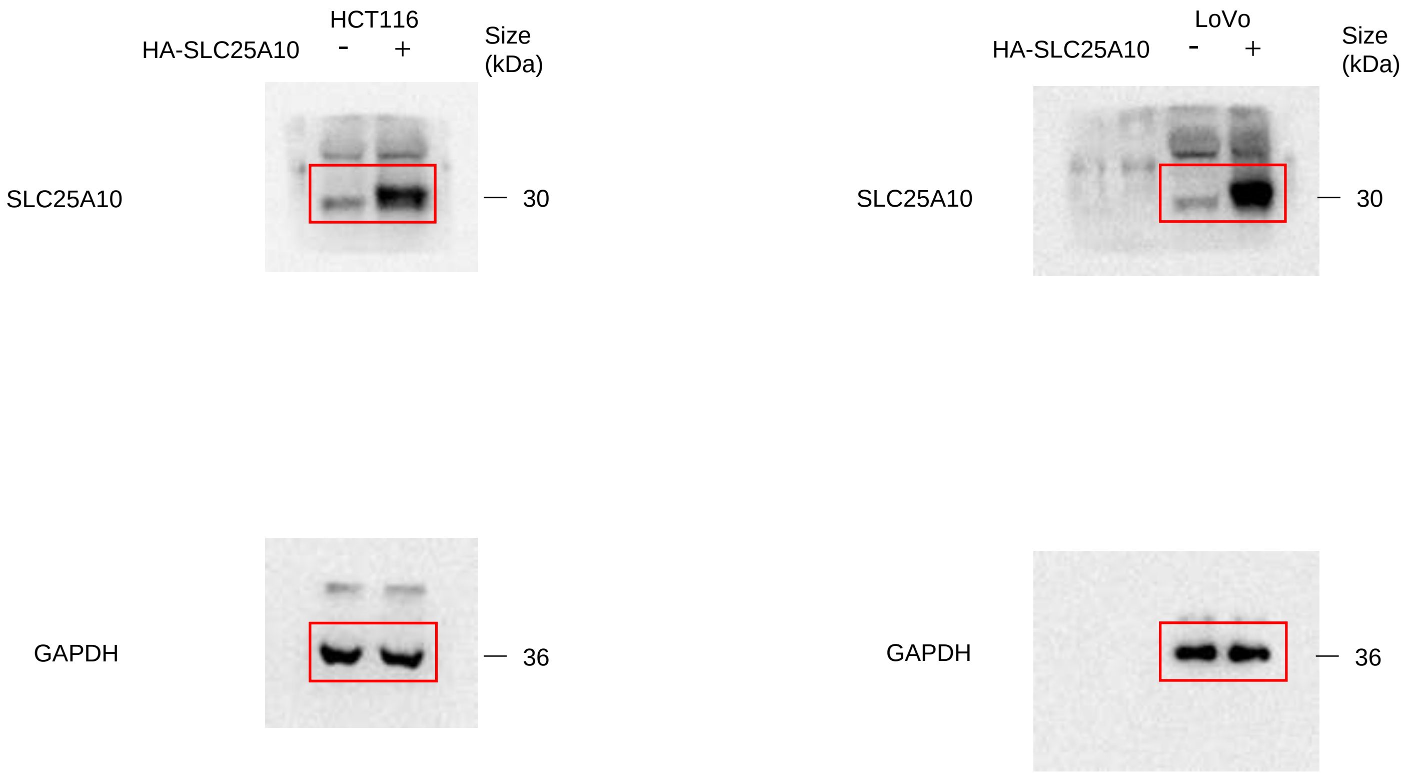
HCT116
HA-SLC25A10 - +
Size (kDa)
SLC25A10
30
GAPDH
36
LoVo
HA-SLC25A10 - +
Size (kDa)
SLC25A10
30
GAPDH
36

**Fig. S22**

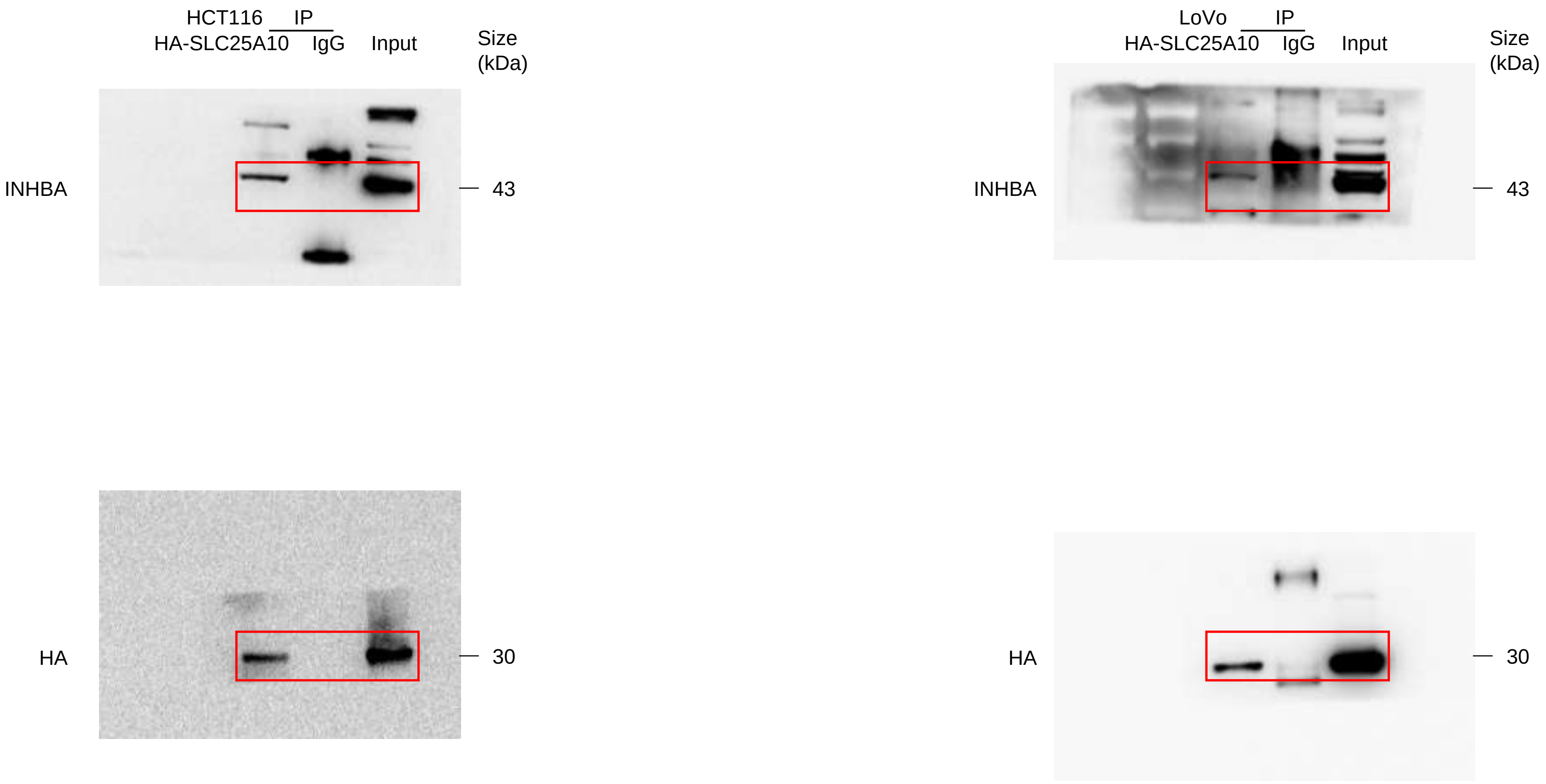
HCT116
IP
HA-SLC25A10
IgG
Input
Size (kDa)
INHBA
43
HA
30
LoVo
IP
HA-SLC25A10
IgG
Input
Size (kDa)
INHBA
43
HA
30

Fig. S29a

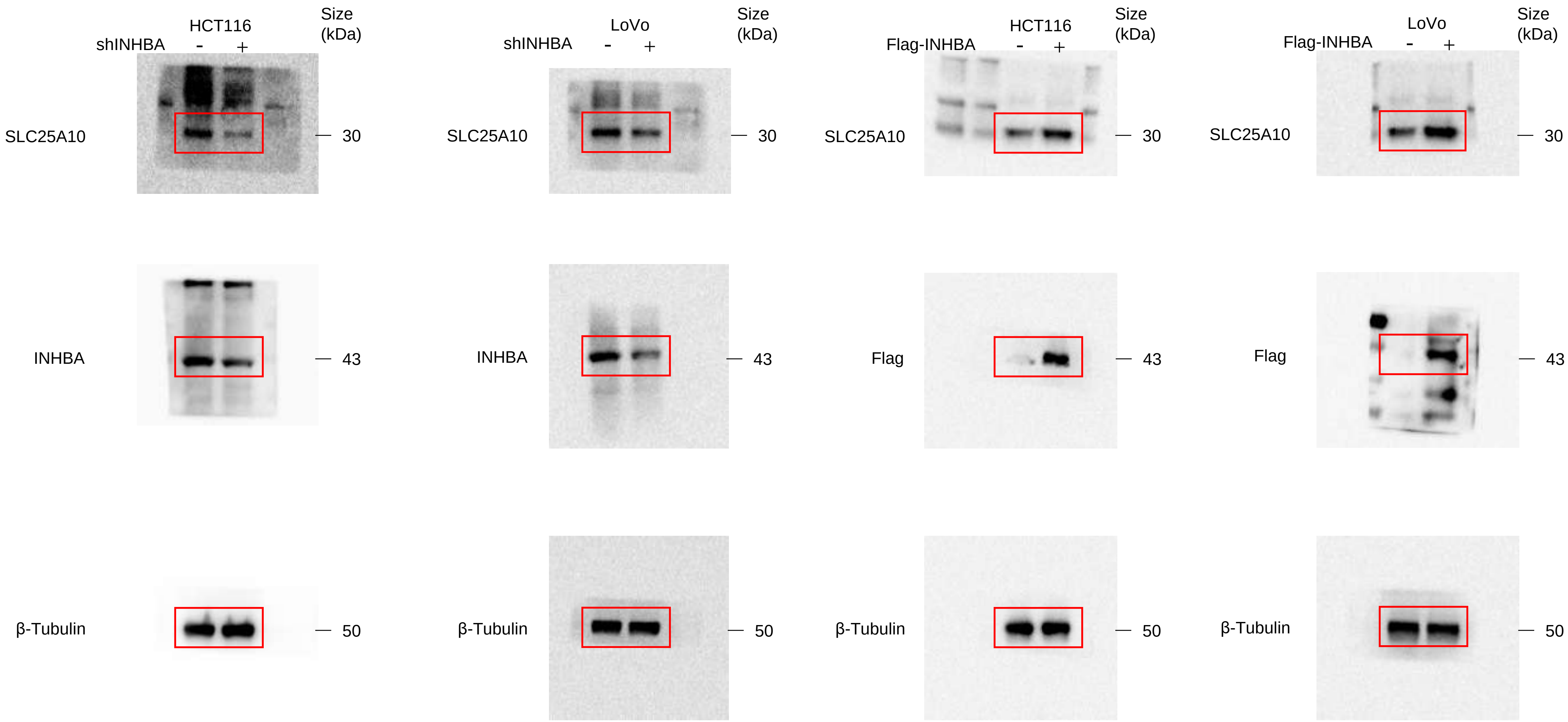
HCT116
shINHBA
-
+
Size (kDa)
SLC25A10
30
INHBA
43
β-Tubulin
50
LoVo
shINHBA
-
+
Size (kDa)
SLC25A10
30
INHBA
43
β-Tubulin
50
HCT116
Flag-INHBA
-
+
Size (kDa)
SLC25A10
30
Flag
43
β-Tubulin
50
LoVo
Flag-INHBA
-
+
Size (kDa)
SLC25A10
30
Flag
43
β-Tubulin
50

**Fig. S29b**

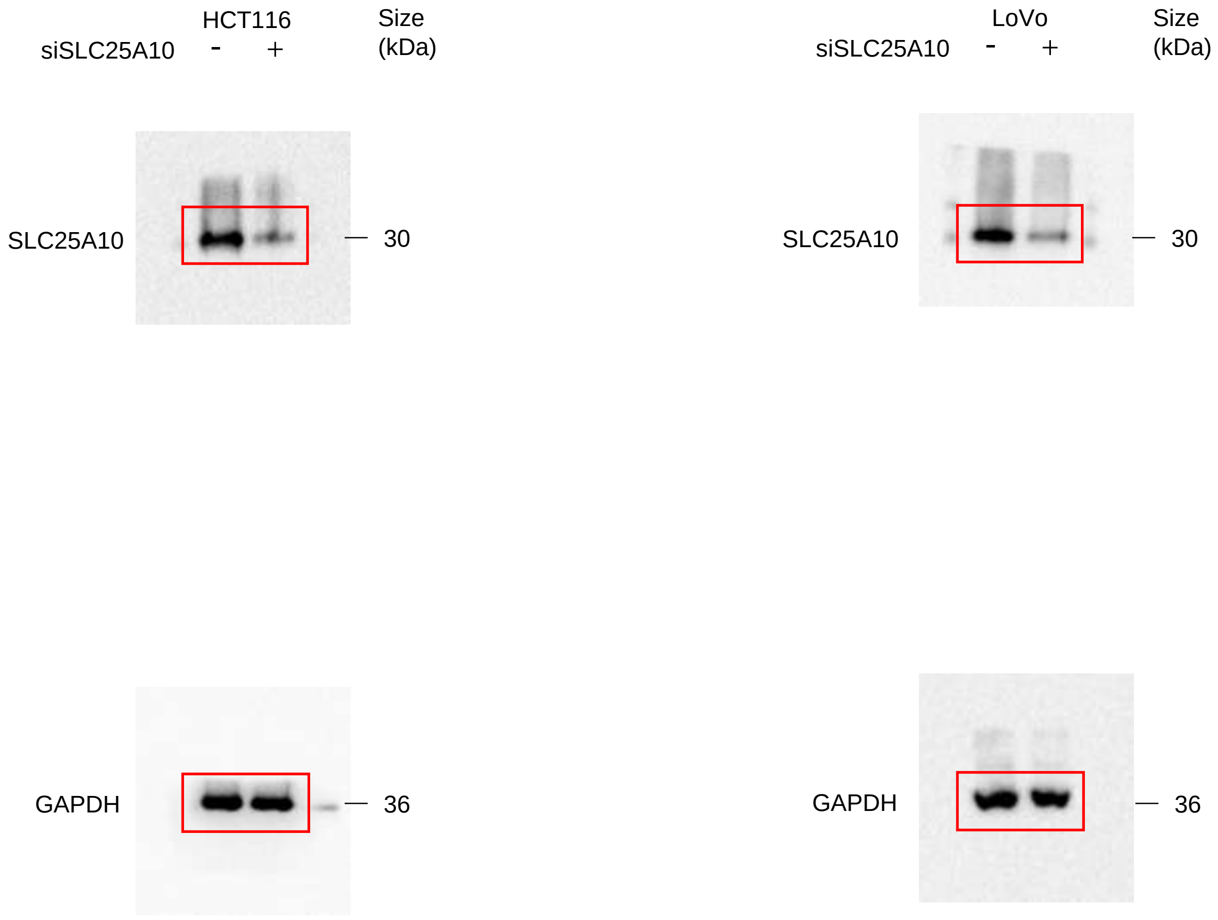

HCT116
siSLC25A10 - +
Size
(kDa)
SLC25A10
30
GAPDH
36
LoVo
siSLC25A10 - +
Size
(kDa)
SLC25A10
30
GAPDH
36

**Fig. S29c**

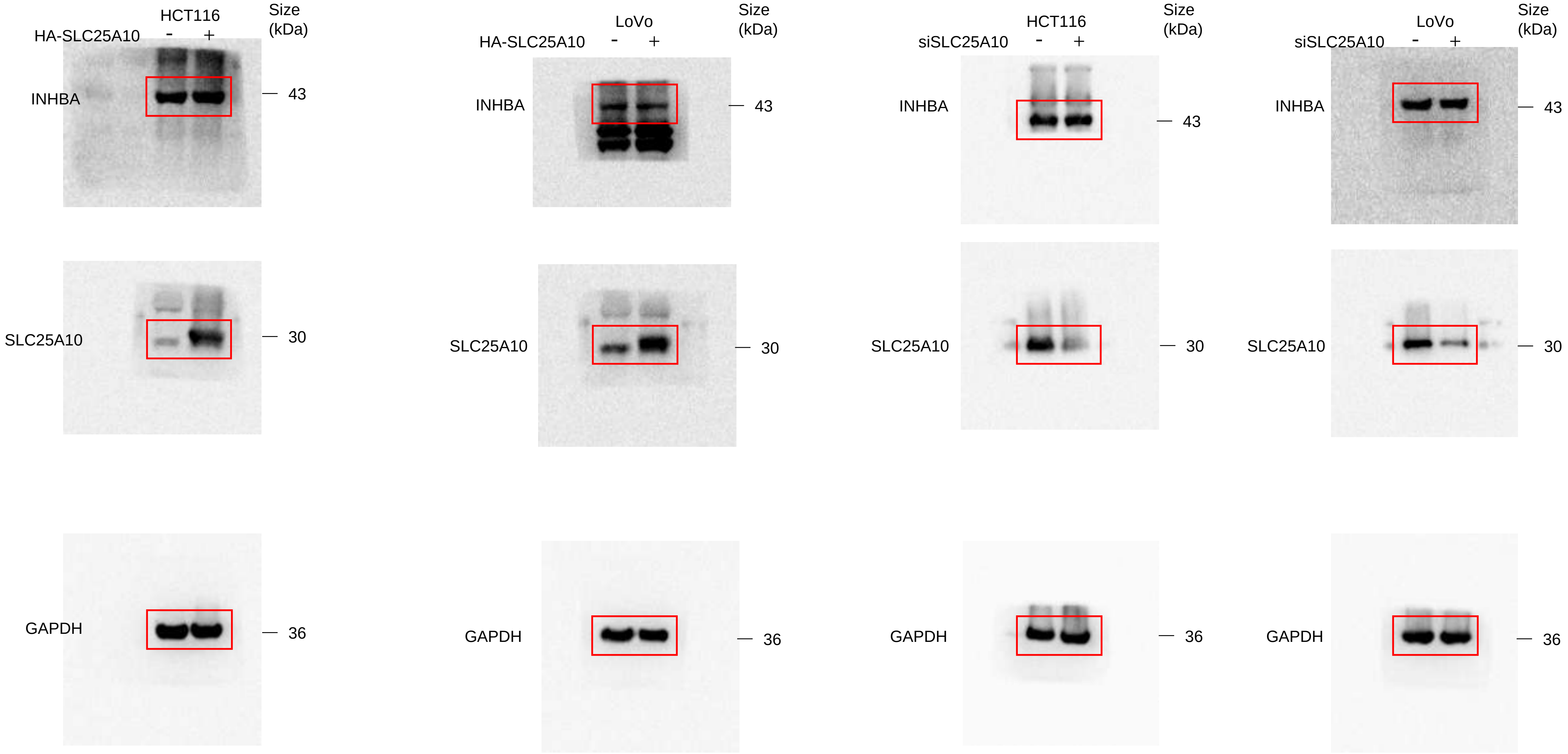
HCT116
HA-SLC25A10 - +
Size (kDa)
INHBA
43
SLC25A10
30
GAPDH
36
LoVo
HA-SLC25A10 - +
Size (kDa)
INHBA
43
SLC25A10
30
GAPDH
36
HCT116
siSLC25A10 - +
Size (kDa)
INHBA
43
SLC25A10
30
GAPDH
36
LoVo
siSLC25A10 - +
Size (kDa)
INHBA
43
SLC25A10
30
GAPDH
36

Fig. S29d

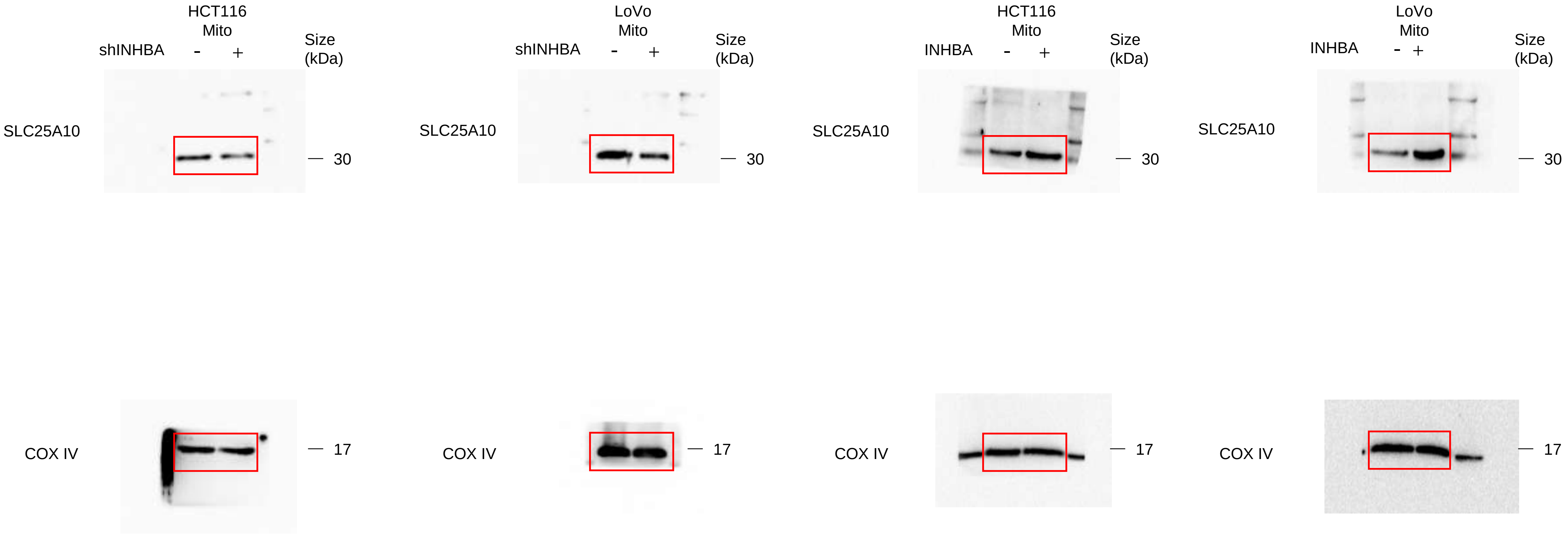
HCT116
Mito
shINHBA
-
+
Size
(kDa)
SLC25A10
30
COX IV
17
LoVo
Mito
shINHBA
-
+
Size
(kDa)
SLC25A10
30
COX IV
17
HCT116
Mito
INHBA
-
+
Size
(kDa)
SLC25A10
30
COX IV
17
LoVo
Mito
INHBA
-
+
Size
(kDa)
SLC25A10
30
COX IV
17

**Fig. S34b**

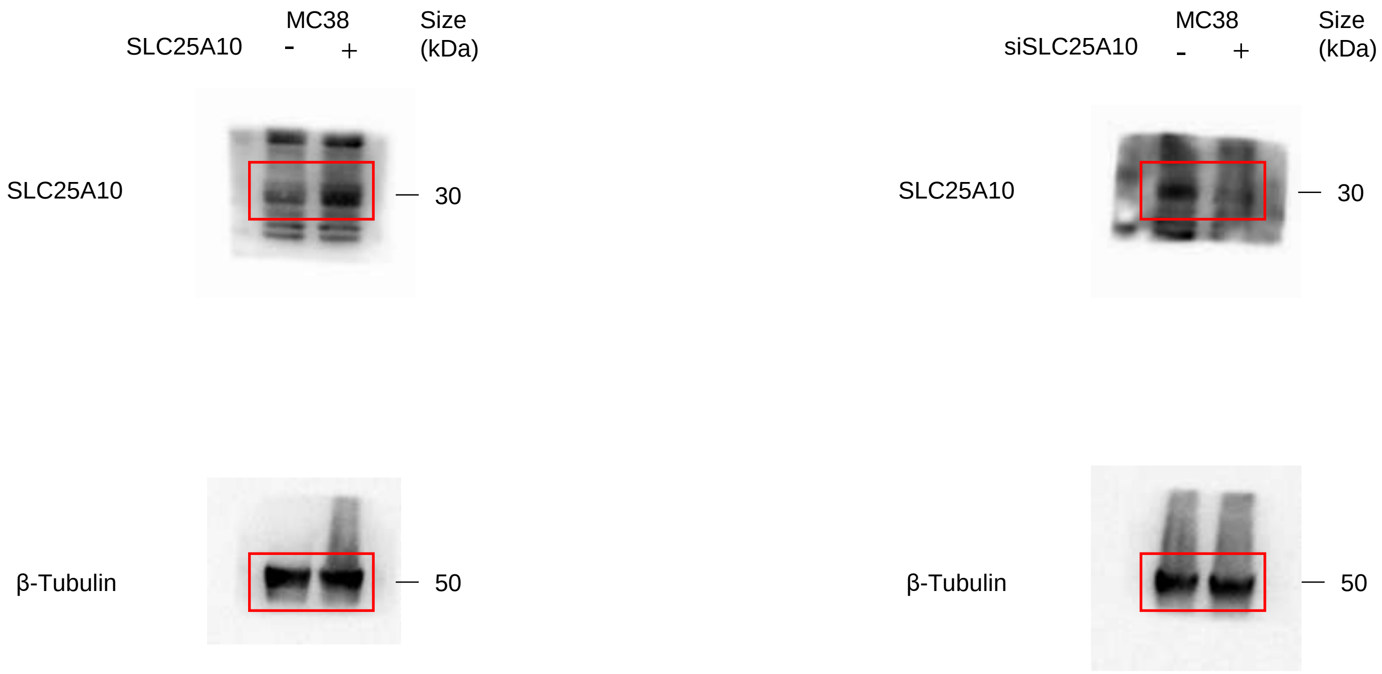
MC38
SLC25A10 - +
Size (kDa)
SLC25A10
30
β-Tubulin
50
MC38
siSLC25A10 - +
Size (kDa)
SLC25A10
30
β-Tubulin
50

**Fig. S35b**

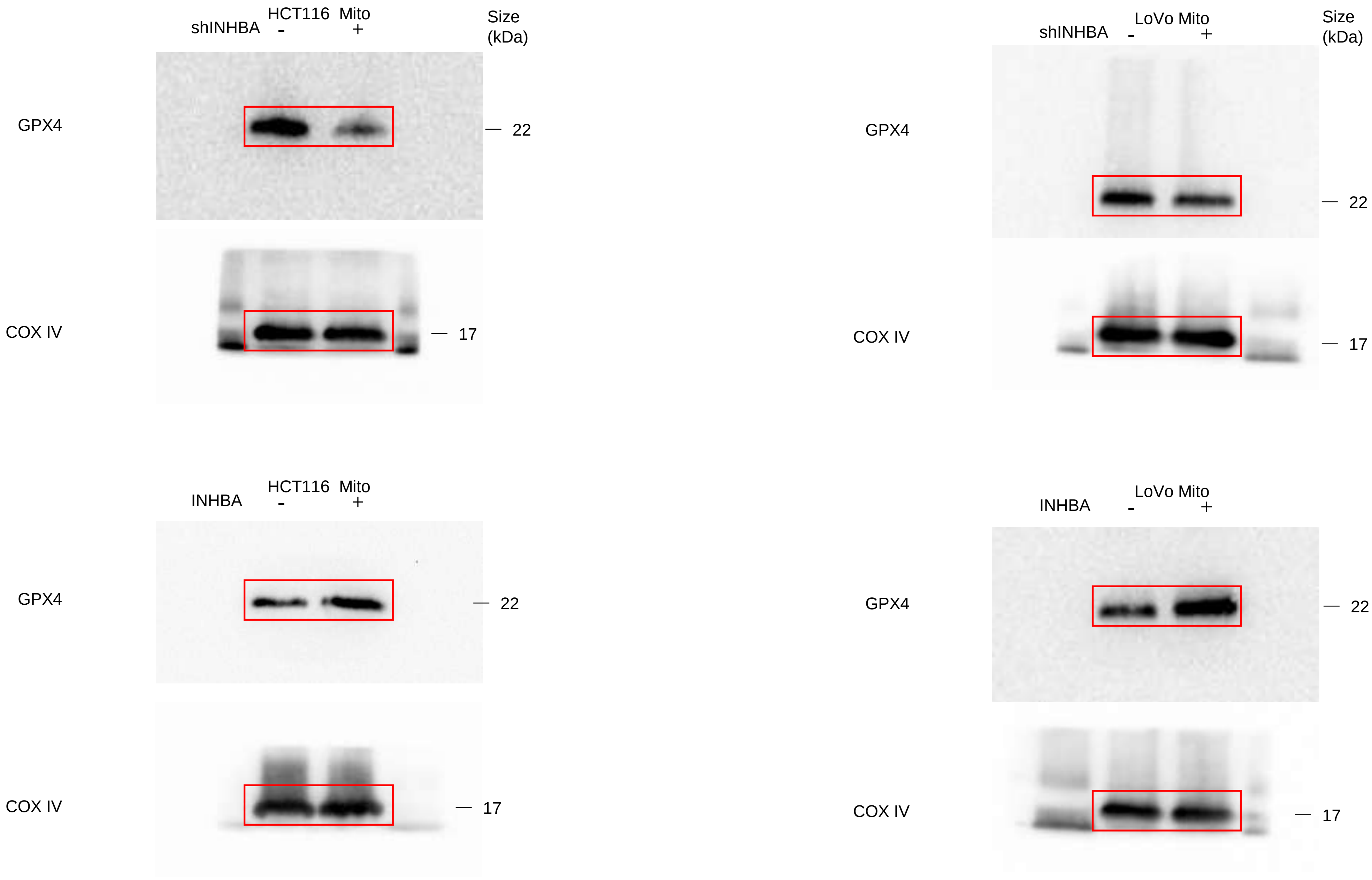
shINHBA
HCT116 Mito
- +
Size (kDa)
GPX4
22
COX IV
17
shINHBA
LoVo Mito
- +
Size (kDa)
GPX4
22
COX IV
17
INHBA
HCT116 Mito
- +
GPX4
22
COX IV
17
INHBA
LoVo Mito
- +
GPX4
22
COX IV
17

Fig. S39

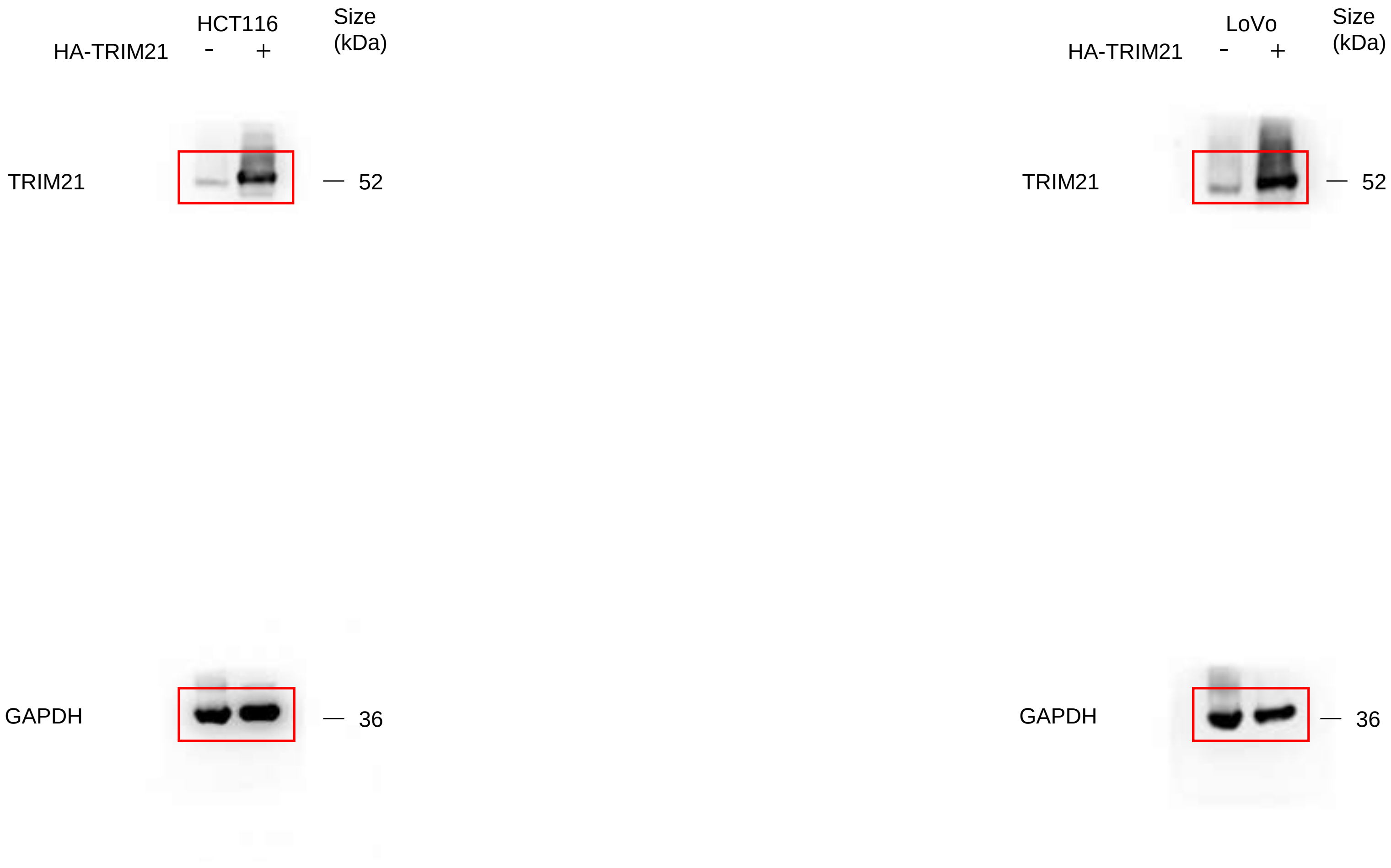
HCT116
HA-TRIM21
-
+
Size (kDa)
TRIM21
52
GAPDH
36
LoVo
HA-TRIM21
-
+
Size (kDa)
TRIM21
52
GAPDH
36

**Fig. S40a**

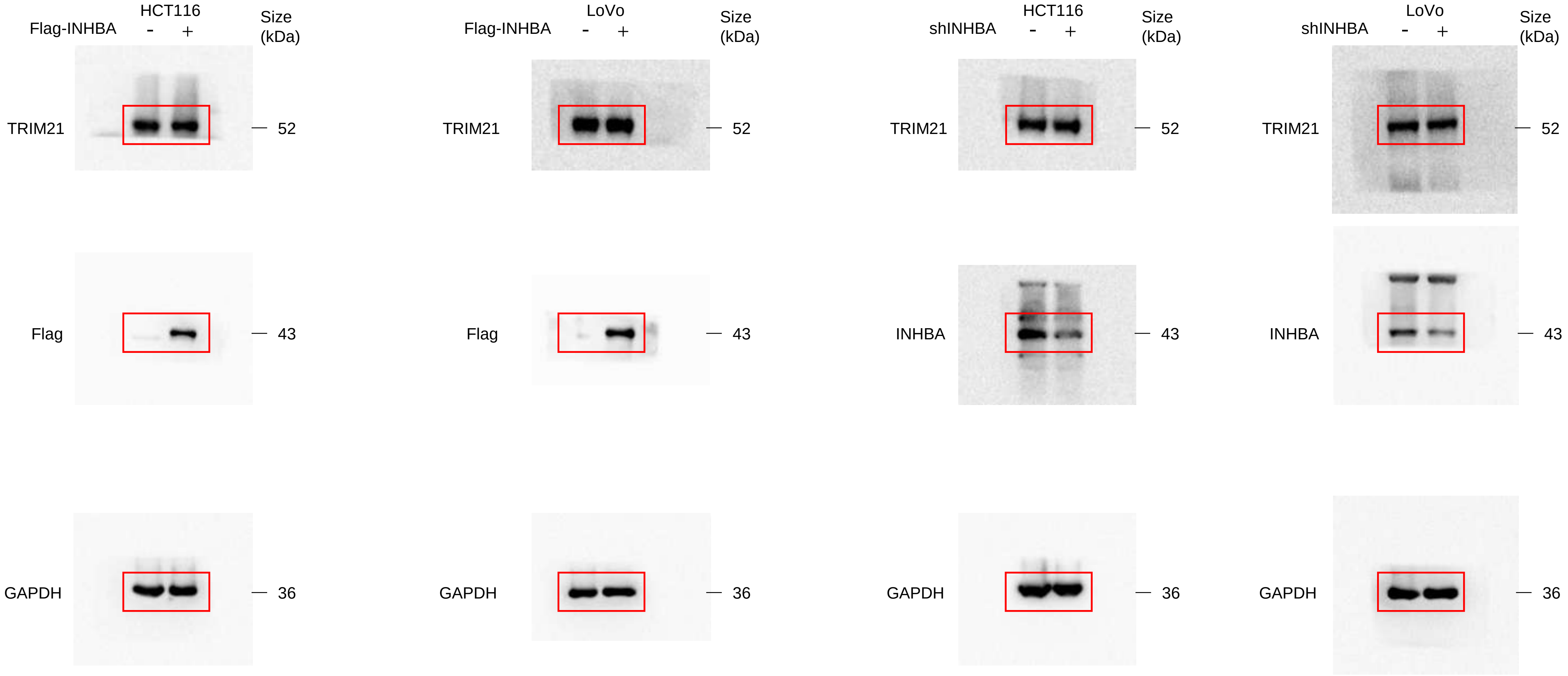

Flag-INHBA
HCT116
- +
Size (kDa)
TRIM21
52
Flag
43
GAPDH
36
Flag-INHBA
LoVo
- +
Size (kDa)
TRIM21
52
Flag
43
GAPDH
36
shINHBA
HCT116
- +
Size (kDa)
TRIM21
52
INHBA
43
GAPDH
36
shINHBA
LoVo
- +
Size (kDa)
TRIM21
52
INHBA
43
GAPDH
36

## Fig. S40b

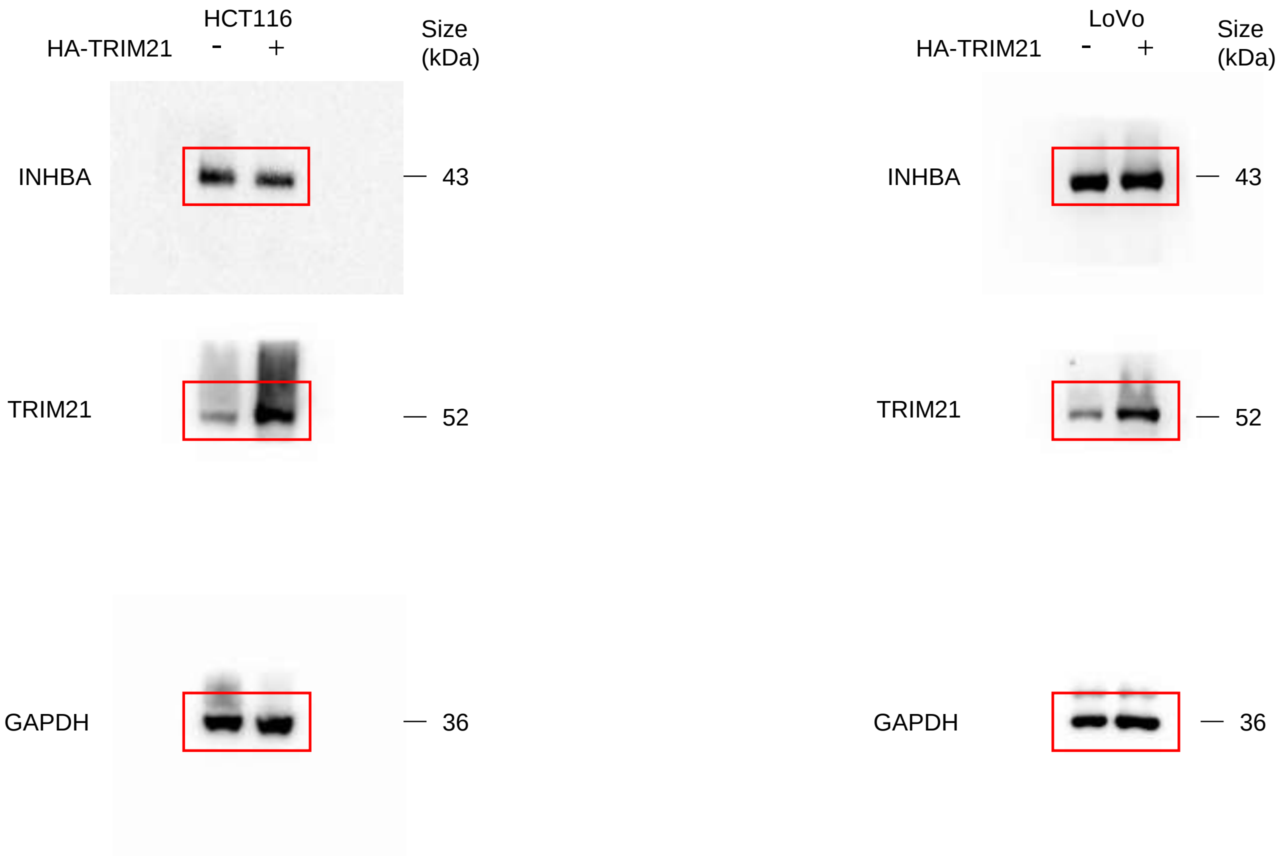
HCT116
HA-TRIM21 - +
Size (kDa)
INHBA
43
TRIM21
52
GAPDH
36
LoVo
HA-TRIM21 - +
Size (kDa)
INHBA
43
TRIM21
52
GAPDH
36

**Fig. S40c**

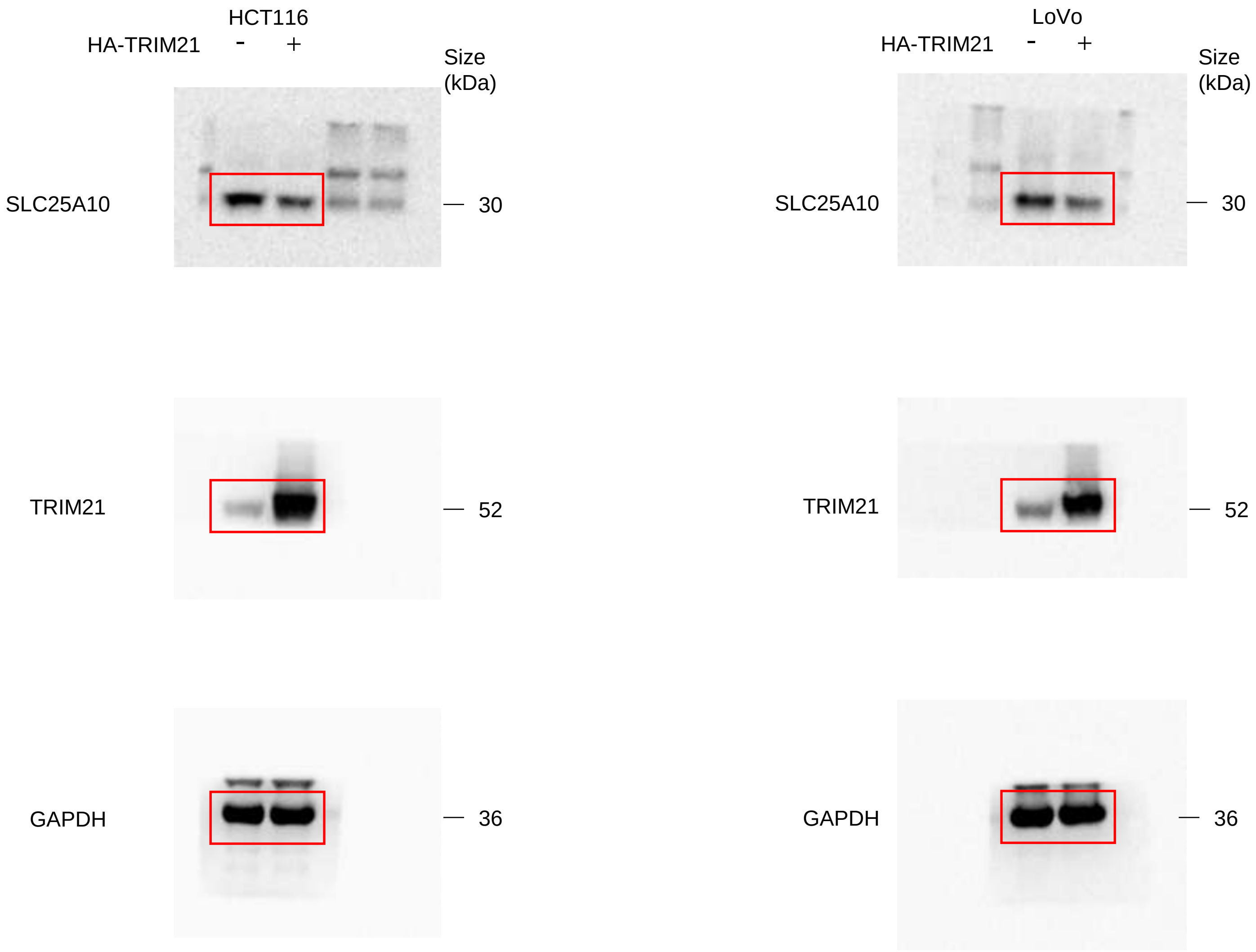

HCT116
HA-TRIM21 - +
Size (kDa)
SLC25A10
30
TRIM21
52
GAPDH
36
LoVo
HA-TRIM21 - +
Size (kDa)
SLC25A10
30
TRIM21
52
GAPDH
36

**Fig. S40d**

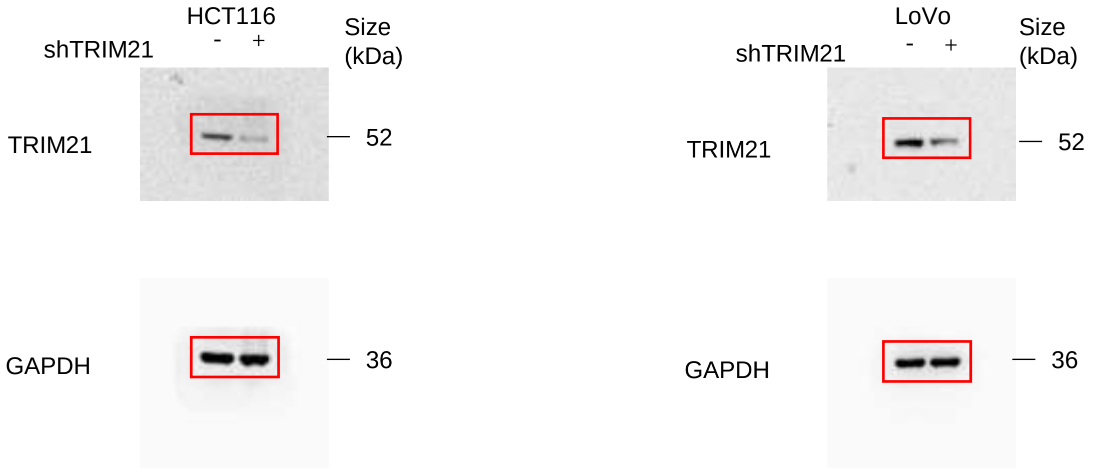


**Fig. S40e**

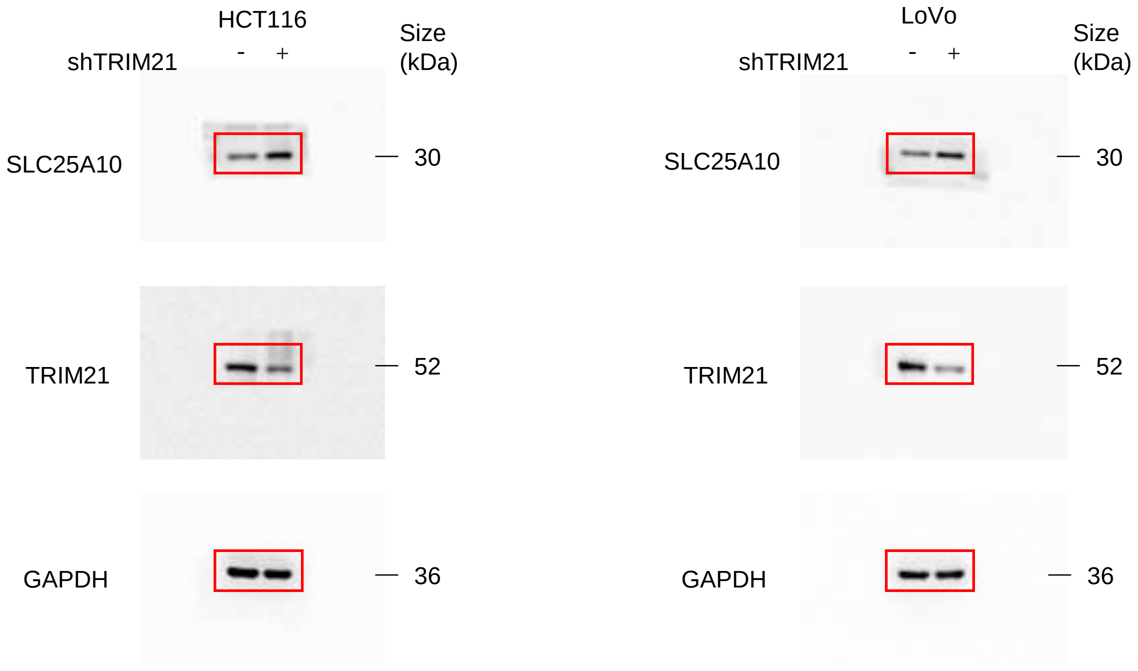

**Fig. S40f**

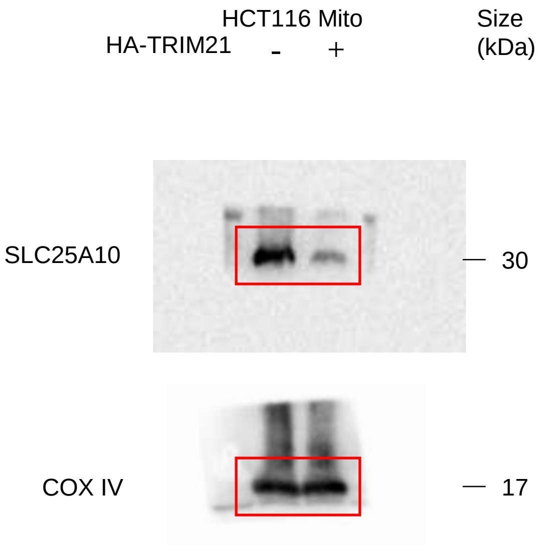
HCT116 Mito
HA-TRIM21
-
+
Size (kDa)
SLC25A10
30
COX IV
17

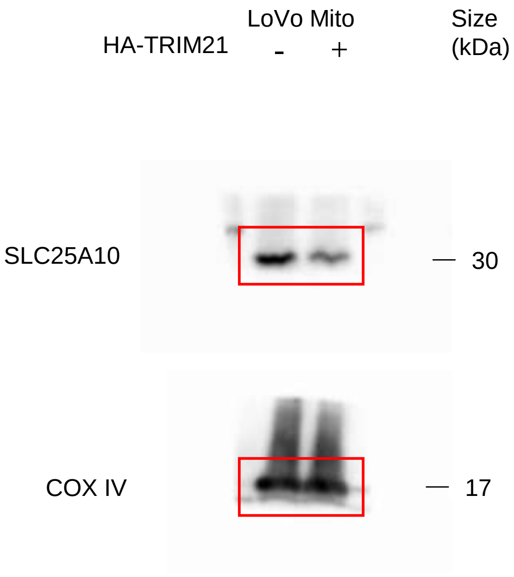
LoVo Mito
HA-TRIM21
-
+
Size (kDa)
SLC25A10
30
COX IV
17

**Fig. S40g**

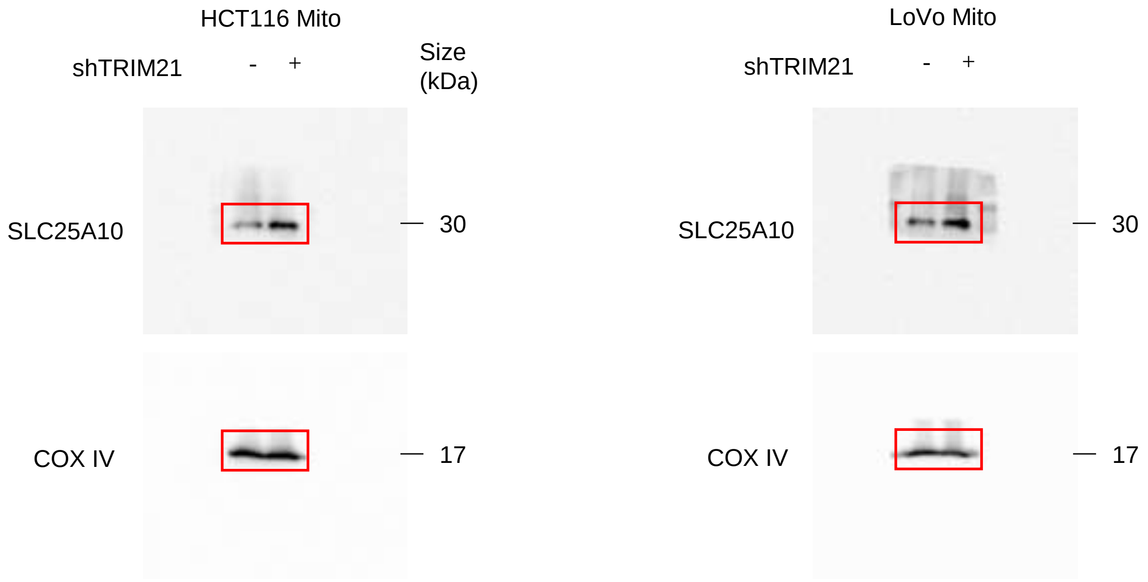
HCT116 Mito
shTRIM21
-
+
Size (kDa)
SLC25A10
30
COX IV
17
LoVo Mito
shTRIM21
-
+
SLC25A10
30
COX IV
17

**Fig. S40h**

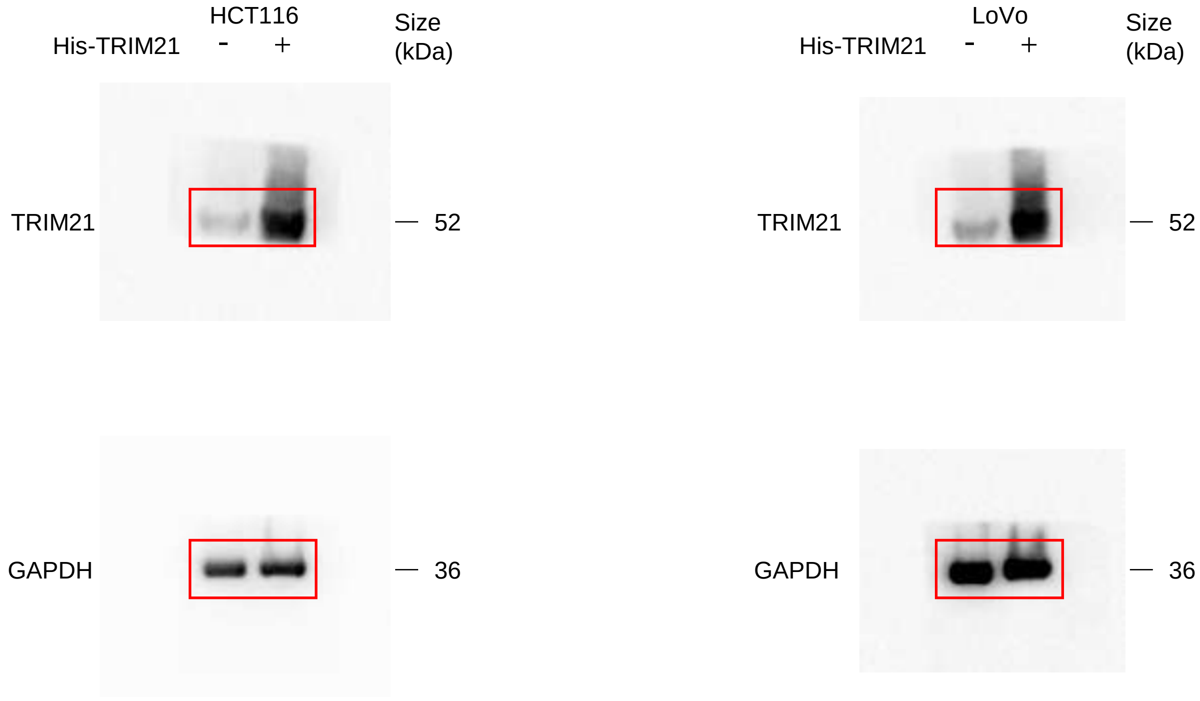
HCT116
His-TRIM21 - +
Size (kDa)
TRIM21
52
GAPDH
36
LoVo
His-TRIM21 - +
Size (kDa)
TRIM21
52
GAPDH
36

**Fig. S41a**

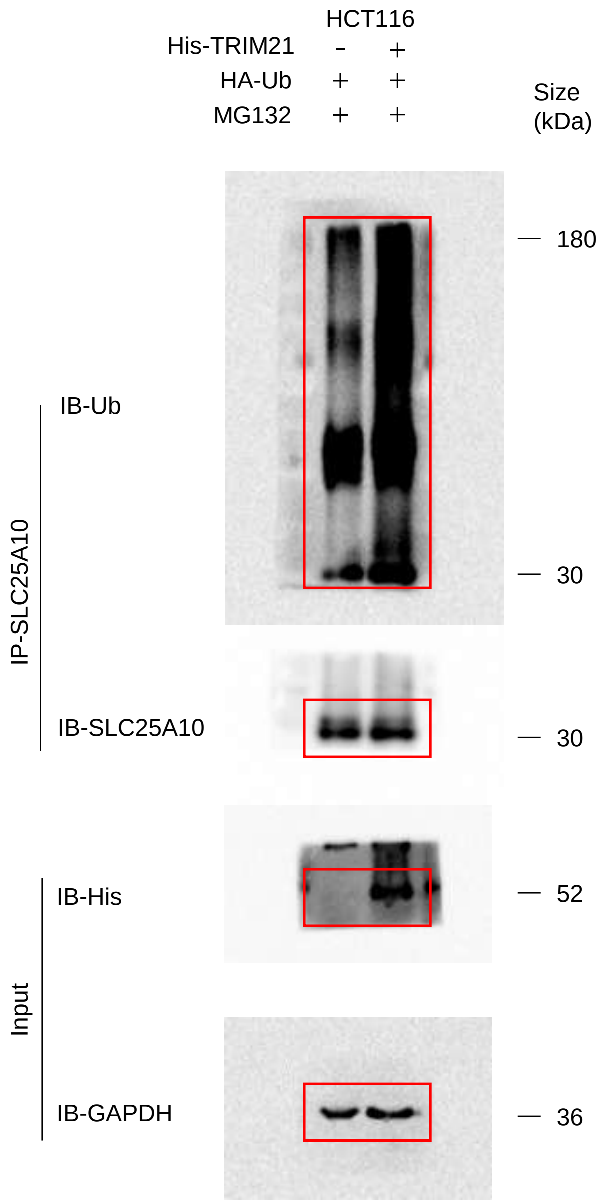
HCT116
His-TRIM21 - +
HA-Ub + +
MG132 + +
Size (kDa)
IB-Ub
IP-SLC25A10
IB-SLC25A10
IB-His
Input
IB-GAPDH
180
30
30
52
36

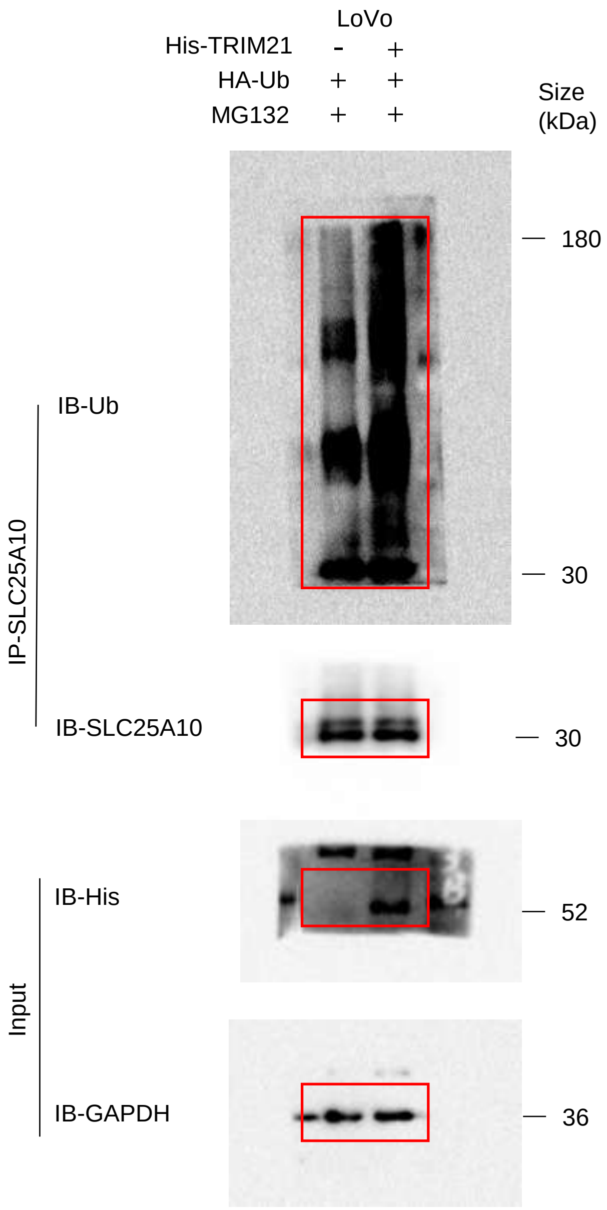
LoVo
His-TRIM21 - +
HA-Ub + +
MG132 + +
Size (kDa)
IB-Ub
IP-SLC25A10
IB-SLC25A10
IB-His
Input
IB-GAPDH
180
30
30
52
36

**Fig. S41b**

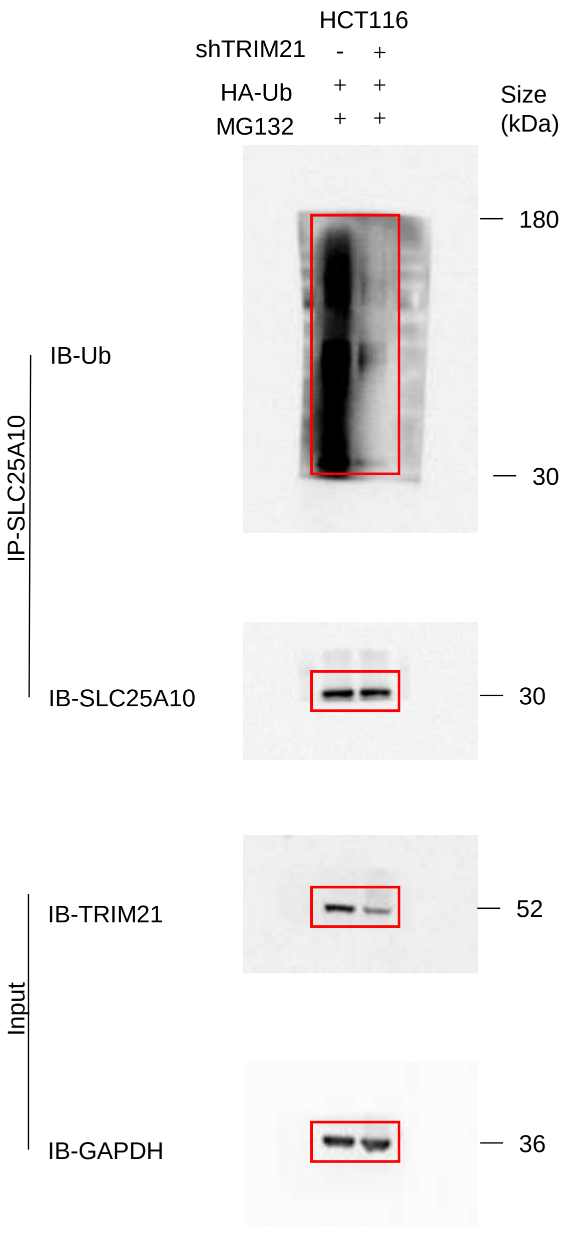
HCT116
shTRIM21 - +
HA-Ub + +
MG132 + +
Size (kDa)
IB-Ub
IP-SLC25A10
IB-SLC25A10
IB-TRIM21
Input
IB-GAPDH
180
30
30
52
36

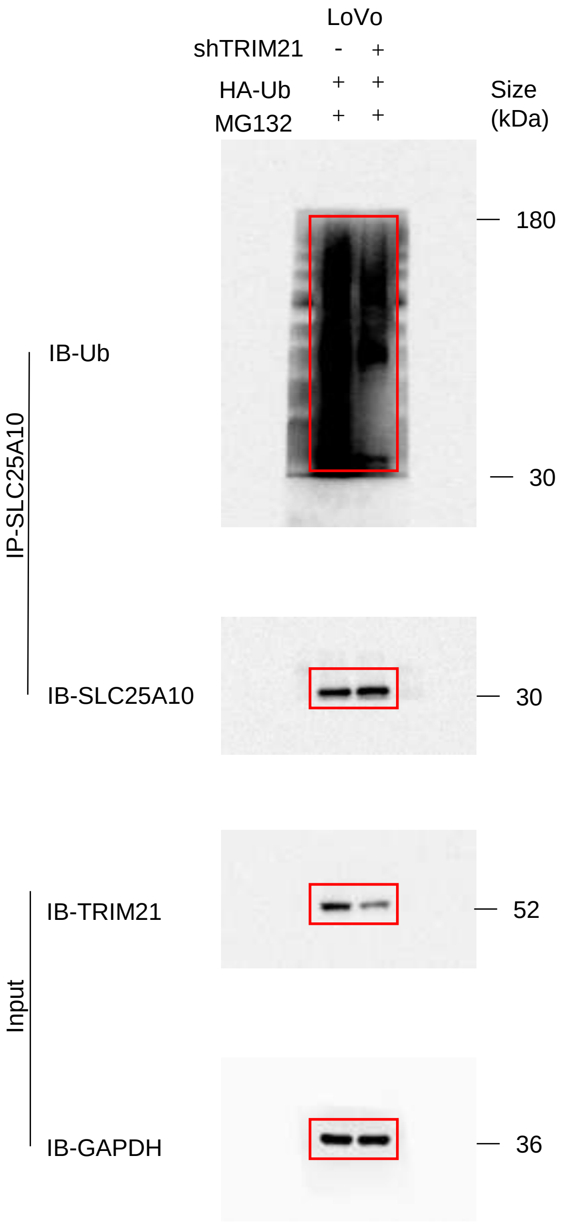
LoVo
shTRIM21 - +
HA-Ub + +
MG132 + +
Size (kDa)
IB-Ub
IP-SLC25A10
IB-SLC25A10
IB-TRIM21
Input
IB-GAPDH
180
30
30
52
36

**Fig. S42**

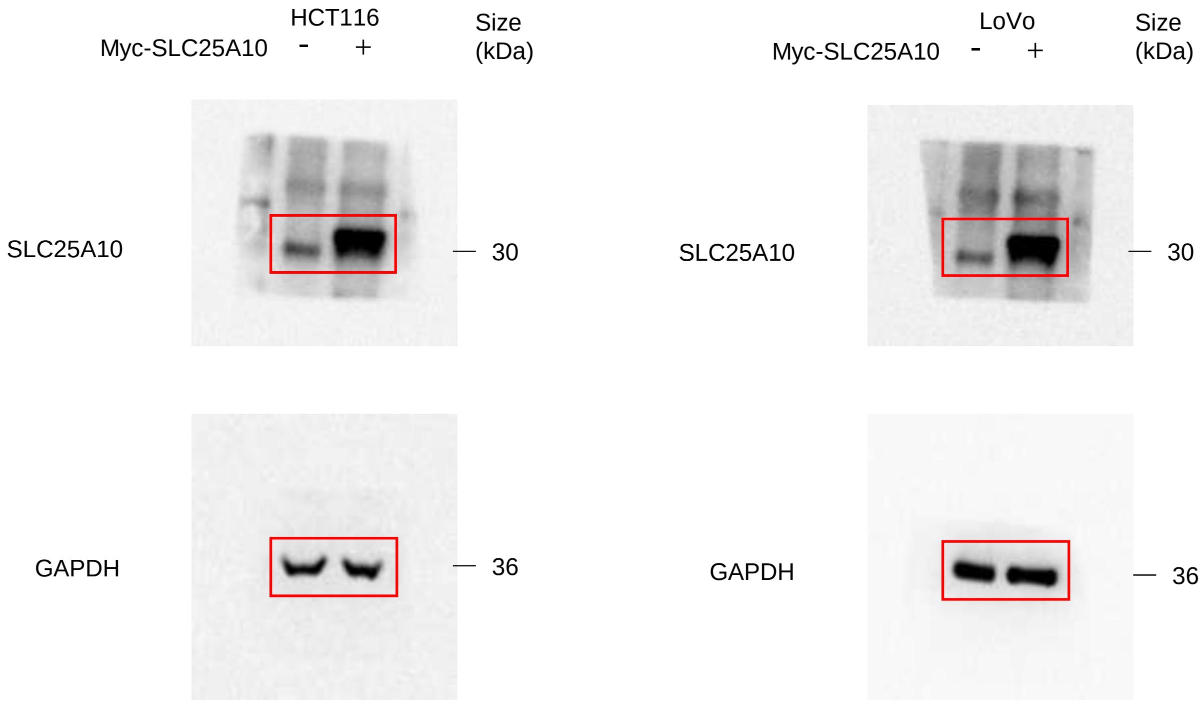

HCT116
Myc-SLC25A10 - +
Size (kDa)
SLC25A10
30
GAPDH
36
LoVo
Myc-SLC25A10 - +
Size (kDa)
SLC25A10
30
GAPDH
36

Fig. S43

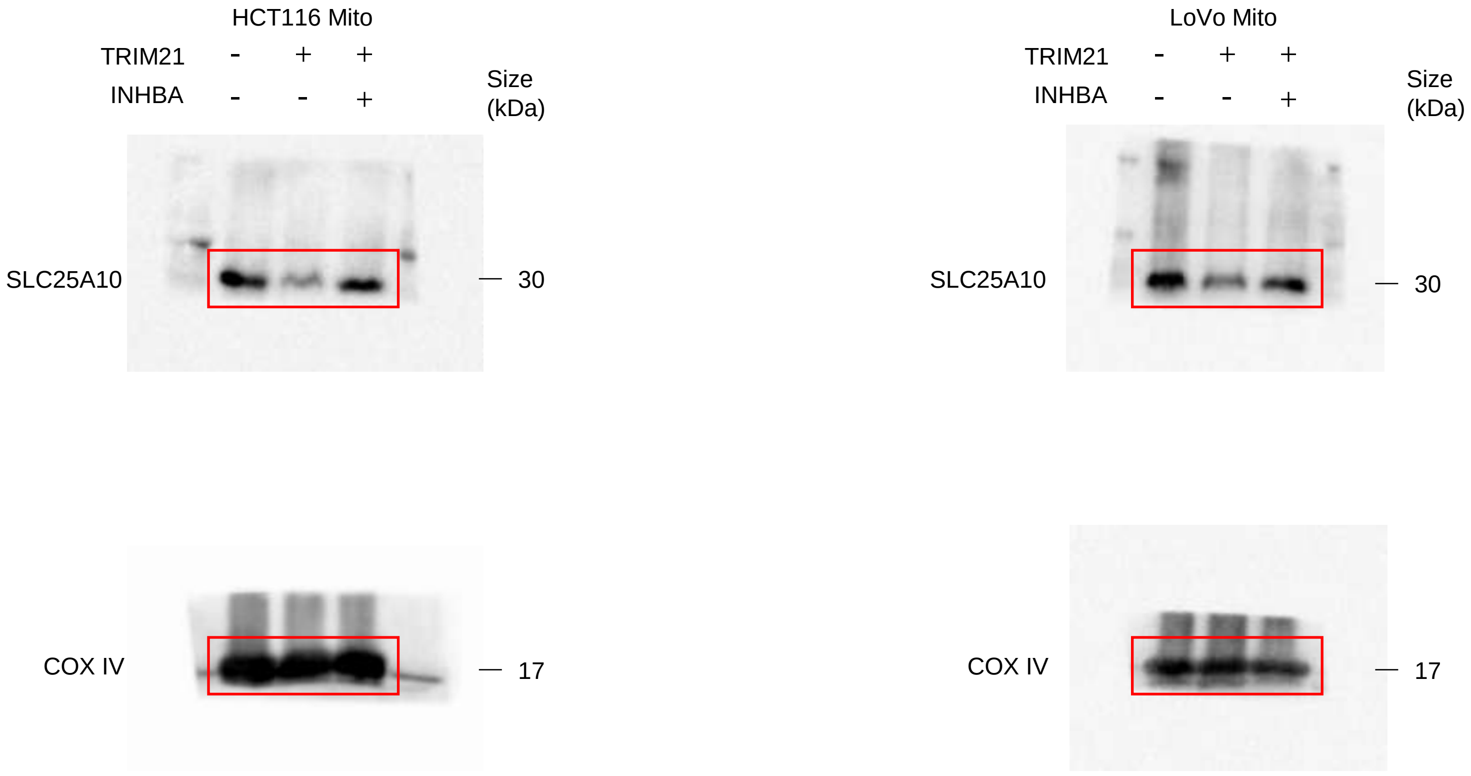
HCT116 Mito
TRIM21 - + +
INHBA - - +
Size (kDa)
SLC25A10
30
COX IV
17
LoVo Mito
TRIM21 - + +
INHBA - - +
Size (kDa)
SLC25A10
30
COX IV
17
